# Synergistic Activation of Immunogenic Cell Death and the cGAS–STING Pathway by Engineered Zinc/Manganese‐Based Metal–Organic Framework Nanoplatforms for Colon Cancer Immunotherapy

**DOI:** 10.1002/advs.202521146

**Published:** 2026-01-28

**Authors:** Bingzi Zhu, Xiaodong Chen, Chang Xu, Xuehua Qian, Binglong Bai, Ji Lin, Wenhai Deng, Xiang Wang, Yuekai Cui, Shengsheng Zhao, Zuoliang Xie, Tao You, Yingpeng Huang, Xian Shen, Xufeng Lu, Weijian Sun

**Affiliations:** ^1^ Department of Colorectal and Anal Surgery, Zhejiang–Finland Joint Laboratory of Gastrointestinal Tumor Metabolism and Nutrition The First Affiliated Hospital of Wenzhou Medical University Wenzhou Zhejiang China; ^2^ Department of Gastrointestinal Surgery, Zhejiang Key Laboratory of Intelligent Cancer Biomarker Discovery and Translation The First Affiliated Hospital Wenzhou Medical University Wenzhou Zhejiang China; ^3^ Department of Gastrointestinal Surgery Zhejiang International Scientific and Technological Cooperation Base of Translational Cancer Research The Second Affiliated Hospital and Yuying Children's Hospital of Wenzhou Medical University Wenzhou Zhejiang China; ^4^ Department of Information The First Affiliated Hospital of Wenzhou Medical University Wenzhou Zhejiang China; ^5^ Department of Hepatobiliary and Pancreatic Surgery The Second Affiliated Hospital and Yuying Children's Hospital of Wenzhou Medical University Wenzhou Zhejiang China; ^6^ Oujiang Laboratory (Zhejiang Lab For Regenerative Medicine, Vision, and Brain Health) School of Laboratory Medicine and Life Sciences Wenzhou Medical University Wenzhou Zhejiang China; ^7^ Research Center of Basic Medicine The Second Affiliated Hospital and Yuying Children's Hospital of Wenzhou Medical University Wenzhou Zhejiang China

**Keywords:** c‐di‐AMP diammonium, cGAS–STING pathway, colon cancer, immunogenic cell death (ICD), Zn/Mn‐MOF

## Abstract

The cyclic guanosine monophosphate (GMP)–adenosine monophosphate (AMP) synthase–stimulator of interferon genes (cGAS–STING) pathway is crucial for tumor immunity. However, activation of the cGAS–STING pathway alone is seldom sufficient to eliminate established tumors. Here, we report the engineering of zinc/manganese (Zn/Mn)‐based metal–organic framework (MOF) nanoparticles, that is, AMP@Zn/Mn‐MOF, comprising Zn/Mn‐MOF nanoparticles as the carrier and the STING agonist c‐di‐AMP diammonium as the therapeutic drug for reinforcing antitumor immune responses. These therapeutic nanoplatforms can significantly activate the cGAS‒STING pathway and facilitate the innate immune response. Furthermore, the peroxidase (POD)‐mimetic and glutathione oxidase (GSHox)‐mimetic activities of AMP@Zn/Mn‐MOF can significantly potentiate tumor cell death and effectively induce robust immunogenic cell death (ICD), thereby amplifying the cGAS–STING pathway. Moreover, AMP@Zn/Mn‐MOF reprogrammed the immunosuppressive tumor microenvironment by promoting intratumoral lymphocyte infiltration, thereby significantly suppressing the growth of murine MC38 tumors in mice. Notably, AMP@Zn/Mn‐MOF amplified the therapeutic effect of anti‐programmed death ligand 1 (αPD‐L1) blockade by triggering systemic antitumor responses, resulting in a notable abscopal effect to effectively inhibit distant tumors. In summary, AMP@Zn/Mn‐MOF offers a nanoplatform with enhanced antitumor effectiveness through activation of the cGAS–STING pathway and ICD, suggesting that enhanced immune checkpoint blockade‐based immunotherapy is promising for colon cancer treatment.

## Graphical abstract

A zinc/manganese (Zn/Mn)‐based metal‐organic framework (MOF) loaded with the STING agonist c‐di‐AMP diammonium (denoted AMP@Zn/Mn‐MOF) was developed to synergistically activate the cGAS‐STING pathway and induce immunogenic cell death. This nanoplatform reprograms the immunosuppressive tumor microenvironment, significantly enhancing anti‐PD‐L1 immunotherapy and eliciting abscopal effects against colon cancer.

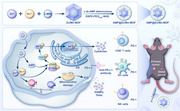

## 1 Introduction

Colon cancer (CC) ranks as the third most common malignancy and the second leading cause of cancer‐related death worldwide [1, 2]. Currently, surgical resection followed by postoperative chemotherapy is the primary approach for managing CC, with a 5‐year survival rate of approximately 60% [1, 3, 4]. Despite significant progress in the field of CC research, the development of new therapeutic strategies is urgently needed. In recent years, immune checkpoint blockade (ICB) agents, including cytotoxic T‐lymphocyte antigen‐4 (CTLA‐4), programmed death receptor‐1 (PD‐1), and programmed death ligand 1 (PD‐L1), have achieved revolutionary breakthroughs in the treatment of CC [5]. Unfortunately, one of the main limitations in the application of immunotherapy is the immunosuppressive tumor microenvironment (TME) [6]. In addition, clinical studies have shown that only a minority of CC patients achieve long‐term benefits from PD‐1/PD‐L1‐based therapy [5, 7]. Therefore, ICB is often adopted as an adjunctive strategy to CC treatment in the clinical setting. Within this context, increasing the effectiveness of immunotherapy through the combined application of multiple approaches is crucial for addressing CC.

Activation of innate immunity has been recognized as a highly effective and prominent strategy to increase the effectiveness of tumor immunotherapy [8]. The cyclic guanosine monophosphate (GMP)–adenosine monophosphate (AMP) synthase–stimulator of interferon genes (cGAS–STING) pathway, which is a key component of the innate immune system, has recently emerged as a highly promising target to accelerate tumor therapy [9, 10]. cGAS recognizes double‐stranded (ds) DNA in the cytoplasm and synthesizes cGAMP, which subsequently binds and activates STING and produces type I interferon (IFN‐I) and proinflammatory cytokines, thereby triggering antitumor innate immune responses and providing therapeutic effects [11]. STING agonists have demonstrated prospective therapeutic effectiveness in increasing immune stimulation and promoting antitumor immune responses in CC [12]. Therefore, the continuous activation of the cGAS–STING pathway offers a potential solution for improving the treatment of CC.

Among various nanoformulations, metal–organic frameworks (MOFs), as a type of porous material comprising organic ligands and their coordinating metal ions/ion clusters, have received attention because of their multiple advantages, including facile synthesis, excellent thermal stability, and the ability to achieve targeted drug delivery [13, 14]. MOF nanoparticles have demonstrated potential as effective carriers for small‐molecule drugs, proteins, and oligonucleotides, facilitating their use in chemotherapy, immunotherapy, and radiotherapy [15, 16]. Notably, the development of MOFs won the 2025 Nobel Prize in Chemistry. Recent studies have shown that the accumulation of zinc ions (Zn^2+^) and manganese ions (Mn^2+^) in the cytoplasm increases the activity of cGAMP synthase, leading to increased cGAMP levels and the activation of innate immune responses via a STING‐dependent pathway [17]. However, direct clinical use of Zn^2+^ or Mn^2+^ is precluded because of their significant toxicity and side effects [18]. To address this issue, various Zn^2+^/Mn^2+^‐coordinated nanoparticles have been developed to deliver these ions as agonists within the TME [19], thereby achieving notable antitumor effects. However, studies on the use of Zn^2+^/Mn^2+^‐modified MOFs as nanotherapeutics in immunotherapy are scarce [20]. In addition, numerous natural or synthetic STING agonists have been developed for cancer therapy, with some demonstrating superior effectiveness and tolerability in preclinical trials [21]. However, these agonists still face drawbacks, such as rapid enzymatic degradation and elimination, poor drug delivery targeting, and instability [22]. Although the cGAS–STING pathway has emerged as a promising therapeutic target, effective systemic delivery remains a significant challenge.

**FIGURE 1:**
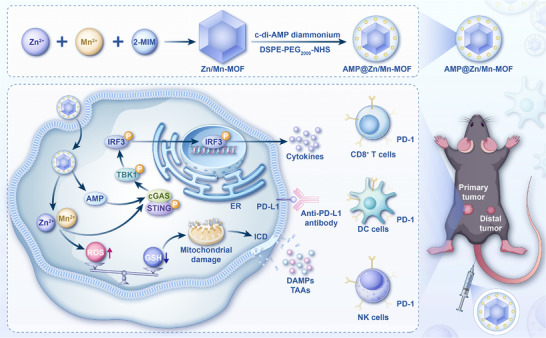
Schematic illustration of AMP@Zn/Mn‐MOF for synergistic activation of the cGAS‐STING pathway and ICD.

To address the above issues, we developed a Zn/Mn‐MOF decorated with a STING agonist c‐di‐AMP diammonium nanoplatform, as illustrated in Figure 1. Notably, AMP@Zn/Mn‐MOF exhibited superior peroxidase (POD)‐mimetic and glutathione oxidase (GSHox)‐mimetic catalytic efficiency, catalyzing the generation of reactive oxygen species (ROS), which in turn triggered immunogenic cell death (ICD) in cancer cells and the release of damage‐associated molecular patterns (DAMPs), thereby increasing cellular immunogenicity. Simultaneously, the release of Zn^2+^/Mn^2+^, together with c‐di‐AMP diammonium, activated the cGAS–STING pathway by facilitating the phosphorylation of STING, TBK1, and IRF3, thereby triggering innate immune responses. Furthermore, AMP@Zn/Mn‐MOF inhibited the growth of MC38 tumors in immunocompetent C57BL/6J mice by activating the cGAS–STING pathway and reversing tumor immunosuppression. When combined with the αPD‐L1 antibody, AMP@Zn/Mn‐MOF effectively reshaped the immunosuppressive TME by promoting the infiltration of cytotoxic T lymphocytes (CTLs), thereby significantly inhibiting the growth of both primary and distant tumors. Collectively, our findings indicate that AMP@Zn/Mn‐MOF serves as an excellent nanomaterial for the precise and effective treatment of CC (Figure 1).

## 2 Results and Discussion

### 2.1 Construction and Characterization of AMP@Zn/Mn‐MOF

**FIGURE 2:**
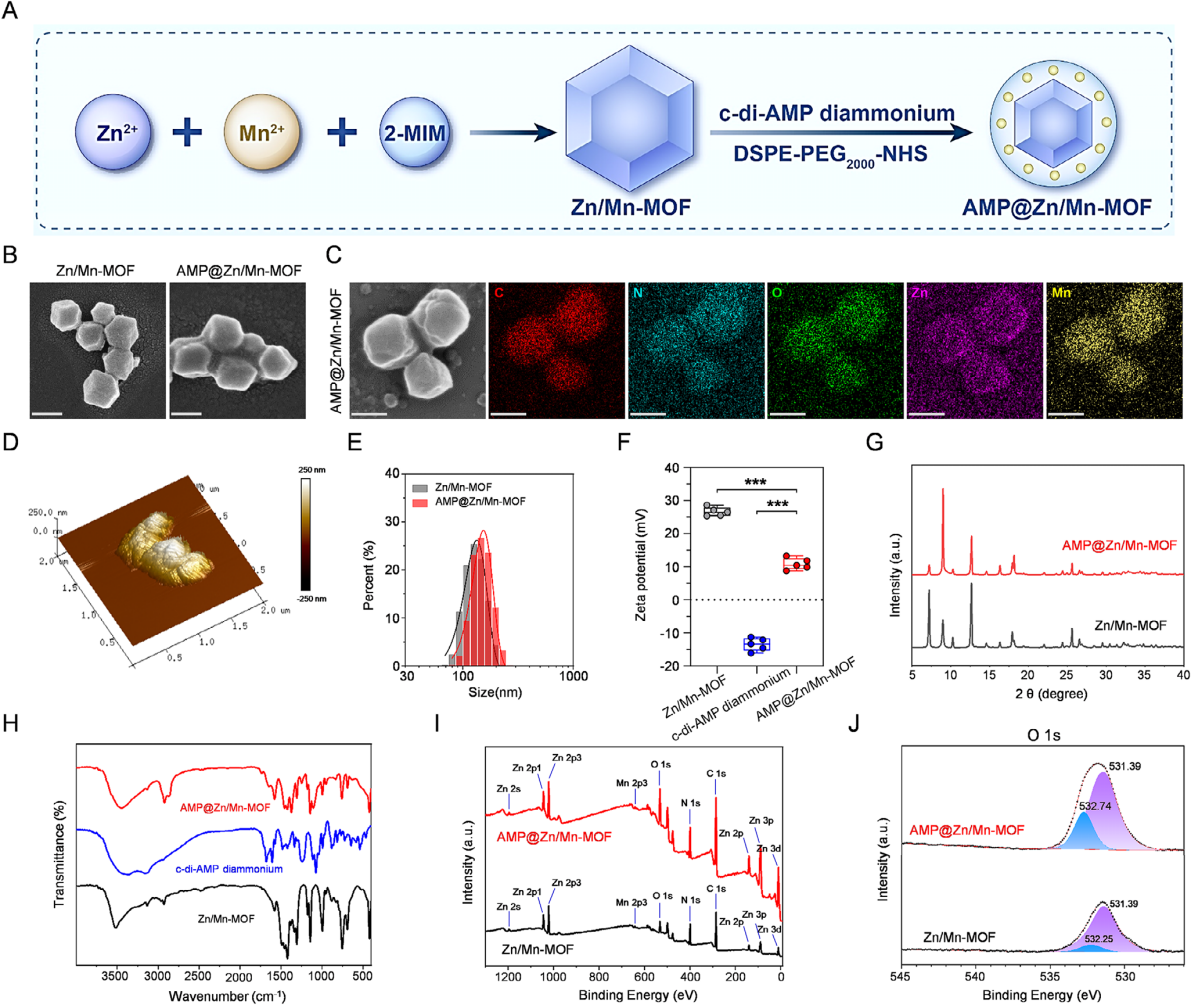
Construction and characterization of AMP@Zn/Mn‐MOF. (A) Schematic diagram of the AMP@Zn/Mn‐MOF synthesis. (B) SEM images of Zn/Mn‐MOF and AMP@Zn/Mn‐MOF. Scale bar: 100 nm. (C) EDS elemental maps of AMP@Zn/Mn‐MOF. Scale bar: 100 nm. (D) AFM characterization of AMP@Zn/Mn‐MOF. (E) Hydrodynamic size distributions of Zn/Mn‐MOF and AMP@Zn/Mn‐MOF. (F) Zeta potentials of Zn/Mn‐MOF and AMP@Zn/Mn‐MOF. n = 5. (G) XRD patterns of Zn/Mn‐MOF and AMP@Zn/Mn‐MOF. (H) FTIR spectrum of Zn/Mn‐MOF, c‐di‐AMP diammonium, and AMP@Zn/Mn‐MOF. (I) XPS spectra of Zn/Mn‐MOF and AMP@Zn/Mn‐MOF. (J) High‐resolution O 1s XPS spectrum of AMP@Zn/Mn‐MOF. Data are presented as mean ± SD. Statistical significance was calculated by a two‐tailed unpaired Student's t‐test. ***p < 0.001.

Zn/Mn‐MOF was synthesized through self‐assembly via the use of Zn(NO_3_)_2_, Mn(NO_3_)_2_, and 2‐methylimidazole(2‐MIM) in a mixture of methanol [23, 24]. To reduce the cytotoxicity of Zn^2+^/Mn^2+^ and effectively activate cGAS–STING signaling, the STING agonist c‐di‐AMP diammonium was encapsulated in the Zn/Mn‐MOF. The surface of Zn/Mn‐MOF was subsequently modified with polyethylene glycol (PEG) to increase its colloidal stability and biocompatibility (Figure 2A). The morphology and elemental distribution of AMP@Zn/Mn‐MOF were characterized by scanning electron microscopy (SEM) and energy‐dispersive X‐ray spectroscopy (EDS) mapping. AMP@Zn/Mn‐MOF displayed a monodispersed polyhedral morphology (Figure 2B,C, Figure S1), further providing visual evidence for the successful encapsulation of c‐di‐AMP diammonium. Moreover, the atomic force microscopy (AFM) image revealed a hexagonal‐like morphology of AMP@Zn/Mn‐MOF with similar morphologies to those of the particles observed by SEM (Figure 2B–D). The average thickness of AMP@Zn/Mn‐MOF was ∼200 nm, which was in line with the results of the hydrodynamic size measurements (Figure 2D,E, Figure S2). Following the encapsulation of c‐di‐AMP diammonium, the zeta potential value of the synthesized Zn/Mn‐MOF shifted from +26 to +10 mV, which can be attributed to the negative charges carried by c‐di‐AMP diammonium (Figure 2F). Furthermore, X‐ray diffraction (XRD) patterns were obtained to further characterize the effects of the c‐di‐AMP diammonium modification. XRD analysis confirmed that the crystal structure of AMP@Zn/Mn‐MOF remained consistent with that of Zn/Mn‐MOF, indicating that no structural alteration occurred (Figure 2G). In addition, the Fourier transform infrared (FTIR) spectrum of AMP@Zn/Mn‐MOF revealed typical peaks, indicating the successful AMP@Zn/Mn‐MOF construction (Figure 2H). The ultraviolet–visible (UV‐vis) curve results revealed that c‐di‐AMP diammonium exhibited a characteristic absorption peak at 258 nm (Figures S3 and S4). The drug loading content (LC%) of the c‐di‐AMP diammonium was calculated as ∼40.48%. Brunauer−Emmett−Teller (BET) analysis and pore size distribution curve analysis revealed that Zn/Mn‐MOF possessed a porous structure with a surface area of 1249.78 m^2^/g and a Barrett−Joyner−Halenda (BJH) pore diameter of 2.1 nm (Figure S5A–E), thereby effectively increasing the c‐di‐AMP diammonium loading. Moreover, inductively coupled plasma‒mass spectrometry (ICP‒MS) analysis revealed that the actual Mn content [Mn/(Mn+Zn)] for AMP@Zn/Mn‐MOF was 6.1%. In addition, X‐ray photoelectron spectroscopy (XPS) was employed to analyze the surface elemental composition, revealing characteristic signals for Mn, Zn, N, and O in the synthesized AMP@Zn/Mn‐MOF. Mn 2p and Zn 2p peaks were detected in both Zn/Mn‐MOF and AMP@Zn/Mn‐MOF (Figure 2I), confirming the successful modification of Zn/Mn‐MOF with c‐di‐AMP diammonium. Additionally, the intensities of the O 1s and P 2p peaks increased in AMP@Zn/Mn‐MOF (Figure 2J, Figure S6), suggesting effective loading of c‐di‐AMP diammonium onto the Zn/Mn‐MOF surface. These results confirmed successful AMP@Zn/Mn‐MOF fabrication.

### 2.2 Cellular Uptake and Cytotoxicity of AMP@Zn/Mn‐MOF

**FIGURE 3:**
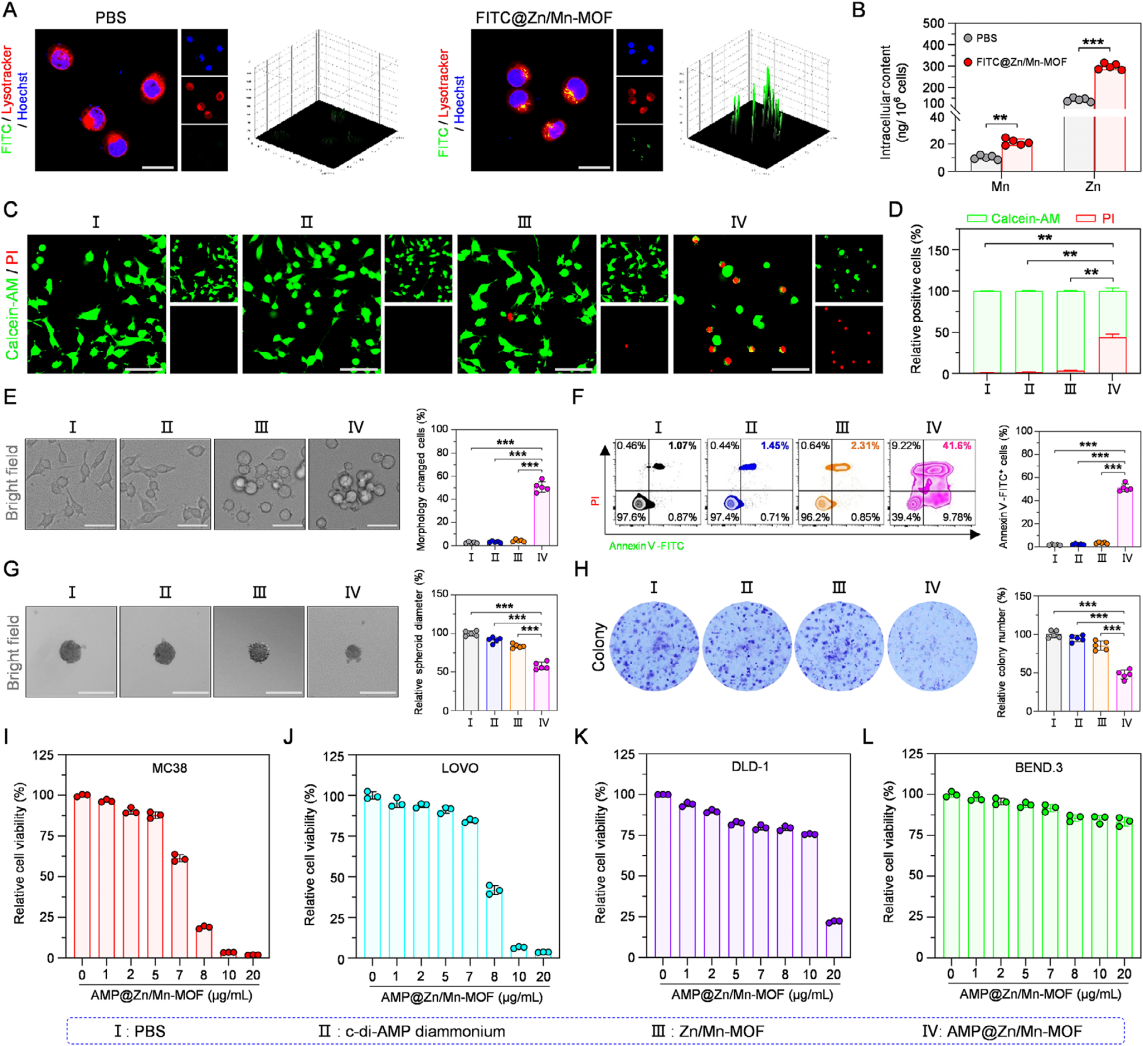
Cellular uptake and antitumor efficacy of AMP@Zn/Mn‐MOF in vitro. (A) CLSM images of MC38 cells treated with PBS and FITC@Zn/Mn‐MOF. Scale bar: 25 µm. (B) The cellular content of Zn and Mn in PBS and FITC@Zn/Mn‐MOF‐treated MC38 cells by ICP‐MS. n = 5. (C,D) CLSM images of MC38 cells stained with Calcein‐AM (green, indicating live cells) and PI (red, indicating dead cells). Scale bar: 100 µm. n = 5. (E) Microscopic images of MC38 cells after various treatments. Scale bar: 40 µm. n = 5. (F) Flow cytometry analysis of MC38 cells stained with Annexin V‐FITC and PI. n = 5. (G) Microscopic images of MC38 tumor spheroids following diverse treatments. Scale bar: 200 µm. n = 5. (H) Representative images of MC38 cell colonies after exposure to various treatments. n = 5. (I–L) Cytotoxic effects on MC38, LOVO, DLD‐1, and BEND.3 cells treated with different concentrations of AMP@Zn/Mn‐MOF. n = 3. Data are presented as mean ± SD. Statistical significance was calculated by a two‐tailed unpaired Student's t‐test. **p < 0.01, ***p < 0.001.

The cellular uptake of Zn/Mn‐MOF, which was labeled with fluorescein isothiocyanate (FITC) dye, was assessed in murine colorectal cancer (MC38) cells via confocal laser scanning microscopy (CLSM). The CLSM images revealed significantly stronger green fluorescence signals within the lysosomes in the FITC@Zn/Mn‐MOF‐treated MC38 cells (Figure 3A). ICP‒MS was utilized to measure the intracellular accumulation of Zn^2+^/Mn^2+^ to further quantify cellular uptake. The results indicated that the Zn^2+^/Mn^2+^ content was significantly greater in the FITC@Zn/Mn‐MOF‐treated MC38 cells than in those treated with phosphate‐buffered saline (PBS) (Figure 3B). Furthermore, these results were corroborated by subsequent flow cytometry analysis (Figure S7). These findings demonstrated that Zn/Mn‐MOF could serve as a promising nanocarrier for effective drug delivery applications.

After cellular phagocytosis of AMP@Zn/Mn‐MOF, its cytotoxicity was evaluated using calcein‐AM and propidium iodide (PI) staining. Notably, a decreased live/dead ratio was observed in the AMP@Zn/Mn‐MOF‐treated group (Figure 3C,D), confirming the cytotoxicity potential of AMP@Zn/Mn‐MOF in MC38 cells. Additionally, the effect of AMP@Zn/Mn‐MOF on cell viability was monitored through morphological alterations. The AMP@Zn/Mn‐MOF‐treated MC38 cells exhibited significant disruption of cell membrane integrity (Figure 3E). Moreover, annexin V‐FITC/PI staining was employed to confirm the antitumor performance of AMP@Zn/Mn‐MOF in vitro. The flow cytometry results revealed a significant increase in the proportion of necrotic cells following treatment with AMP@Zn/Mn‐MOF compared with that in the other treatment groups (Figure 3F). To better understand the antitumor effects of AMP@Zn/Mn‐MOF on solid tumors, a 3D tumor spheroid culture model was established to monitor the cytotoxicity of AMP@Zn/Mn‐MOF. As depicted in Figure 3G, compared with that in the other groups, the growth of 3D MC38 spheroids in the AMP@Zn/Mn‐MOF‐treated group was notably suppressed. In addition, a long‐term colony formation assay was employed to further evaluate the effect of AMP@Zn/Mn‐MOF treatment on MC38 cell proliferation. As depicted in Figure 3H, the AMP@Zn/Mn‐MOF group exhibited fewer colonies with smaller sizes, suggesting that AMP@Zn/Mn‐MOF effectively inhibited colony formation. We subsequently investigated the superior cytotoxic efficacy of AMP@Zn/Mn‐MOF against multiple colorectal cancer cell lines, including murine MC38 cells and the human colorectal cancer cell lines LOVO and DLD‐1. Interestingly, a cell counting kit‐8 (CCK‐8) assay revealed that compared with either c‐di‐AMP diammonium or Zn/Mn‐MOF alone, AMP@Zn/Mn‐MOF exhibited greater cytotoxicity against MC38 cells (Figure 3I, Figures S8 and S9), suggesting that the combination of c‐di‐AMP diammonium and Zn/Mn‐MOF significantly enhanced the therapeutic effectiveness in promoting MC38 cell death. Furthermore, AMP@Zn/Mn‐MOF dose‐dependently inhibited LOVO and DLD‐1 cells (Figure 3J,K), indicating the broad‐spectrum cytotoxicity and potent anticancer activity of AMP@Zn/Mn‐MOF. However, at the same high concentration of AMP@Zn/Mn‐MOF, lower cytotoxicity was observed in nontumor cell lines (BEND.3, murine brain‐derived endothelial cells) than in tumor cells (Figure 3L), likely due to the higher metabolic activity rate of tumor cells. These data demonstrated that AMP@Zn/Mn‐MOF has potential applications in cancer therapy.

### 2.3 pH‐Responsive Release Profile of AMP@Zn/Mn‐MOF

**FIGURE 4:**
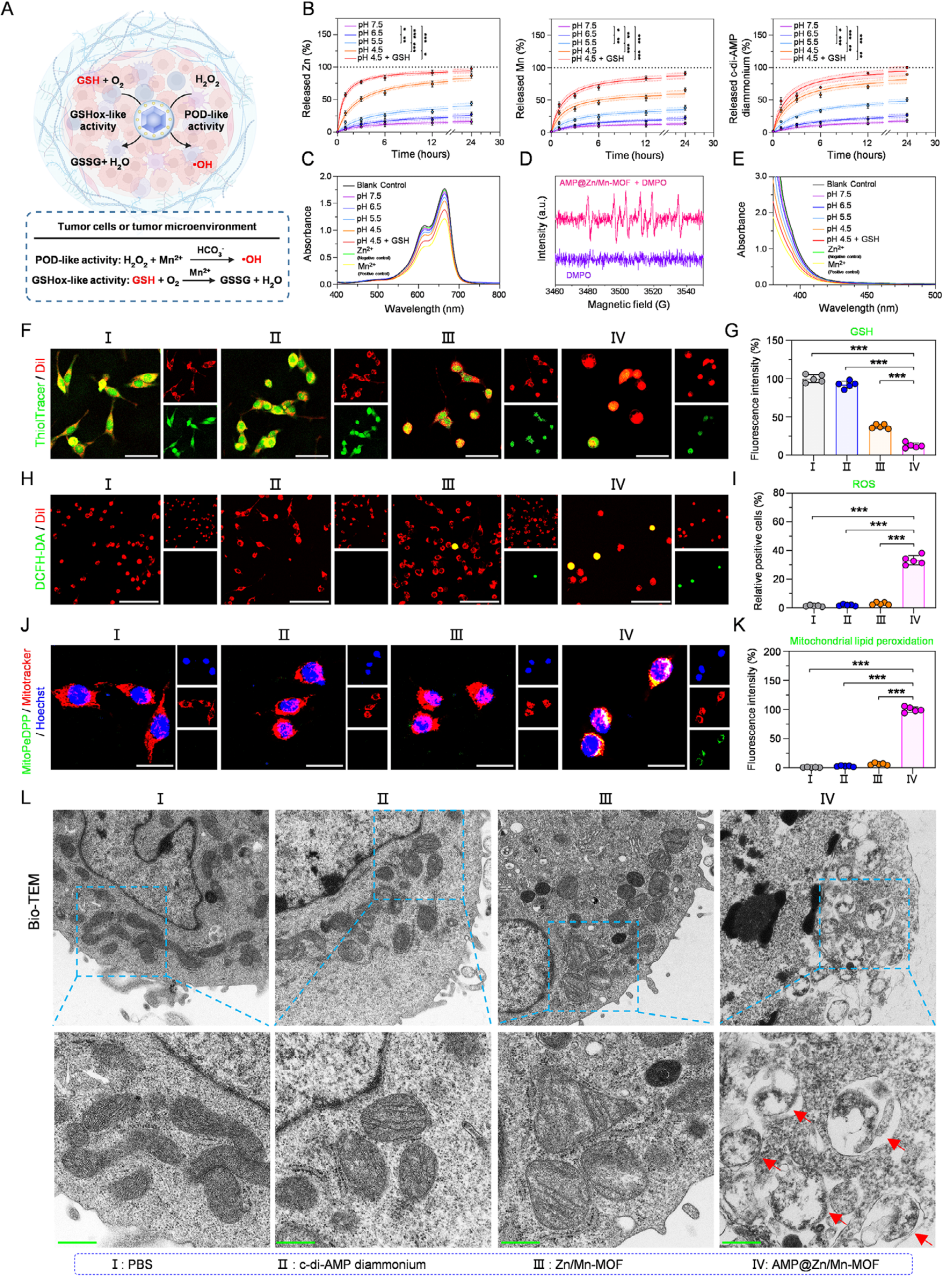
pH‐responsive release profile and catalytic activities of AMP@Zn/Mn‐MOF. (A) Schematic illustration of pH‐responsive release profile and catalytic activities of AMP@Zn/Mn‐MOF. Created in BioRender. Zhu, B. (2026) https://BioRender.com/0ze9fqb. (B) Cumulative release profiles of Zn^2+^, Mn^2+^, and c‐di‐AMP diammonium from AMP@Zn/Mn‐MOF after stirring in buffer solutions at varying pH levels. n = 3. (C) UV−vis absorption spectra of •OH‐induced MB degradation by AMP@Zn/Mn‐MOF with different H_2_O_2_ and GSH concentrations. (D) EPR spectra of •OH produced by AMP@Zn/Mn‐MOF. (E) UV−vis absorption spectra of DTNB following incubation with AMP@Zn/Mn‐MOF in the presence of various concentrations of H_2_O_2_ and GSH. (F, G) CLSM images of MC38 cells stained with ThiolTracker (a GSH detection probe) after different treatments. Scale bar: 50 µm. n = 5. (H,I) CLSM images of MC38 cells stained with DCFH‐DA (a ROS detection probe) after different treatments. Scale bar: 100 µm. n = 5. (J,K) CLSM images of MC38 cells stained with MitoPeDPP (a mitochondrial ROS probe) after different treatments. Scale bar: 20 µm. n = 5. (L) Bio‐TEM images of mitochondria in MC38 cells following various treatments. Scale bar: 400 nm. Data are presented as mean ± SD. Statistical significance was calculated by a two‐tailed unpaired Student's *t*‐test. *p < 0.05, **p < 0.01, ***p < 0.001.

To investigate release dynamics, we evaluated the release behaviors of c‐di‐AMP diammonium, Zn ions, and Mn ions from the nanoplatform in an acidic aqueous environment (Figure 4A). When stirred in a sodium acetate buffer solution (pH 4.5), approximately 89.4% of c‐di‐AMP diammonium was released from AMP@Zn/Mn‐MOF over 24 h. In contrast, at pH values of 5.5 and 6.5, the release percentages decreased to 50.6% and 27.1%, respectively (Figure 4B). Therefore, Mn and Zn ions were released in a pH‐dependent manner. ICP‒MS analysis revealed that after 24 h of incubation in a sodium acetate buffer solution (pH 4.5), AMP@Zn/Mn‐MOF released 65.7% of the Mn ions and 86.8% of the Zn ions. In a neutral buffer solution (pH 7.5), only 14.5% of the Mn ions and 16.2% of the Zn ions were released. Moreover, under conditions of 10 mM glutathione (GSH) and a pH of 4.5, the release of Mn and Zn ions from AMP@Zn/Mn‐MOF exceeded 91.3% and 97.6%, respectively, within 24 h (Figure 4B). Additionally, 99.8% of c‐di‐AMP diammonium was released from AMP@Zn/Mn‐MOF within the same period at pH 4.5 and 10 mM GSH (Figure 4B). These findings suggested that the pH/GSH dual responsiveness of AMP@Zn/Mn‐MOF might contribute to its potential in tumor treatment.

### 2.4 Catalytic Activities of AMP@Zn/Mn‐MOF

Given the ability of Mn^2+^ to catalyze Fenton‐like reactions [25], the POD‐mimetic catalytic activity of AMP@Zn/Mn‐MOF was assessed using H_2_O_2_ as the substrate and methylene blue (MB) as the indicator to examine the production of the hydroxyl radical (•OH) [26]. As shown in Figure 4C, under neutral conditions (pH 7.5), a small amount of •OH was detected for AMP@Zn/Mn‐MOF. However, under acidic conditions (pH 4.5), a gradual increase in the •OH was observed. The addition of GSH further enhanced •OH production, indicating a pH/GSH dual response that synergistically increased the efficiency of the Fenton‐like reaction (Figure 4C, Figure S10). Electron paramagnetic resonance (EPR) with 5,5‐dimethyl‐1‐pyrroline N‐oxide (DMPO) as a spin‐trapping agent confirmed the strongest •OH signal in the AMP@Zn/Mn‐MOF group (Figure 4D). Additionally, the high levels of GSH in tumor tissues could consume ROS during the catalytic process [27]. To confirm the acid‐ and GSH‐activated decomposition behavior of the nanoplatforms, 5,5′‐dithio‐bis(2‐nitrobenzoic acid) (DTNB) was used as a probe to measure the GSH consumption capability of AMP@Zn/Mn‐MOF. UV‐vis spectral analysis revealed that the DTNB absorption peak at approximately 410 nm decreased significantly with increasing acidification (Figure 4E, Figure S10), indicating the high pH dependence of the GSH depletion ability of AMP@Zn/Mn‐MOF. These results suggested that AMP@Zn/Mn‐MOF catalytically converted endogenous H_2_O_2_ to generate an ROS storm for tumor treatment.

We further investigated the GSH depletion capacity of AMP@Zn/Mn‐MOF in tumor cells, and the intracellular GSH level was determined by staining with a ThiolTracker probe [28]. When MC38 cells were treated with AMP@Zn/Mn‐MOF, their GSH levels were distinctly reduced (Figure 4F,G, Figure S11), which was consistent with the experimental results in solution (Figure 4E, Figure S10). To investigate the oxidative damage capacity of AMP@Zn/Mn‐MOF in tumor cells, 2′,7′‐dichlorodihydrofluorescein diacetate (DCFH‐DA) was employed as a fluorescent probe to measure the intracellular ROS level [29, 30]. The CLSM images revealed that the intracellular green fluorescence intensity was highest in the AMP@Zn/Mn‐MOF group (Figure 4H,I), indicating that AMP@Zn/Mn‐MOF could efficiently produce ROS and trigger intracellular oxidative stress. Moreover, the accumulation of ROS in MC38 cells led to severe lipid peroxidation and mitochondrial depolarization. MitoPeDPP was used to identify the production of ROS in the mitochondria [31]. The AMP@Zn/Mn‐MOF group exhibited the strongest fluorescence intensity (Figure 4J,K), confirming that AMP@Zn/Mn‐MOF could induce obvious oxidative damage. Additionally, specific mitochondrial morphological changes were examined using bio‐TEM [32]. Compared with the normal morphology of mitochondria, the typical characteristics of mitochondrial dysfunction after AMP@Zn/Mn‐MOF treatment were observed (Figure 4L). Given the above results, the concurrent ROS generation and GSH depletion capacities of AMP@Zn/Mn‐MOF could induce tumor cell death.

### 2.5 AMP@Zn/Mn‐MOF Stimulates cGAS–STING Pathway Activation

**FIGURE 5:**
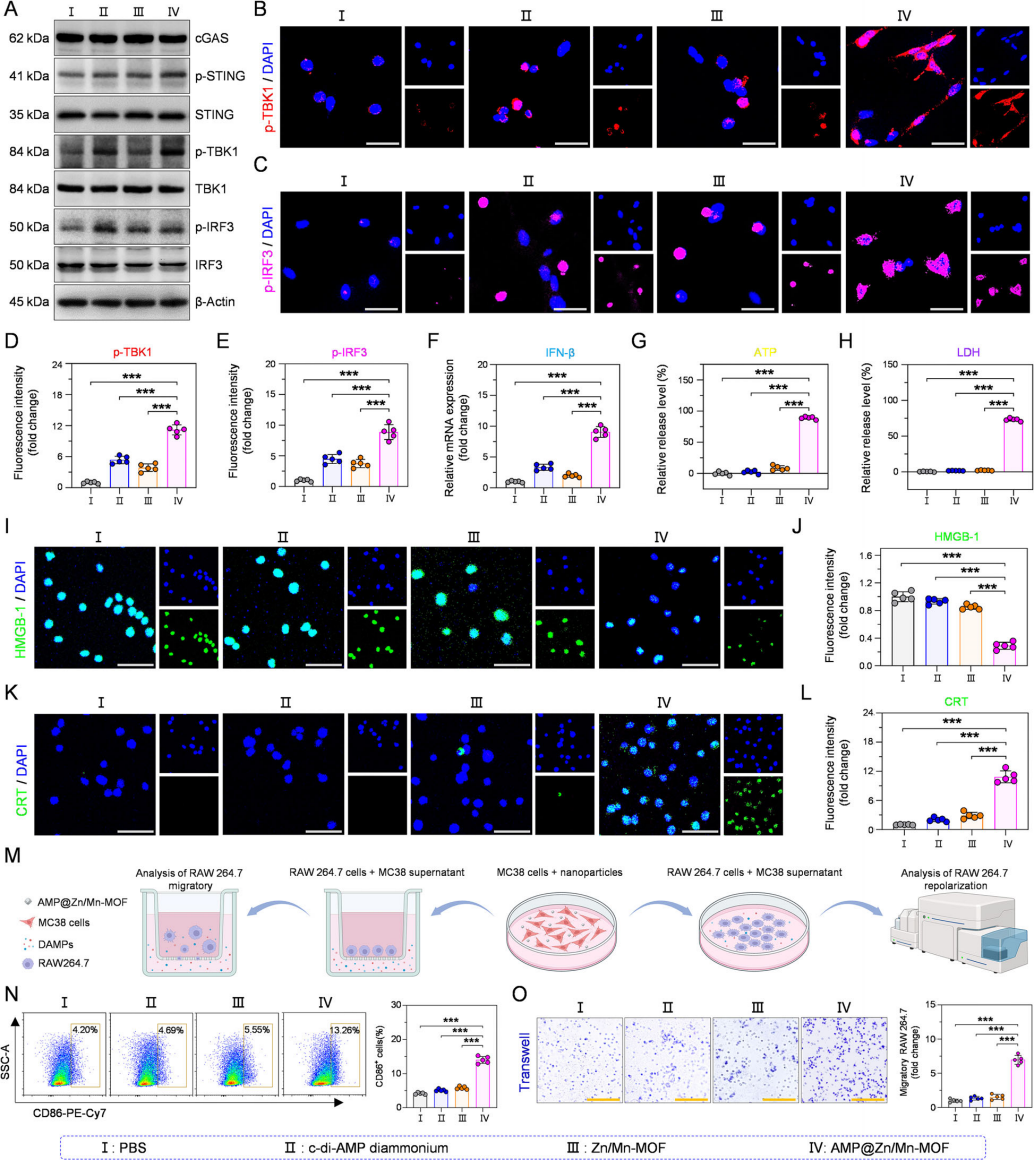
Synergistic activation of cGAS‐STING pathway and ICD by AMP@Zn/Mn‐MOF. (A) Western blot analysis of STING pathway activation in MC38 cells following the indicated treatments. (B,C) CLSM images of p‐TBK1 and p‐IRF3 in MC38 cells subjected to different treatments. Scale bar: 30 µm. (D,E) Quantitative analysis of the relative fluorescence intensity of p‐TBK1 and p‐IRF3 in MC38 cells subjected to different treatments. n = 5. (F) RT‐qPCR analysis of IFN‐β mRNA expression in MC38 cells subjected to different treatments. n = 5. (G,H) Levels of ATP and LDH released from MC38 cells subjected to different treatments. n = 5. (I,J) CLSM images and relative fluorescence intensity of HMGB‐1 expression in MC38 cells following the indicated treatments. Scale bar: 50 µm. n = 5. (K,L) CLSM images and relative fluorescence intensity of CRT expression in MC38 cells following the indicated treatments. Scale bar: 50 µm. n = 5. (M) Schematic illustration of AMP@Zn/Mn‐MOF‐induced ICD‐activated immune response of RAW264.7 cells. Created in BioRender. Zhu, B. (2026) https://BioRender.com/a5h6o2i. (N) Flow cytometric and statistical analysis of the matured RAW264.7 cells after incubation with different medium. n = 5. (O) Chemotaxis and statistical analysis of the migrated RAW264.7 cells after indicated treatments. n = 5. Scale bar: 150 µm. Data are presented as mean ± SD. Statistical significance was calculated by a two‐tailed unpaired Student's t‐test. ***p < 0.001.

As demonstrated in recent research, Zn^2+^, Mn^2+^, or c‐di‐AMP diammonium can effectively activate the cGAS–STING pathway [17, 33]. The activation of the cGAS–STING pathway in tumor cells induced by AMP@Zn/Mn‐MOF was explored using western blotting. MC38 cells treated with AMP@Zn/Mn‐MOF presented the highest levels of phosphorylated STING (p‐STING), phosphorylated TBK1 (p‐TBK1), and phosphorylated IRF3 (p‐IRF3) (Figure 5A, Figure S12), indicating robust activation of the cGAS–STING pathway. In contrast, the expression level of cGAS, which senses cytosolic dsDNA fragments for STING activation [34], remained almost unchanged (Figure 5A, Figure S12). Moreover, CLSM analysis revealed that the fluorescence intensity of p‐TBK1 and p‐IRF3 was markedly stronger after AMP@Zn/Mn‐MOF treatment than after c‐di‐AMP diammonium or Zn/Mn‐MOF treatment (Figure 5B–E). Furthermore, IFN‐β, a key effector involved in the activation of the cGAS–STING pathway [35], was detected in MC38 cells after the indicated treatments. Compared with that in the other groups, the mRNA expression level of IFN‐β in the AMP@Zn/Mn‐MOF group was notably elevated (Figure 5F). Previous studies have shown that STING also triggers proinflammatory responses through the NF‐κB pathway in macrophages [36]. Notably, the expression of phosphorylated NF‐κB (p‐NF‐κB) was not affected in the AMP@Zn/Mn‐MOF‐treated MC38 cells compared with the control cells (Figure S13). Next, to exclude the activation of the cGAS–STING pathway induced by cyclic GMP‐AMP (cGAMP) [9], an enzyme‐linked immunosorbent assay (ELISA) was performed to analyze the level of endogenous cGAMP triggered by the nanoparticles. Compared with those in the control groups, the intracellular cGAMP levels in the AMP@Zn/Mn‐MOF‐treated MC38 cells remained almost unchanged (Figure S14). These results demonstrated that AMP@Zn/Mn‐MOF effectively activated the cGAS–STING pathway and stimulated immune responses.

### 2.6 AMP@Zn/Mn‐MOF Induces ICD

In our design, AMP@Zn/Mn‐MOF efficiently induced cell death and activated the cGAS–STING signaling pathway, thereby triggering ICD. During ICD, rupture of the cell membrane resulted in the release of adenosine triphosphate (ATP), high‐mobility group box‐1 protein (HMGB‐1), and lactate dehydrogenase (LDH), leading to the translocation of calreticulin (CRT) to the cell membrane surface [37, 38]. Compared with those in the control groups, the intracellular ATP levels in the AMP@Zn/Mn‐MOF treatment group were significantly lower (Figure 5G). Similarly, the level of LDH released was greater in the AMP@Zn/Mn‐MOF group than in the other groups (Figure 5H), indicating that AMP@Zn/Mn‐MOF significantly disrupted the integrity of the cell membrane. Accordingly, the fluorescence intensity of HMGB‐1 in the cell nucleus was significantly reduced following treatment with AMP@Zn/Mn‐MOF (Figure 5I,J). Compared with the PBS group, the AMP@Zn/Mn‐MOF group showed a marked increase in CRT fluorescence intensity (Figure 5K,L), which further supported the occurrence of ICD. To further confirm the immunogenic nature of AMP@Zn/Mn‐MOF‐induced ICD, the immune responses of mouse macrophages (RAW264.7) were investigated (Figure 5M). Flow cytometric analysis of RAW264.7 cells after different treatments revealed that the proportion of CD86^+^ macrophages increased gradually after the supernatants of AMP@Zn/Mn‐MOF‐treated MC38 cells were incubated (Figure 5N, Figure S15). We next investigated this trend in bone marrow‐derived macrophages (BMDMs) incubated with AMP@Zn/Mn‐MOF *i* *n vitro*. Consistently, AMP@Zn/Mn‐MOF treatment increased the expression of CD86 in BMDMs, indicating that the activation of ICD promoted the polarization of macrophages for immune activation (Figure S16). Like macrophages, compared with the control treatment, AMP@Zn/Mn‐MOF treatment significantly increased the maturation ability of DC2.4 cells (Figure S17). Furthermore, exposure to the supernatants of MC38 cells treated with AMP@Zn/Mn‐MOF substantially enhanced macrophage migration (Figure 5O), which verified that AMP@Zn/Mn‐MOF dramatically improved the immunogenicity of MC38 cells. These results indicated that the combined activation of ICD and the cGAS–STING pathway by AMP@Zn/Mn‐MOF could effectively enhance immune responses.

### 2.7 Antitumor Effect of AMP@Zn/Mn‐MOF

**FIGURE 6:**
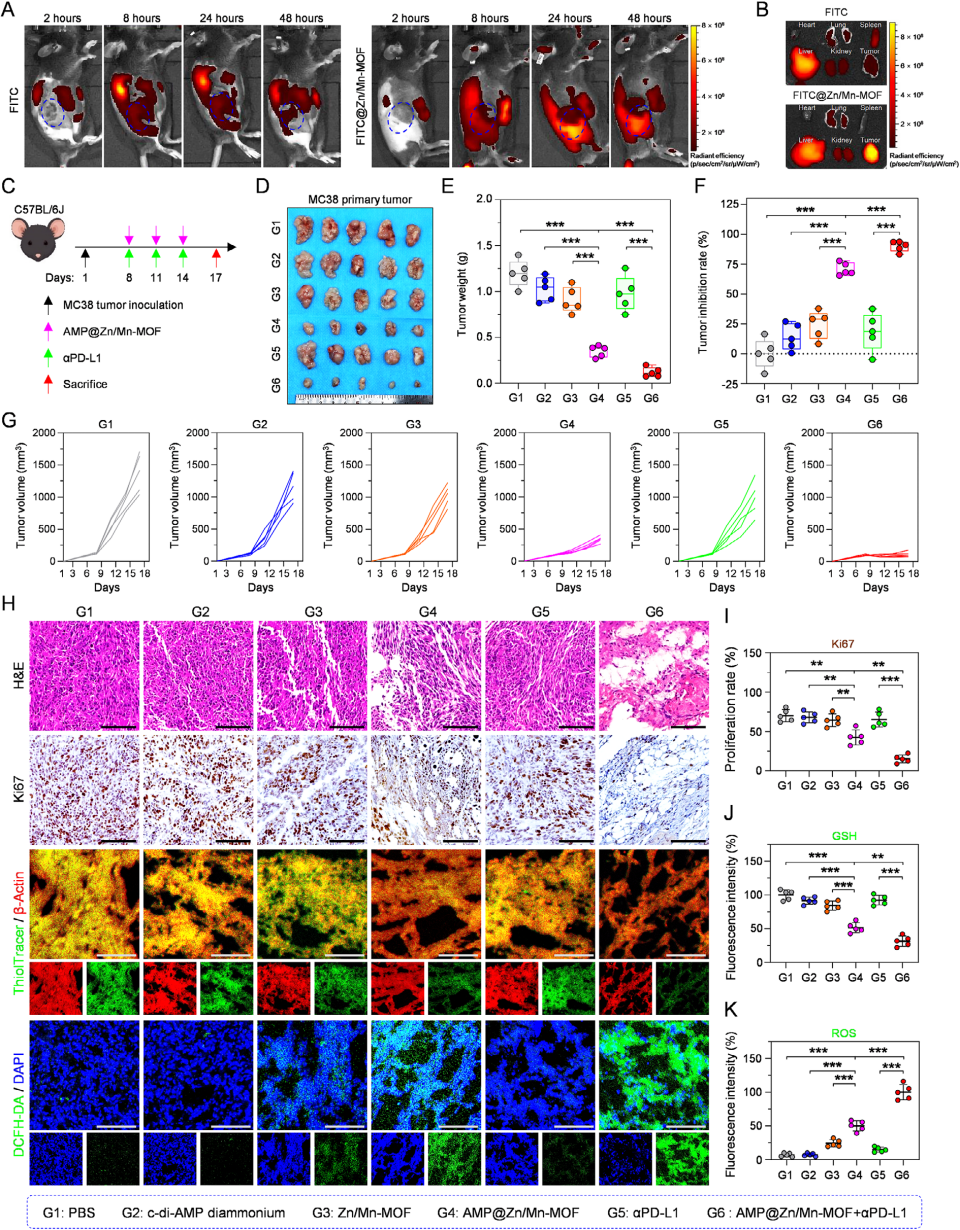
In vivo antitumor effect of AMP@Zn/Mn‐MOF combined with αPD‐L1. (A) In vivo fluorescence imaging of MC38 tumor‐bearing mice at various time points following intravenous injection of free FITC or FITC@Zn/Mn‐MOF. (B) Fluorescence images of major organs (heart, liver, spleen, lung, and kidney) and tumors excised from MC38 tumor‐bearing mice 48 h post‐intravenous administration. (C) Schematic illustration of the in vivo antitumor experimental protocol. Created in Figdraw. (D) Digital images of primary tumors resected from each mouse following different treatments. (E) Profile of tumor weight in mice subjected to different treatments. n = 5. (F) Tumor inhibition rate in mice following different treatments. n = 5. (G) Tumor growth curves for each mouse in each group after different treatments. n = 5. (H) Histological analysis (H&E, Ki67, GSH, and ROS staining) of tumor tissues in mice following different treatments. Scale bar: 100 µm. (I–K) Quantitative analysis of Ki67 index and the relative fluorescence intensity of GSH and ROS in tumor tissues following different treatments. n = 5. Data are presented as mean ± SD. Statistical significance was calculated by a two‐tailed unpaired Student's t‐test. **p < 0.01, ***p < 0.001.

On the basis of the excellent activation of the cGAS–STING pathway and cancer cell‐killing ability of AMP@Zn/Mn‐MOF, its therapeutic effect was evaluated in mice using an aggressive and poorly immunogenic MC38 tumor model. The biodistribution of the nanoparticles was systematically assessed at various time points following intravenous injection of FITC‐labeled Zn/Mn‐MOF (FITC@Zn/Mn‐MOF) into MC38 tumor‐bearing mice. In vivo imaging system (IVIS) results revealed substantial accumulation of FITC@Zn/Mn‐MOF at tumor sites at 24 h after injection, confirming its effective tumor‐targeting ability (Figure 6A, Figure S18A). *Ex vivo* biodistribution images demonstrated that FITC@Zn/Mn‐MOF primarily accumulated in tumor tissues, with minimal fluorescence signals detected in the lungs and liver (Figure 6B, Figure S18B). Immunofluorescence staining of tumor sections and ICP‒MS analysis of tumors excised from mice treated with FITC@Zn/Mn‐MOF further corroborated its superior tumor‐targeting ability (Figure S18C–E).

As previously demonstrated, cGAS–STING signaling upregulates PD‐L1 expression in certain contexts [39]. Moreover, an αPD‐L1 antibody enhances antitumor immune responses by inhibiting the interaction between PD‐L1 on tumor cells and PD‐1 on CTLs [40]. We subsequently explored whether AMP@Zn/Mn‐MOF could modulate PD‐L1 expression. Western blot analysis revealed that AMP@Zn/Mn‐MOF did not influence PD‐L1 expression in MC38 cells (Figure S19), highlighting tumor heterogeneity. To evaluate the antitumor effects of AMP@Zn/Mn‐MOF, we established an MC38 tumor‐bearing mouse model by injecting MC38 cells subcutaneously into the right flanks of male C57BL/6J mice. When the tumor volume reached approximately 100 mm^3^, the mice were randomly divided into six groups (n = 5 per group): (G1) PBS, (G2) c‐di‐AMP diammonium, (G3) Zn/Mn‐MOF, (G4) AMP@Zn/Mn‐MOF, (G5) αPD‐L1, and (G6) AMP@Zn/Mn‐MOF+αPD‐L1. On day 8 after MC38 tumor inoculation, the mice received an intravenous injection of AMP@Zn/Mn‐MOF and an intraperitoneal injection of αPD‐L1 antibody. Repeated treatments were performed on days 11 and 14 to ensure treatment effectiveness. The weight of the mice and the tumor volume were monitored every 3 days (Figure 6C). Compared with those in the PBS group, tumor growth in the c‐di‐AMP diammonium and αPD‐L1 monotherapy groups was not significantly inhibited (Figure 6D–G, Figure S20), confirming that MC38 tumors were not sensitive to the immunoadjuvant alone. The mild tumor inhibition in the AMP@Zn/Mn‐MOF group was attributed to its cytotoxic effect (Figure 6D–G, Figure S20). More notably, tumor growth was significantly inhibited in the AMP@Zn/Mn‐MOF+αPD‐L1 combination therapy group (Figure 6G, Figure S20), indicating that AMP@Zn/Mn‐MOF‐induced ICD enhanced the therapeutic activity of the αPD‐L1 antibody. At the end of the treatment, the tumors were excised and weighed to verify the tumor‐inhibitory effect. The weight and photographic images of the tumors correlated with the changes in tumor volume (Figure 6D–G, Figure S20). In addition, the combination therapy of AMP@Zn/Mn‐MOF and αPD‐L1 achieved a tumor inhibition rate greater than 89%, surpassing the rates of AMP@Zn/Mn‐MOF (70%), c‐di‐AMP diammonium (14%), or αPD‐L1 (18%) alone (Figure 6F).

Subsequently, tumor tissues from the various subgroups were prepared for histological analysis, including Ki67 immunohistochemical (IHC) staining and hematoxylin and eosin (H&E) staining. Compared with the other groups, the AMP@Zn/Mn‐MOF and αPD‐L1 combination therapy group exhibited obvious tissue damage, nuclear fragmentation, and nucleolysis (Figure 6H). Accordingly, the percentage of Ki67‐positive cells clearly decreased in the AMP@Zn/Mn‐MOF+αPD‐L1 group (Figure 6H,I), further suggesting that the combination therapy had the strongest ability to suppress tumor proliferation. Next, we explored whether the catalytic activities of AMP@Zn/Mn‐MOF occurred in tumor tissues to enhance the antitumor effectiveness of the combination therapy. The CLSM results revealed that the AMP@Zn/Mn‐MOF+αPD‐L1‐treated group presented the highest ROS level and lowest GSH level, while moderate levels of ROS and GSH were observed in the AMP@Zn/Mn‐MOF group (Figure 6H,J,K), indicating the activation of ICD. In conclusion, these results showed that the activation of ICD by AMP@Zn/Mn‐MOF could effectively increase the antitumor effectiveness of the αPD‐L1 antibody in vivo.

### 2.8 RNA‐seq Analysis of the Antitumor Effect

**FIGURE 7:**
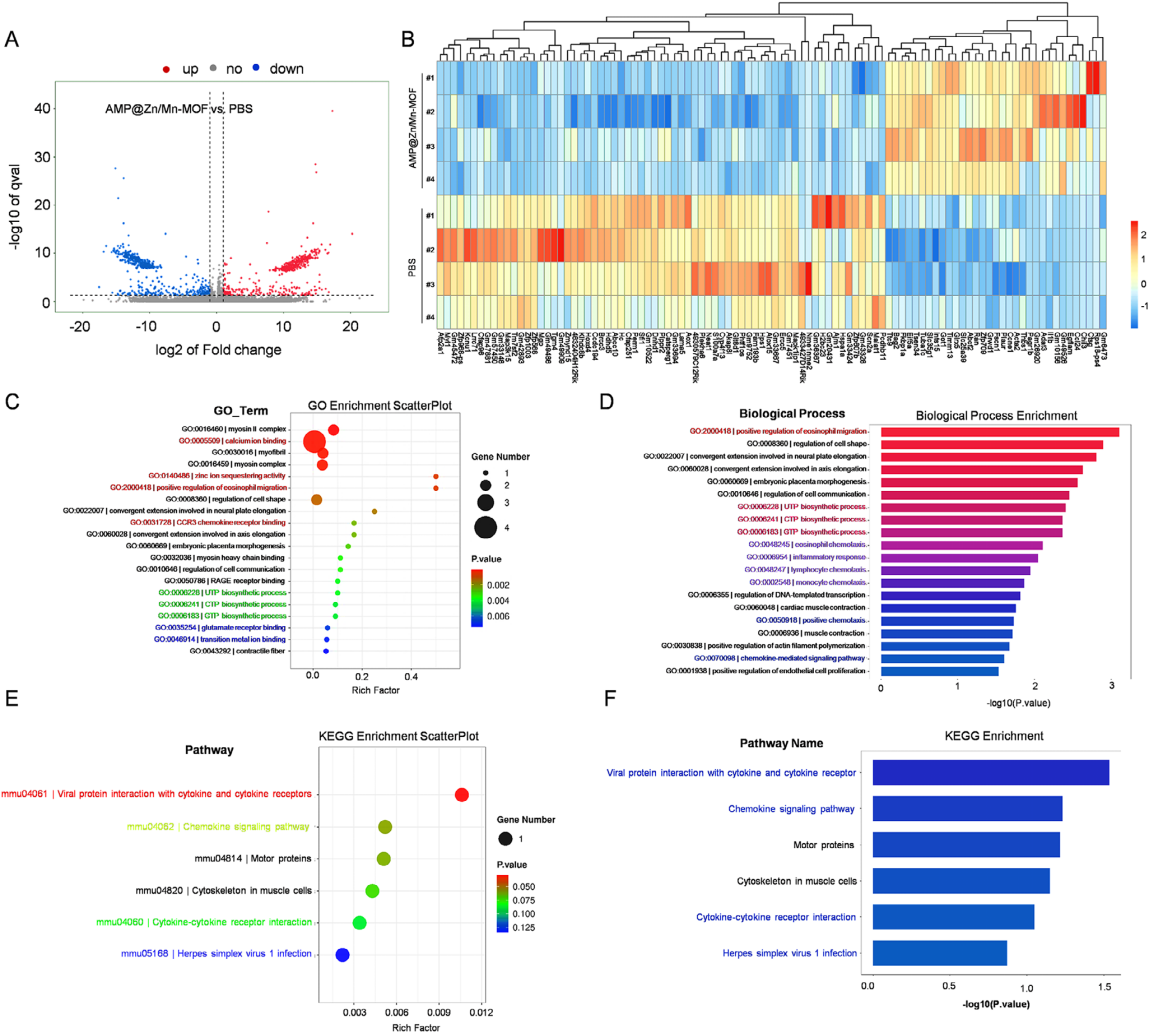
Antitumor immune response evoked by AMP@Zn/Mn‐MOF. (A) Volcano plots depicting significantly upregulated and downregulated genes in the PBS and AMP@Zn/Mn‐MOF groups (log2(Fold Change) ≥ 1.00; *p* ≤ 0.05). n = 4. (B) Heatmap illustrating differential gene expression between the PBS and AMP@Zn/Mn‐MOF groups (log2(Fold Change) ≥1.00; *p* ≤ 0.05). n = 4. (C,D) Gene Ontology (GO) enrichment analysis of differentially expressed pathways comparing the PBS group with the AMP@Zn/Mn‐MOF group (log2(Fold Change) ≥1.00; FDR ≤ 0.01). n = 4. (E,F) Kyoto Encyclopedia of Genes and Genomes (KEGG) pathway analysis of differentially expressed genes between the PBS group and the AMP@Zn/Mn‐MOF group (log2(Fold Change) ≥1.00; FDR ≤ 0.01) based on RNA sequencing data. n = 4.

RNA sequencing (RNA‐seq) of MC38 tumor tissues was conducted to explore the underlying therapeutic mechanisms. Volcano plots revealed 998 genes whose expression differed between the PBS group and the AMP@Zn/Mn‐MOF group, including 488 upregulated genes and 510 downregulated genes (Figure 7A,B). Gene Ontology (GO) enrichment analysis indicated the activation of multiple biological processes, including “calcium ion binding”, “zinc ion sequestering activity”, “positive regulation of eosinophil migration”, and “CCR3 chemokine receptor binding” (Figure 7C,D, Figure S21), indicating that AMP@Zn/Mn‐MOF contributed to antitumor immunity. To further elucidate the mechanisms underlying cGAS–STING activation, Kyoto Encyclopedia of Genes and Genomes (KEGG) enrichment analysis revealed that AMP@Zn/Mn‐MOF‐treated tumors activated several key pathways, such as “viral protein interaction with cytokine and cytokine receptors”, “chemokine signaling pathway”, “cytokine–cytokine receptor interaction”, and “herpes simplex virus 1 infection” (Figure 7E,F, Figure S22). As indicated, the cGAS–STING pathway is key in modulating antiviral and inflammatory immune responses [11]. In line with the findings of previous studies, activating the “chemokine signaling pathway” and “herpes simplex virus 1 infection” further confirmed the activation of the cGAS–STING pathway in AMP@Zn/Mn‐MOF‐treated tumors (Figure 7E,F, Figure S22). Additionally, several genes associated with the cell cycle, including cyclin D3 (Ccnd3), CDC14 cell division cycle 14A (Cdc14a), cell cycle progression 1 (Ccpg1), RB transcriptional corepressor 1 (Rb1), and RB transcriptional corepressor like 2 (Rbl2), were significantly downregulated in tumors treated with AMP@Zn/Mn‐MOF (Figure 7B), which further demonstrated that AMP@Zn/Mn‐MOF treatment could effectively suppress the proliferation of tumor cells.

Proinflammatory cytokines play crucial roles in activating, mobilizing, and enhancing the activity of immune cells, thereby bolstering the immune response against tumors [41]. To assess the impact of AMP@Zn/Mn‐MOF on this process, ELISA was used to measure the levels of key proinflammatory cytokines linked to cGAS–STING pathway activation in vivo. Treatment with AMP@Zn/Mn‐MOF led to a marked increase in the serum concentrations of TNF‐α, IL‐6, and IFN‐β, which were significantly greater than those observed in the PBS‐treated group (Figure S23). Additionally, the mRNA expression levels of several genes associated with enhanced immune responses [42, 43], such as Ccl24 (C–C motif chemokine ligand 24), Il1b (interleukin 1 beta), Irak2 (interleukin‐1 receptor‐associated kinase 2), Il11ra1 (interleukin 11 receptor subunit alpha 1), Tnfaip2 (tumor necrosis factor, alpha‐induced protein 2), Traf3 (TNF receptor‐associated factor 3), and Ly6e (lymphocyte antigen 6 family member E) (Figure 7B), were notably elevated in tumors treated with AMP@Zn/Mn‐MOF. Collectively, these findings confirmed that AMP@Zn/Mn‐MOF effectively activated the cGAS‒STING pathway, thereby eliciting a potent systemic antitumor immune response.

### 2.9 AMP@Zn/Mn‐MOF NPs and αPD‐L1 Combined Immunotherapy Remodeled the Immunosuppressive TME

**FIGURE 8:**
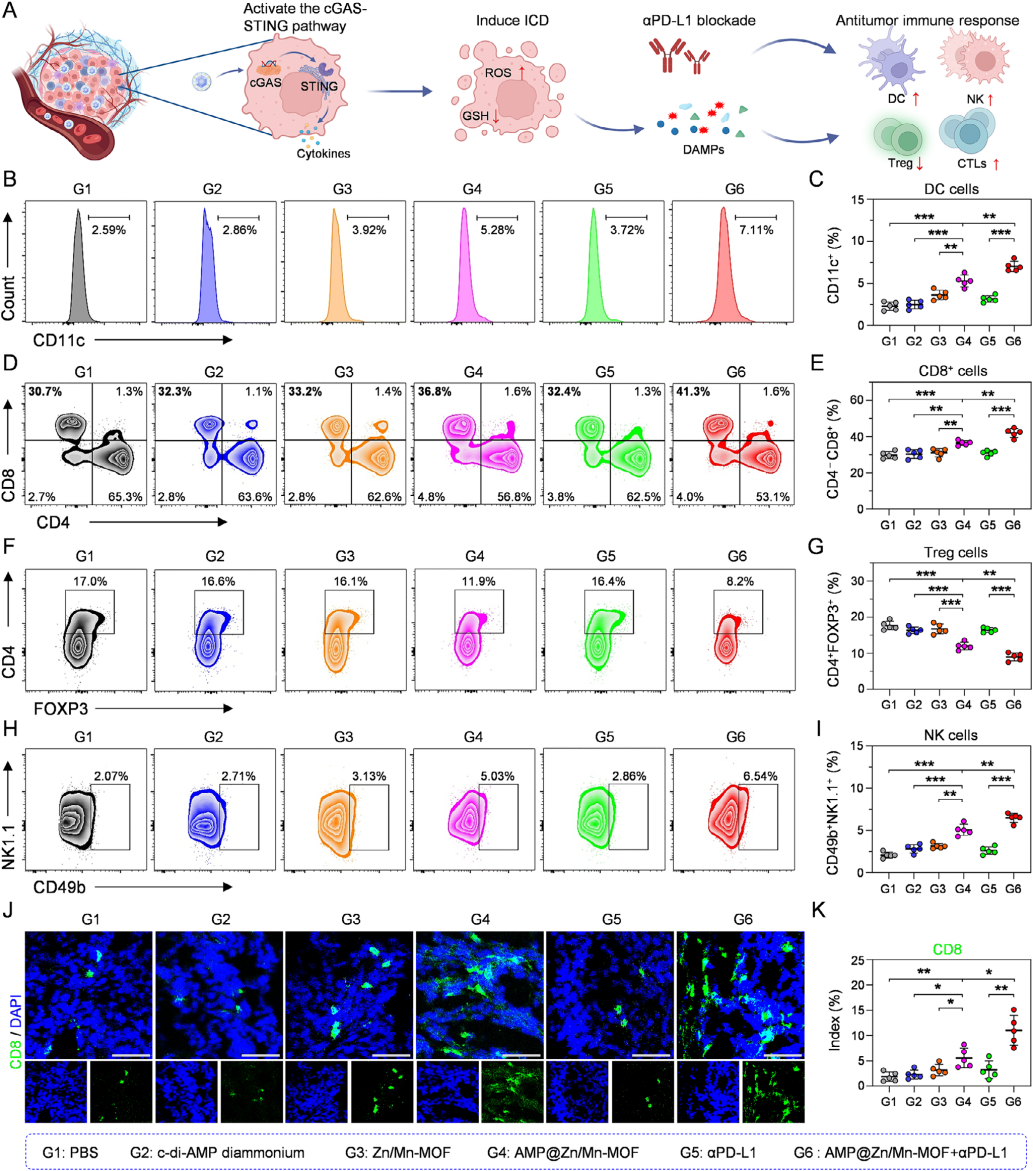
AMP@Zn/Mn‐MOF combined with αPD‐L1 reprogramed the immunosuppressive TME. (A) Schematic illustration of the mechanism underlying the combination of AMP@Zn/Mn‐MOF with αPD‐L1 for antitumor immunotherapy. Created in BioRender. Zhu, B. (2026) https://BioRender.com/layyt5o. (B,C) Flow cytometry analysis and relative quantification of tumor‐infiltrating dendritic cells (CD3^−^CD11c^+^) in mice following the indicated treatments. n = 5. (D,E) Flow cytometry analysis and relative quantification of tumor‐infiltrating CD8^+^ T cells in mice following the indicated treatments. n = 5. (F,G) Flow cytometry analysis and relative quantification of tumor‐infiltrating regulatory T cells (CD4^+^FOXP3^+^) in mice following the indicated treatments. n = 5. (H, I) Flow cytometry analysis and relative quantification of tumor‐infiltrating NK cells (CD49b^+^NK1.1^+^) in mice following the indicated treatments. n = 5. (J,K) Immunofluorescence staining analysis and relative quantification of infiltrating CD8^+^ T cells in tumor tissues after different treatments. n = 5. Scale bar: 100 µm. Data are presented as mean ± SD. Statistical significance was calculated by a two‐tailed unpaired Student's t‐test. *p < 0.05, **p < 0.01, ***p < 0.001.

The combination of AMP@Zn/Mn‐MOF and αPD‐L1 was confirmed to increase antitumor immune effectiveness. The evaluation of tumor‐infiltrating lymphocytes was subsequently conducted using flow cytometry. Fresh tumor tissues were collected from MC38 tumor‐bearing mice at the end of the treatment period (Figure S24). In the AMP@Zn/Mn‐MOF+αPD‐L1 group, the proportion of mature dendritic cells (DCs) in the TME increased significantly from 2.59% to 7.11% (Figure 8A–C), indicating that the activation of the ICD and cGAS–STING pathways promoted DC infiltration and enhanced antigen presentation and immune system activation, which could facilitate subsequent antigen presentation to T cells to prime the adaptive immune response [44]. Tumor‐infiltrating CTLs are crucial for antitumor immunity [45]. After treatment with AMP@Zn/Mn‐MOF+αPD‐L1, the percentage of CD8^+^ CTLs reached 41.3%, which was significantly greater than that in the control group (30.7%). In the absence of αPD‐L1, AMP@Zn/Mn‐MOF alone only modestly increased CTL infiltration (36.8%) (Figure 8D,E). Additionally, the infiltration of immunosuppressive regulatory T cells (Tregs) was reduced from 17.0% to 8.2% in the AMP@Zn/Mn‐MOF+αPD‐L1 group (Figure 8F,G), likely due to the combined effects of ICD and cGAS–STING pathway activation. The proportion of natural killer (NK) cells, another type of cytotoxic lymphocyte [46], was also significantly elevated in the AMP@Zn/Mn‐MOF+αPD‐L1 group (Figure 8H,I). Immunofluorescence staining of tumor tissues revealed a substantially greater number of CD8^+^ CTLs in the AMP@Zn/Mn‐MOF+αPD‐L1 group (Figure 8J,K), corroborating the findings from flow cytometric analysis (Figure 8D,E). Collectively, these results indicated that the combination of AMP@Zn/Mn‐MOF and αPD‐L1 enhanced CTL infiltration and effectively reprogrammed the immunosuppressive TME.

### 2.10 Combination Therapy Induced Systemic Antitumor Responses for Distant Tumor Inhibition

**FIGURE 9:**
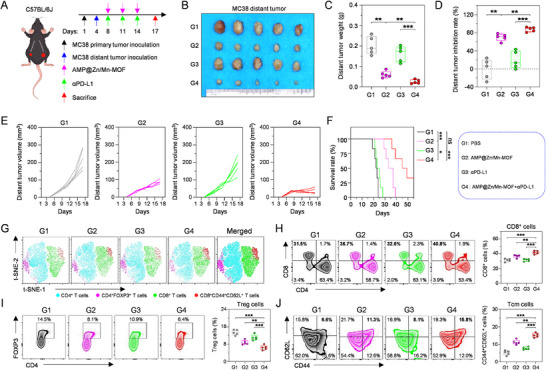
Combination therapy activated long‐term immune memory for distant tumor inhibition. (A) Schematic illustration of the experimental design for distant tumor immunotherapy. Created in Figdraw. (B) Digital images of distant tumors excised from each mouse following different treatments. (C) Profile of distant tumor weight in mice subjected to different treatments. n = 5. (D) Distant tumor inhibition rate in mice following different treatments. n = 5. (E) Growth curves of distant tumors in each mouse per group after different treatments. n = 5. (F) Survival curve of mice subjected to different treatments. n = 6. (G) t‐SNE plots of immune cells in distant tumor tissues following different treatments. (H) Flow cytometry analysis and relative quantification of infiltrating CD8^+^ T cells in distant tumor tissues after different treatments. n = 5. (I) Flow cytometry analysis and relative quantification of infiltrating regulatory T cells (CD4^+^FOXP3^+^) in distant tumor tissues after different treatments. n = 5. (J) Flow cytometry analysis and relative quantification of infiltrating central memory T cells (CD3^+^CD8^+^CD44^+^CD62L^+^) in distant tumor tissues after different treatments. n = 5. Data are presented as mean ± SD. Statistical significance was calculated by a two‐tailed unpaired Student's t‐test. Survival analysis was determined by Kaplan‒Meier analysis followed by the log‐rank test. *p < 0.05, **p < 0.01, ***p < 0.001; ns: no significant difference.

The observed immune activation prompted us to explore the impact of abscopal treatment on distant tumors. Notably, MC38 cells were subcutaneously implanted on the left side of the mice 4 days after the initial tumor inoculation on the right side. Three days postimplantation, the mice were randomly assigned to four groups (n = 5 per group): (G1) PBS, (G2) AMP@Zn/Mn‐MOF, (G3) αPD‐L1, and (G4) AMP@Zn/Mn‐MOF+αPD‐L1 (Figure 9A). Interestingly, αPD‐L1 monotherapy had no significant effect on the growth inhibition of distant tumors, whereas AMP@Zn/Mn‐MOF alone resulted in modest tumor growth suppression. In contrast, the combination of AMP@Zn/Mn‐MOF and αPD‐L1 significantly delayed tumor growth (Figure 9B–E, Figure S25). Compared with those in the other groups, the tumor weight and inhibition rate of distant tumors in the AMP@Zn/Mn‐MOF+αPD‐L1 group were notably greater (Figure 9B–D), highlighting the potential antitumor effects mediated through cGAS–STING pathway activation and ICD induction. Furthermore, compared with those in the other groups, the survival time of mice in the AMP@Zn/Mn‐MOF+αPD‐L1 group was significantly longer (Figure 9F).

We subsequently examined lymphocyte infiltration in distant tumors following the specified treatments (Figure S26). Two‐dimensional t‐distributed stochastic neighbor embedding (t‐SNE) analysis revealed that compared with the other groups, the AMP@Zn/Mn‐MOF+αPD‐L1 group exhibited heightened infiltration of CD8^+^ CTLs in distant tumors (Figure 9G). Flow cytometric analysis further confirmed that AMP@Zn/Mn‐MOF+αPD‐L1 treatment increased CD8^+^ CTL infiltration in distant tumors (Figure 9H). Additionally, the AMP@Zn/Mn‐MOF+αPD‐L1 group demonstrated a reduction in Treg cell populations within distant tumors (Figure 9I), confirming the potent systemic antitumor immunity elicited by AMP@Zn/Mn‐MOF+αPD‐L1. Given the importance of central memory T cells (T_cm_, CD3^+^CD8^+^CD44^+^CD62L^+^) in establishing long‐term immune responses [47], compared with the other groups, the AMP@Zn/Mn‐MOF+αPD‐L1 group showed a significant increase in T_cm_ populations (Figure 9J), indicating enhanced generation of immune memory cells. Collectively, these findings emphasize that the combination of AMP@Zn/Mn‐MOF and αPD‐L1 resulted in a robust systemic immune response, effectively combating tumors.

### 2.11 Biosafety of Combined Therapy with AMP@Zn/Mn‐MOF and αPD‐L1

To evaluate the biocompatibility of AMP@Zn/Mn‐MOF in combination with αPD‐L1, blood and tissue samples were obtained from tumor‐bearing mice at the end of the treatment regimen for comprehensive examination. First, serum biochemical assays were conducted to assess potential hepatic and renal toxicity. The levels of alanine aminotransferase (ALT), aspartate aminotransferase (AST), and blood urea nitrogen (BUN) in mice treated with different regimens all remained within the normal physiological ranges (Figure S27), indicating that the combination therapy had minimal adverse effects on liver or kidney function. Concurrently, histological examination of major organs (liver, heart, spleen, lungs, and kidneys) via H&E staining revealed no evident organ damage, indicating that the AMP@Zn/Mn‐MOF+αPD‐L1 treatment was well tolerated without significant toxic side effects (Figure S28). Hemolysis assays confirmed the excellent blood compatibility of AMP@Zn/Mn‐MOF, with no hemolysis observed in red blood cells even at concentrations up to 200 µg mL^−1^ (Figure S29). Throughout the treatment period, no significant change in body weight was observed, indicating negligible systemic toxicity (Figure S30). Moreover, AMP@Zn/Mn‐MOF had favorable colloidal stability in ultrapure water, PBS, and Dulbecco's modified Eagle's medium (DMEM) (Figure S31). Collectively, these findings demonstrated that the AMP@Zn/Mn‐MOF+αPD‐L1 combination therapy had excellent biocompatibility and biological safety.

## 3 Conclusions

We successfully engineered a novel pH/GSH‐responsive AMP@Zn/Mn‐MOF nanoplatform that enhanced tumor immunogenicity and facilitated robust antitumor immunity in synergy with ICB. Upon cellular uptake, the sustained release of Zn^2+^, Mn^2+^, and c‐di‐AMP diammonium activated the cGAS–STING signaling pathway, thereby increasing innate immune responses. Additionally, the chemodynamic therapeutic effect of AMP@Zn/Mn‐MOF induced ICD in tumor cells, resulting in the release of DAMPs such as LDH, HMGB‐1, and ATP, which further recruited immune cells. The dual effects of cGAS–STING pathway activation and ICD enabled AMP@Zn/Mn‐MOF to significantly inhibit the growth of murine MC38 tumors through the transformation of immunologically cold tumors into hot tumors. Mechanistic insights from RNA‐seq analysis revealed significant upregulation of activated immune response pathways and inhibition of cell proliferation in tumors treated with AMP@Zn/Mn‐MOF. Furthermore, the combination of AMP@Zn/Mn‐MOF with an αPD‐L1 antibody significantly enhanced antitumor efficacy, synergistically suppressing both primary and distant CC in mice by eliciting effective antitumor immunity. Given the critical role of the cGAS–STING pathway in tumor development [48], we investigated STING expression in human CC. Bioinformatics analysis of data from The Cancer Genome Atlas (TCGA) database revealed upregulated STING mRNA expression in the tumor tissues of CC patients (Figure S32A). Consistent with these findings, western blot analysis confirmed higher levels of STING in tumor tissues than in adjacent nontumor tissues (Figure S32B,C). In addition, in an HT‐29 xenograft model, AMP@Zn/Mn‐MOF significantly inhibited the tumorigenicity of human CC cells in vivo (Figure S33), highlighting the role of the cGAS–STING pathway as a promising target for clinical CC immunotherapy. Recent studies have also emphasized the potential of STING agonists to overcome resistance to immunotherapy and improve patient outcomes [49]. However, the effectiveness of STING activation in CC treatment is influenced by the genetic and epigenetic landscape of the tumor, as well as the presence of ICBs [50], and further studies are needed to improve the therapeutic outcomes for CC. Our study proved that Zn/Mn‐MOF exerts enzymatic activity with improved cGAS–STING activation ability, and the optimization of the Zn^2+^/Mn^2+^ molar ratio for the MOF is worthy of in‐depth exploration to obtain a higher catalytic efficiency with lower toxicity (Figure S5). In conclusion, we present an efficient and biocompatible platform for enhancing ICB‐related anticancer immunotherapy via activation of the cGAS–STING pathway and ICD to combat CC (Figure 1).

## 4 Materials and Methods

### 4.1 Preparation of Zn/Mn‐MOF

Zn/Mn‐MOF was prepared according to a previously reported method with slight modifications [23, 24]. Typically, 5 mmol Zn(NO_3_)_2_•6H_2_O and 5 mmol Mn(NO_3_)_2_•4H_2_O were added synchronously to 120 mL of methanol solution and stirred for 30 min. Then, 120 mL of methanol containing 2‐methylimidazole (40 mmol) was added dropwise to the above mixture and stirred for 4 h. Afterward, the mixture was maintained at 50°C for 1 h and then centrifuged at 10 000 × *g* for 15 min. The resulting precipitates were separated, washed twice with ethanol, and finally dried under vacuum at 60°C.

### 4.2 Synthesis of AMP@Zn/Mn‐MOF

The as‐prepared Zn/Mn‐MOF (10 mg) was dispersed into 10 mL of ultrapure water by sonication. Afterward, 20 mg of c‐di‐AMP diammonium was dissolved in the suspension by sonication for 3 min. Afterward, 10 mg of DSPE‐PEG_2000_‐NHS was added, and the mixture was stirred for another 6 h at room temperature. The product was collected by centrifugation (10 000 × *g*, 15 min) and washed with ultrapure water several times.

### 4.3 Measurement of POD‐mimetic and GSHox‐mimetic Activity

Mixed solutions containing AMP@Zn/Mn‐MOF ([Mn] = 0.1 mM), H_2_O_2_ (3 mM), GSH (10 mM), and MB (10 µg mL^−1^) with a total volume of 1 mL in NaHCO_3_ buffer (25 mM) were prepared and kept at 37°C for 30 min. Then, the •OH‐induced MB degradation was evaluated by the change in absorbance at 652 nm via UV‐vis spectroscopy [26]. The generation of •OH was measured on an EPR spectrometer (JES‐FA200, JEOL, Japan) using DMPO as the •OH spin‐trapping agent.

The ability of AMP@Zn/Mn‐MOF to eliminate GSH was determined using DTNB as a GSH indicator [27]. After GSH (10 mM) and DTNB (1 mM) were added to AMP@Zn/Mn‐MOF solutions, the mixed solution was incubated for 24 h, and the absorbance at 410 nm was recorded.

### 4.4 3D Tumor Spheroid Formation

To construct 3D MC38 tumor spheroids, cells were seeded at a density of 10^3^ cells per well in low‐adhesion 96‐well plates. The culture medium was changed every two days with gentle agitation to ensure uniform spheroid formation, and the spheroids were cultured for 4 days prior to experimental use. Afterward, PBS, c‐di‐AMP diammonium (2.8 µg mL^−1^), Zn/Mn‐MOF (7 µg mL^−1^), or AMP@Zn/Mn‐MOF (7 µg mL^−1^) was added to the 96‐well plates, followed by a 3‐day incubation period. The morphology of the 3D tumor spheroids was visualized via microscopy.

### 4.5 Western Blot Analysis

Western blot analysis was performed to evaluate the expression levels of cGAS, STING, p‐STING, TBK1, p‐TBK1, IRF3, p‐IRF3, NF‐κB, p‐NF‐κB, and β‐actin. MC38 cells were cultured in 6‐well plates and incubated for 24 h following treatment with PBS, c‐di‐AMP diammonium (2.8 µg mL^−1^), Zn/Mn‐MOF (7 µg mL^−1^), or AMP@Zn/Mn‐MOF (7 µg mL^−1^). The treated cells were then collected and washed twice with PBS. Cell lysis was achieved using RIPA buffer supplemented with 1% protease and phosphatase inhibitors for 1 h. The lysates were subsequently centrifuged at 12,000 rpm for 15 min at 4°C to isolate the proteins. The protein concentration was determined using a BCA protein assay kit.

The protein samples were separated by 10% sodium dodecyl sulfate‒polyacrylamide gel electrophoresis (SDS‒PAGE) using Tris‒glycine running buffer. The separated proteins were then transferred to polyvinylidene fluoride (PVDF) membranes using a gel electrophoretic transfer apparatus (Bio‐Rad, USA). The PVDF membranes were blocked with 5% bovine serum albumin (BSA) in TBST and incubated overnight at 4°C with primary antibodies as recommended by the manufacturer. The membranes were subsequently washed three times with TBST and incubated with horseradish peroxidase (HRP)‐conjugated secondary antibodies for 2 h at room temperature. Finally, western blot images were captured using a Bio‐Rad gel documentation system after incubation with an enhanced chemiluminescence (ECL) substrate.

### 4.6 CCK‐8 Assay

A CCK‐8 assay was used to assess the cytotoxicity of the various formulations. Different cell lines, including MC38, LOVO, DLD‐1, and BEND.3, were plated in 96‐well plates at a density of 2 × 10^4^ cells per well and cultured overnight. The culture medium was then replaced with fresh media containing AMP@Zn/Mn‐MOF at various concentrations. Following a 24‐h incubation period, 10 µL of CCK‐8 reagent was added to each well. The absorbance was subsequently measured at a wavelength of 450 nm using a microplate spectrophotometer (Tecan, Switzerland).

### 4.7 Immunofluorescence Staining Assay

MC38 cells were plated in CLSM‐exclusive culture dishes at a density of 1 × 10^5^ cells per dish and incubated for 24 h to allow cell adhesion. Following the indicated treatments for 24 h, the cells were fixed with precooled 4% paraformaldehyde solution for 15 min and then blocked with a solution containing 5% BSA and 0.25% Triton X‐100 for 30 min. The fixed cells were subsequently incubated overnight at 4°C with primary antibodies targeting p‐TBK1, p‐IRF3, HMGB‐1, and CRT. After being washed, the cells were stained with secondary antibodies for 2 h at room temperature. The nuclei were then stained with DAPI for 10 min. Fluorescence images were captured using a confocal laser scanning microscope (CLSM, Leica, Germany) and analyzed using ImageJ software (NIH, Bethesda, MD, USA).

### 4.8 Tumor Model

Male C57BL/6J mice (4 weeks old) were obtained from Vital River Laboratory Animal Technology Co., Ltd. All animal procedures were approved by the Institutional Animal Care and Use Committee of Wenzhou Institute, University of Chinese Academy of Sciences (Approval number: WIUCAS24090902). The mice were maintained in specific‐pathogen‐free (SPF) rodent facilities. To establish MC38 tumor‐bearing models, 2 × 10^6^ MC38 cells were implanted into the right flanks of the mice. The tumor volume was calculated using the following formula: tumor volume (mm^3^) = width^2^ × length/2.

### 4.9 In Vivo Antitumor Study

When the tumor volume reached approximately 100 mm^3^, the MC38 tumor‐bearing mice were randomly assigned to six groups (n = 5 per group) and treated with intravenous injections of PBS, Zn/Mn‐MOF (100 µg per mouse), c‐di‐AMP diammonium (40 µg per mouse), AMP@Zn/Mn‐MOF (100 µg per mouse), αPD‐L1 (100 µg per mouse), or AMP@Zn/Mn‐MOF (100 µg per mouse)+αPD‐L1 (100 µg per mouse). The treatments were administered three times, with a 3‐day interval between each injection. The body weights and tumor volumes of the mice were monitored every 3 days. At the end of the treatment period, the mice were euthanized, and the tumors and major tissues (heart, liver, spleen, lung, and kidney) were harvested for histological examination. The mice were humanely sacrificed if the tumor volume reached 2 000 mm^3^ during the study.

### 4.10 Biodistribution Analysis

MC38 tumor‐bearing mice (n = 3) were intravenously injected with FITC or FITC@Zn/Mn‐MOF (5 µg per mouse). Fluorescence images of the mice were captured at specified time points (0, 2, 8, 24, and 48 h) postinjection using the Perkin Elmer IVIS Lumina III system. At 48 h postinjection, the mice were euthanized, and major organs (heart, liver, spleen, lungs, and kidneys) and tumors were collected for *ex vivo* biodistribution imaging analysis.

### 4.11 Transcriptome Sequencing

Tumor tissues treated with PBS and AMP@Zn/Mn‐MOF were frozen and thoroughly cryoground in TRIzol reagent. RNA extraction was performed according to the manufacturer's instructions. The purified mRNA samples were then processed for fragmentation, reverse transcription, polymerase chain reaction (PCR), library construction, and sequencing using a NovasSeq 6000 system (Illumina, USA) at LC‐Biotechnology (Hangzhou, China).

### 4.12 Flow Cytometry Analysis

Tumor tissues were digested following the manufacturer's protocols and then centrifuged and separated to obtain a single‐cell lymphocyte suspension for further analysis [51]. Various immune cell populations were stained with antibodies (Table S1) according to the manufacturer's instructions. Flow cytometry data were acquired using an Agilent Technologies flow cytometer, and the results were analyzed with FlowJo software (version 10.9; Ashland, USA).

### 4.13 Immunofluorescence Staining of Tumor Tissues

The harvested tissues were embedded in Tissue‐Tek O.C.T. Compound and sectioned into slices using a freezing microtome. The tissue sections were then blocked with a solution containing 5% BSA and 0.25% Triton X‐100, washed, and stained with anti‐mouse CD8 antibody. Fluorescence imaging of the tissue sections was performed using a CLSM(Leica, Germany).

### 4.14 H&E and Immunohistochemistry (IHC) Staining

The resected tumors and major organs were fixed in a 4% paraformaldehyde solution and subsequently embedded in paraffin. The tumor sections were dewaxed, rehydrated, and stained with H&E. For IHC staining, the tissue sections were subjected to antigen retrieval using sodium citrate buffer (pH 6.0), after which endogenous peroxidase activity was blocked with 3% H_2_O_2_. After permeabilization with 0.25% Triton X‐100 and blocking with 5% goat serum, the sections were incubated with primary antibodies overnight at 4°C. The slides were then washed and incubated with secondary antibodies for 2 h at room temperature. Immunoreactive sites were visualized using 3,3′‐diaminobenzidine (DAB) and counterstained with hematoxylin. The slides were subsequently dehydrated through graded alcohol concentrations, and images were captured using a pathological scanner (KF‐PRO‐005).

### 4.15 ELISA

Ocular venous blood samples collected from treated mice were allowed to clot spontaneously at 4°C for 2 h. The samples were then centrifuged at 2000 × *g* for 20 min at 4°C to isolate the serum. ELISA kits were used to analyze cytokine levels and blood biochemistry following the manufacturer's instructions.

### 4.16 Statistical Analysis

All experimental data are presented as the mean ± standard deviation (SD). Two‐group comparisons were analyzed using a two‐tailed unpaired Student's *t*‐test. Statistical analyses were performed using GraphPad Prism 10 (GraphPad Software Inc., USA). In all the cases, *p* values < 0.05 were considered to indicate statistical significance. The significance levels are denoted as follows: *p < 0.05, **p < 0.01, ***p < 0.001, and not significant (ns.).

## Conflicts of Interest

The authors declare no conflicts of interest.

## Supporting information




**Supporting File**: advs74121‐sup‐0001‐SuppMat.docx.

## Data Availability

The data that support the findings of this study are available in the supplementary material of this article.
