# Supplementary material for: Synergistic Activation of Immunogenic Cell Death and the cGAS–STING Pathway by Engineered Zinc/Manganese‐Based Metal–Organic Framework Nanoplatforms for Colon Cancer Immunotherapy
Source: Adv Sci (Weinh). 2026 Jan 28;13(19):e21146. doi: 10.1002/advs.202521146 (PMC13045217; doi:10.1002/advs.202521146)
Supplement: Supplementary file 1 — Supporting File: advs74121‐sup‐0001‐SuppMat.docx. [file ADVS-13-e21146-s001.docx]

**Supporting information**

1. Characterization

Hydrodynamic size and zeta potential analysis were performed using a Malvern Nano Analyzer. Morphology and corresponding EDS spectra of the NPs were obtained using a ZEISS Gemini SEM 300. XPS spectra were obtained using a Thermo Scientific K-Alpha instrument. XRD was carried out with a Rigaku Ultima IV spectrometer. UV−vis absorption spectra were recorded using a MAPADA spectrometer. FTIR spectra were recorded using a Thermo Scientific Nicolet iS20 spectrometer. The Brunauer−Emmett−Teller surface area and pore size distribution of the NPs were measured using a Micromeritics ASAP 2460. The thickness of the NPs was measured using a Bruker Dimension Icon microscope.

2. Drug loading efficiency

The conventional absorbance-concentration calibration curve for c-di-AMP diammonium was constructed using a UV–vis spectrophotometer at 258 nm. After centrifugation (10,000 × g, 10 min), the loading capacity was determined by quantifying free c-di-AMP diammonium in the supernatant. The drug loading content (LC%) of c-di-AMP diammonium was calculated using the following equation:

LC% = M_Encapsulated_ _c-di-AMP diammonium mass_ /M_Initial_ _c-di-AMP diammonium mass_ × 100%.

3. pH-responsive release

To assess pH-responsive release profiles of Zn^2+^, Mn^2+^, and c-di-AMP diammonium, AMP@Zn/Mn-MOF was incubated in different NaAc solutions (pH = 4.5, 5.5, 6.5, 7.5) under horizontal shaking at 100 rpm at room temperature. Following incubation, the solution was centrifuged (10,000 × g, 15 min), and then the supernatant was collected. The released Zn^2+^, Mn^2+^, and c-di-AMP diammonium were measured using ICP‒MS and UV−vis spectrometer, respectively.

4. Bio-TEM

For TEM observation, the MC38 cells were collected and fixed using glutaraldehyde for 2 h to prepare samples for bio-TEM observation (Hitachi HT-7800, Japan).

5. RT-qPCR

After the indicated treatment, the cells were collected. mRNA was extracted, and the expression of the cGAS-STING pathway-regulated gene (IFN-β) was detected by RT-qPCR analysis.

6. Hemolysis

The obtained red blood cells were then added to PBS to prepare 2% red blood cell solution. Afterward, different concentrations of AMP@Zn/Mn-MOF were added to the erythrocyte suspension and incubated for 4 hours at 37 °C, followed by centrifugation to precipitate the erythrocytes at the bottom of the Eppendorf tubes. Finally, the absorbance of the supernatant at 545 nm was tested using a multifunctional microplate reader.

7. Intracellular ROS and GSH detection

MC38 cells were seeded into CLSM-exclusive culture dishes (1 × 10^5^ cells per dish) and incubated for 24 h to adhere. After the indicated therapies for 24 h, DCFH-DA, MitoPeDPP, and ThiolTracker were added into dishes and incubated for 15 min, then the intracellular ROS and GSH in the treated cells were evaluated by CLSM (Leica, Germany).

8. Cell lines

Mouse colon carcinoma cell line (MC-38, RRID: CVCL_B288)), mouse macrophages transformed by leukemia virus (RAW264.7, RRID: CVCL_0493), mouse brain-derived endothelial cell line (BEND.3, RRID: CVCL_0170), human colon cancer cell line (LOVO, RRID: CVCL_0399), Mouse Dendritic Cell Line (DC2.4, RRID: CVCL_J409), Human Colon Carcinoma Cell line (HT-29, RRID: CVCL_0320), and human colorectal cancer cell lines (DLD-1, RRID: CVCL_0248) were obtained from the National Infrastructure of Cell Line Resource (Beijing, China. <http://cellresource.cn>). All cells were tested for mycoplasma monthly, and only mycoplasma-free cells were used in this study. These cells were maintained in Roswell Park Memorial Institute (RPMI) 1640 medium or Dulbecco’s modified Eagle’s medium (DMEM), which were supplemented with 10% fetal bovine serum (FBS) and 1% penicillin/streptomycin. All cell lines were cultured at 37 °C in a humidified atmosphere containing 5% CO_2_. When the cells reached approximately 70% confluence, they were employed for both *in vitro* and *in vivo* experimental procedures.

9. Cellular uptake

MC38 cells were plated in CLSM-exclusive culture dishes (1 × 10^5^ cells per dish) and cultured overnight to achieve adherence. The cells were then incubated with FITC@Zn/Mn-MOF (1 μg/mL) for a duration of 6 hours. After treatment, the cells were rinsed with PBS, and Hoechst 33342 and LysoTracker were introduced into the dishes for a 15-minute staining period. The fluorescence was visualized using Leica CLSM or analyzed via a Beckman Coulter flow cytometer.

10. Intracellular iron content detection

MC38 cells were plated into 6-well plates (1 × 10^6^ cells per well) and cultured for 12 hours. Subsequently, the cells were treated with AMP@Zn/Mn-MOF (1 μg/mL) for an additional 6 hours. Following this, the cells were washed three times with PBS. Next, the cells were lysed using a 4% nitric acid solution at 60 °C for 8 hours. The resulting lysates were resuspended in ultrapure water, and the concentrations of intracellular Zn^2+^ and Mn^2+^ were quantified using an ICP-MS instrument.

11. Live/dead staining assay

MC38 cells were plated into CLSM-exclusive culture dishes (1 × 10^5^ cells per dish) and incubated for 24 hours to adhere. Subsequently, the cells were exposed to various medium containing PBS, c-di-AMP diammonium (2.8 μg/mL), Zn/Mn-MOF (7 μg/mL), or AMP@Zn/Mn-MOF (7 μg/mL) for a period of 24 hours. Following treatment, the cells were co-stained with a calcein-AM/PI working solution for 15 minutes. The stained cells were then examined using CLSM (Leica, Germany).

12. Cell apoptosis assay

MC38 cells were seeded into 12-well plates (5 × 10^5^ cells per well) and cultured overnight. Subsequently, the cells were exposed to PBS, c-di-AMP diammonium (2.8 μg/mL), Zn/Mn-MOF (7 μg/mL), or AMP@Zn/Mn-MOF (7 μg/mL) for a period of 24 hours. Following treatment, the cells were rinsed with PBS, harvested, and stained using the Annexin V-FITC and PI apoptosis detection kits according to the manufacturer’s instructions. Apoptosis was assessed by flow cytometry, and the data were analyzed using a flow cytometer.

13. LDH and ATP Assays

MC38 cells were seeded in 96-well plates at a density of 1 × 10^4^ cells per well and incubated overnight. Following treatment with PBS, c-di-AMP diammonium (2.8 μg/mL), Zn/Mn-MOF (7 μg/mL), or AMP@Zn/Mn-MOF (7 μg/mL) for 24 hours, the levels of extracellular ATP and LDH were measured using the LDH and ATP Assay Kit (Dojindo, Japan) in accordance with the manufacturer’s instructions.

14. Colony Formation Assay

MC38 cells were plated in 12-well plates at a density of 8 × 10^2^ cells per well and cultured for 3 days. Subsequently, the culture medium was replaced with fresh medium supplemented with PBS, c-di-AMP diammonium (2.8 μg/mL), Zn/Mn-MOF (7 μg/mL), or AMP@Zn/Mn-MOF (7 μg/mL) for an additional 2 days. Once colonies were visible, cells were fixed with a 4% paraformaldehyde solution for 15 min and subsequently stained with crystal violet for 15 min.

15. Transwell migration assay

MC38 cells were seeded in 6-well plates at a density of 2 × 10^6^ cells per well and cultured overnight. The cells were then treated with medium containing PBS, c-di-AMP diammonium (2.8 μg/mL), Zn/Mn-MOF (7 μg/mL), or AMP@Zn/Mn-MOF (7 μg/mL) for 24 hours. Following treatment, the medium was collected and centrifuged at 12,000 × g for 15 minutes at 4 °C. The resulting supernatant was added to the lower compartment of a transwell chamber, while RAW264.7 cells were plated in the upper compartment at a density of 1 × 10^4^ cells per well. After a 24-hour incubation, the migrated RAW264.7 cells were stained with crystal violet and imaged using an inverted microscope.

16. TCGA

The normalized gene expression RNA-seq of CC patients was obtained from TGCA (<https://portal.gdc.cancer.gov/>).

17. Human clinical samples

Colon cancer patient samples (n = 5) were collected from The First Affiliated Hospital of Wenzhou Medical University. All human cancer tissues used in this study were approved by the Institutional Review Board of The First Affiliated Hospital of Wenzhou Medical University. Written informed consent was obtained from each patient before sample collection.

18. BMDM isolation

Bone marrow cells were harvested from the femurs of 6-week-old male C57BL/6J mice and cultured in complete medium (RPMI1640 medium supplemented with 10% FBS, 1% penicillin/streptomycin, 2 mM L-glutamine, 10 mM HEPES, 1 mM sodium pyruvate, 1×nonessential amino acids, 50 μM β-mercaptoethanol, and 20 ng/mL M-CSF). After 7 days of incubation, BMDMs were seeded in a 12-well plate at 5 × 10^5^ cells/well and treated with the indicated medium. Data were collected and analyzed on the flow cytometer.

19. HT-29 xenograft model

HT-29 human colorectal cells (2 × 10^6^ cells/mouse) were subcutaneously injected into the right ﬂank of 4-week-old male NOD SCID mice. The mice were divided randomly into two groups (n = 5 per group) and were intravenously injected with PBS or AMP@Zn/Mn-MOF (100 μg per mouse). The treatments were administered three times, with a 3-day interval between each injection. The tumor volumes of the mice were monitored every 3 days. At the end of the treatment period, the mice were euthanized, and the tumors were harvested for further examination.


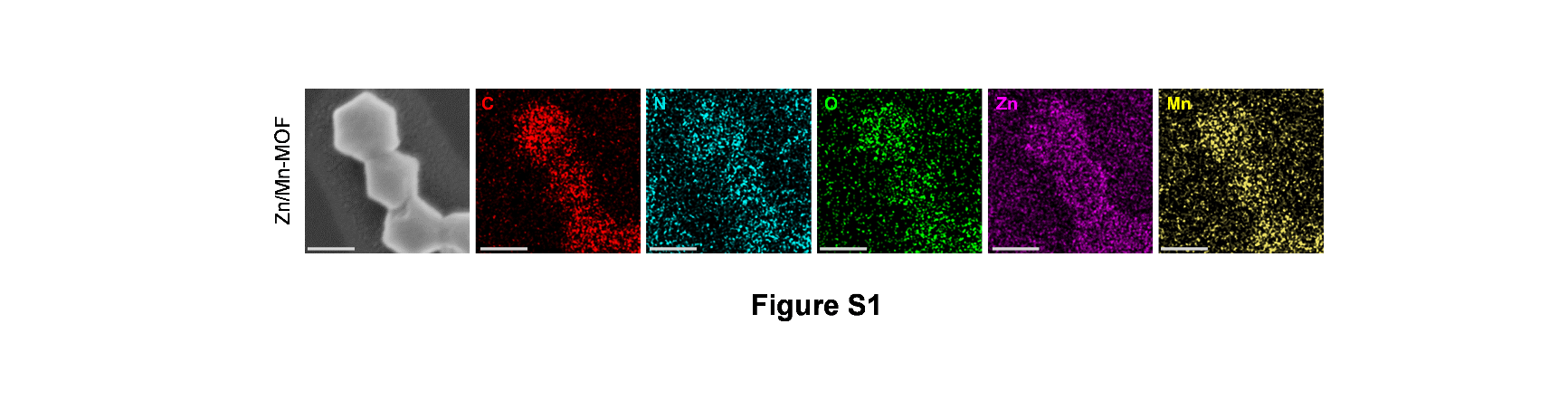
**Figure S1.** EDS elemental maps of Zn/Mn-MOF. Scale bar: 100 nm.


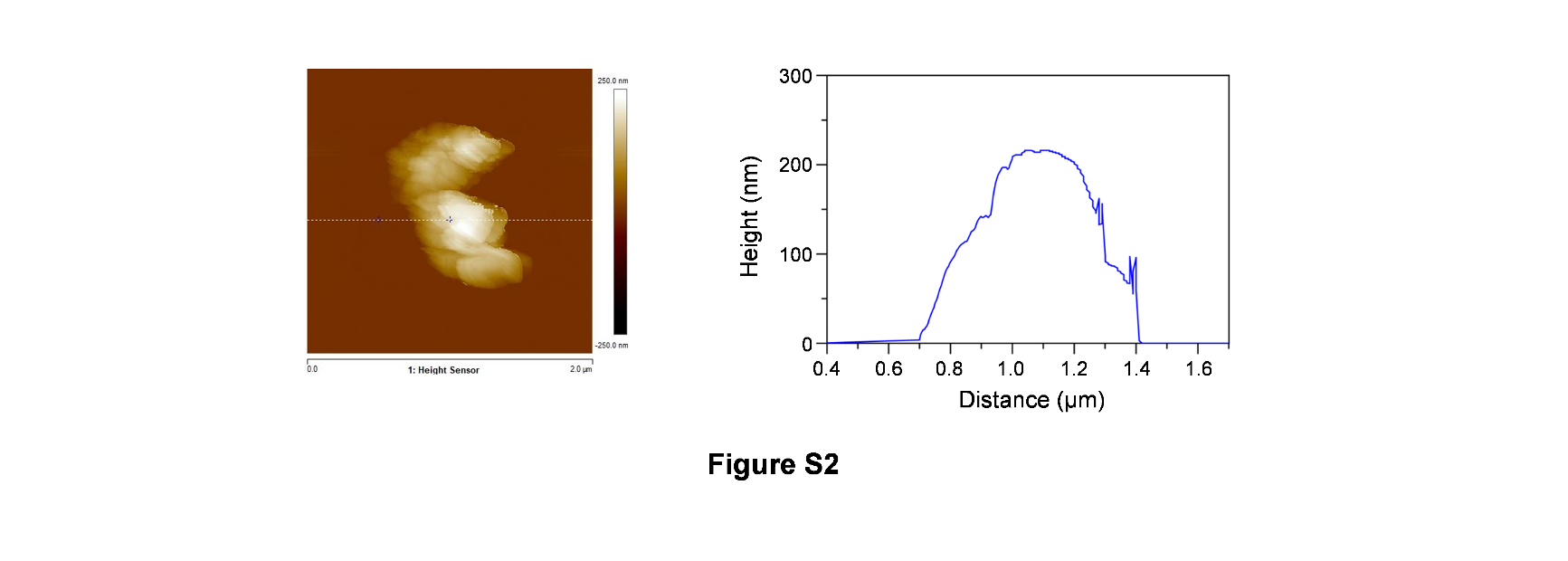
**Figure S2.** AFM image and the particle thickness of AMP@Zn/Mn-MOF.


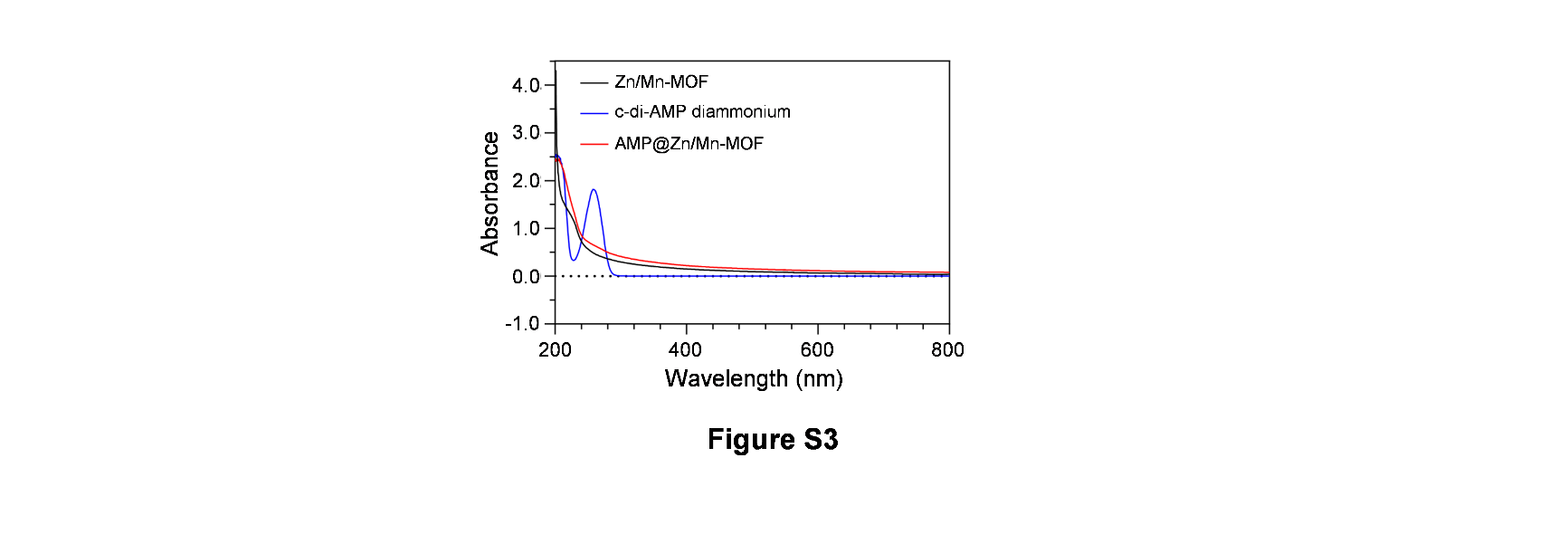
**Figure S3.** UV−vis spectra of Zn/Mn-MOF, c-di-AMP diammonium, and AMP@Zn/Mn-MOF.


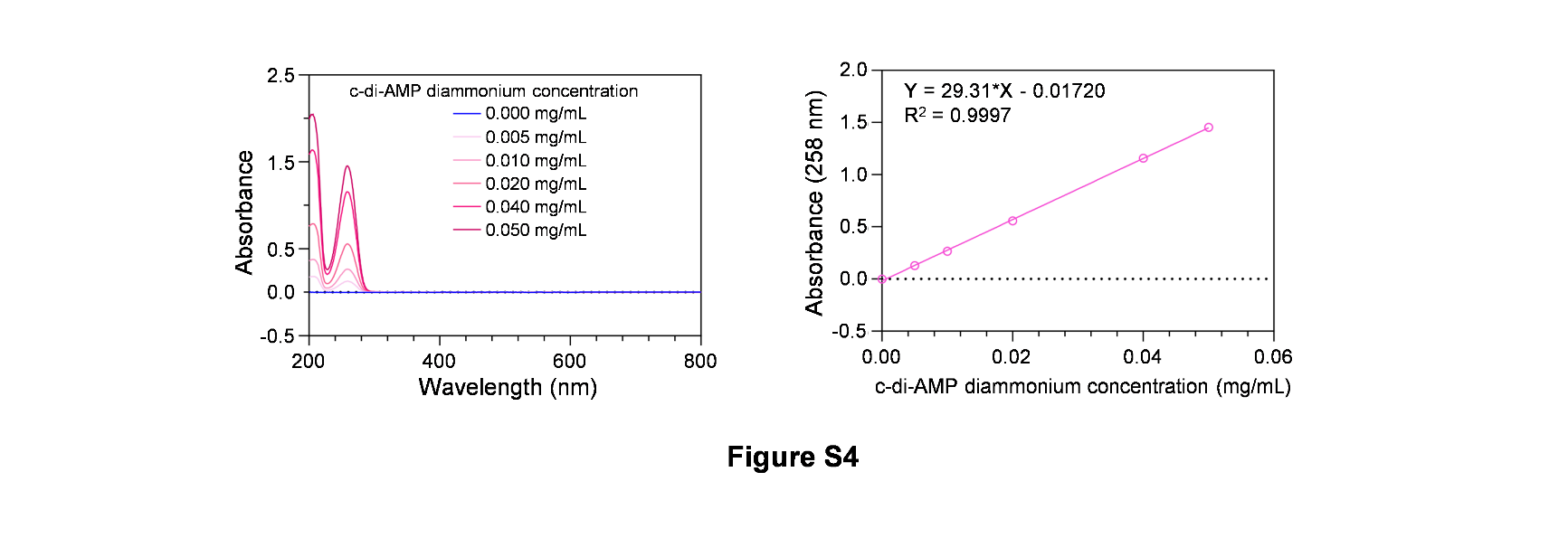
**Figure S4.** The standard curve of the absorption intensity (258 nm) of c-di-AMP diammonium as a function of concentration. n = 3. Data are presented as mean ± SD.


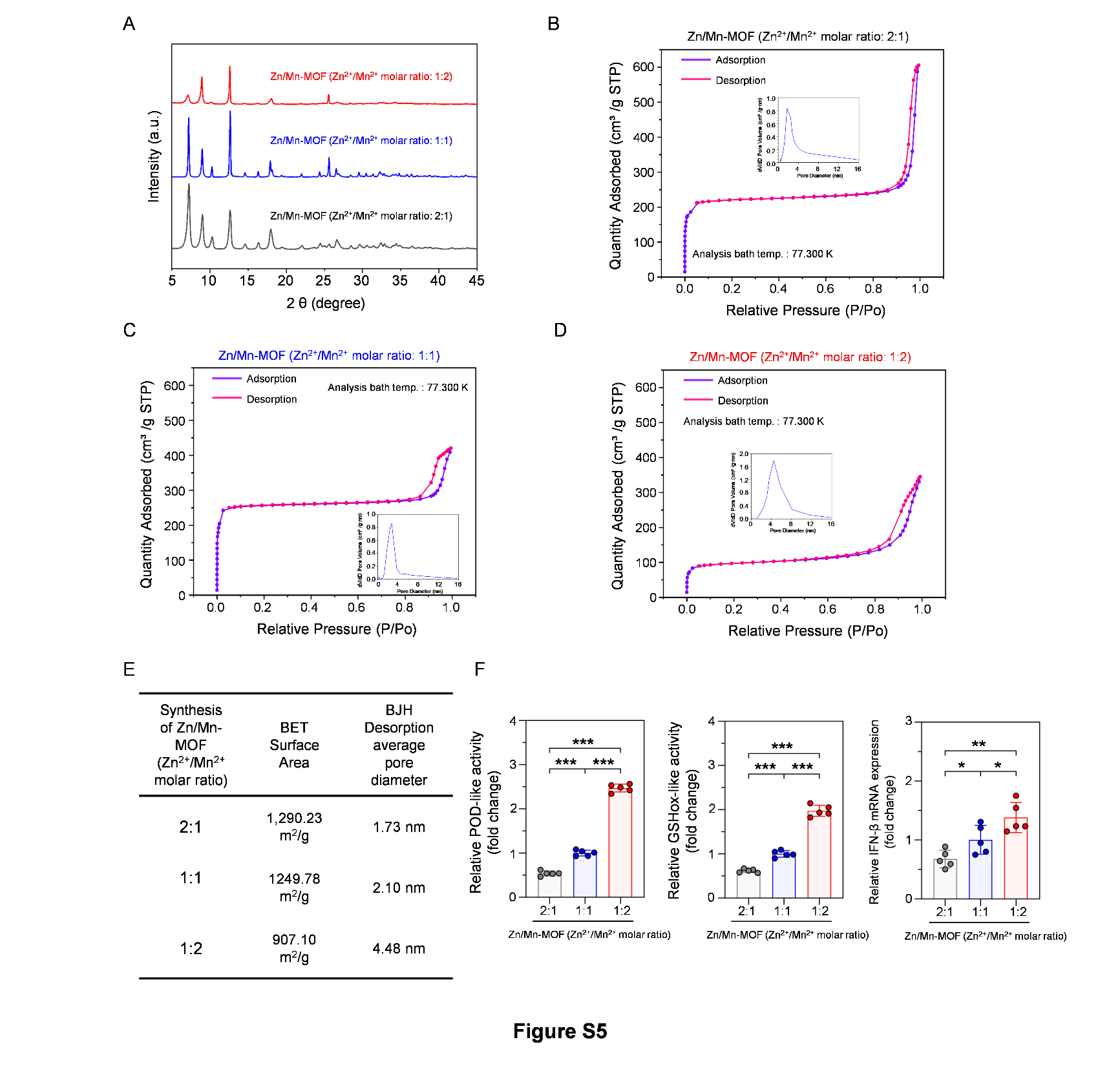
**Figure S5.** Characterization, catalytic activity, and cGAS–STING activation ability of Zn/Mn-MOF. (A) XRD patterns of Zn/Mn-MOF. (B-E) BET N_2_ adsorption/desorption isotherms and pore distribution curves of Zn/Mn-MOF. (F) Quantitative analysis of POD-like/GSHox-like activity and cGAS–STING activation ability of Zn/Mn-MOF. Data are presented as mean ± SD. Statistical significance was calculated by a two-tailed unpaired Student's t-test. *P < 0.05, **P < 0.01, ***P < 0.001.


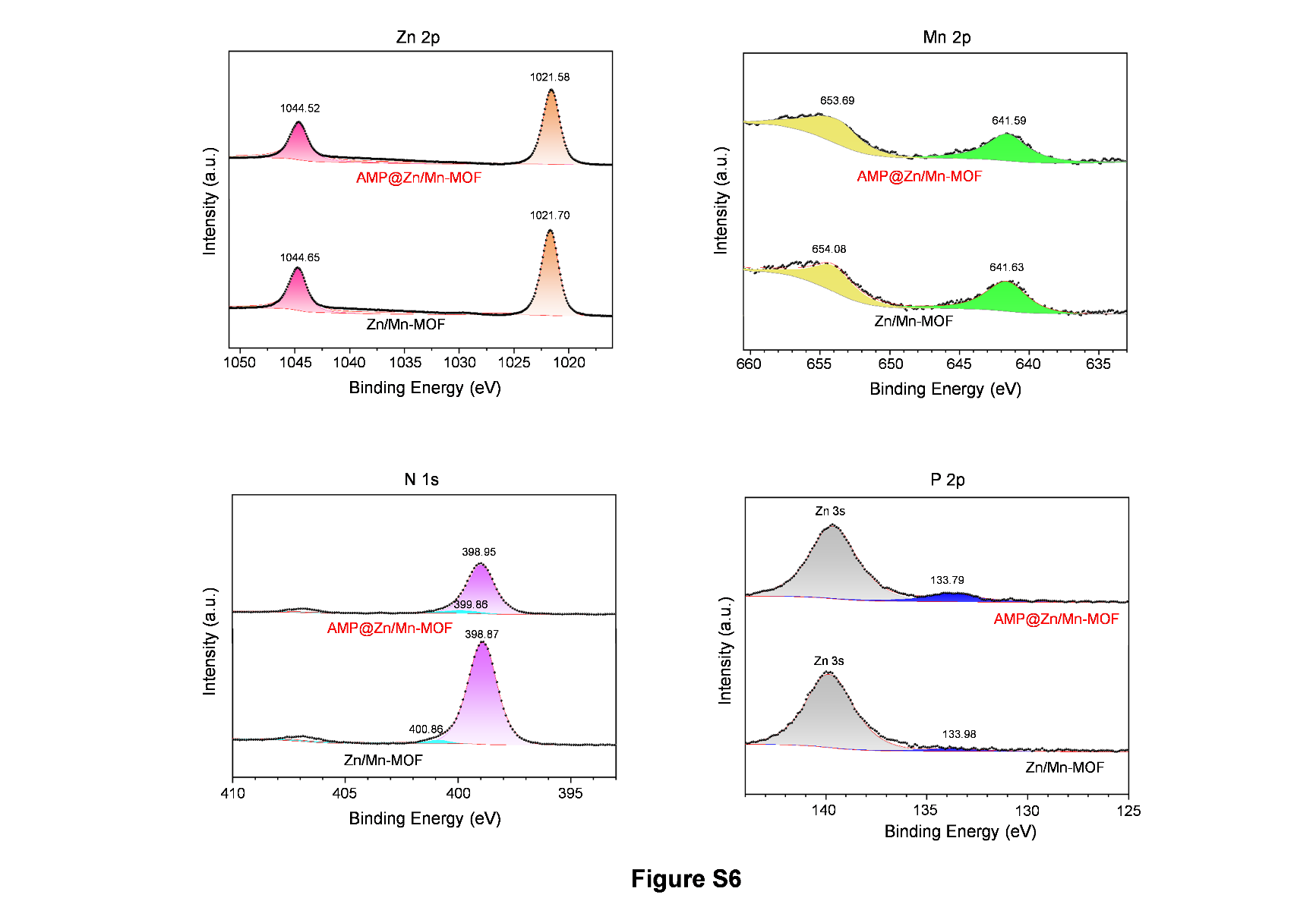


**Figure S6.** High-resolution XPS spectra of Zn 2p, Mn 2p, N 1s, and P 2p in Zn/Mn-MOF and AMP@Zn/Mn-MOF.


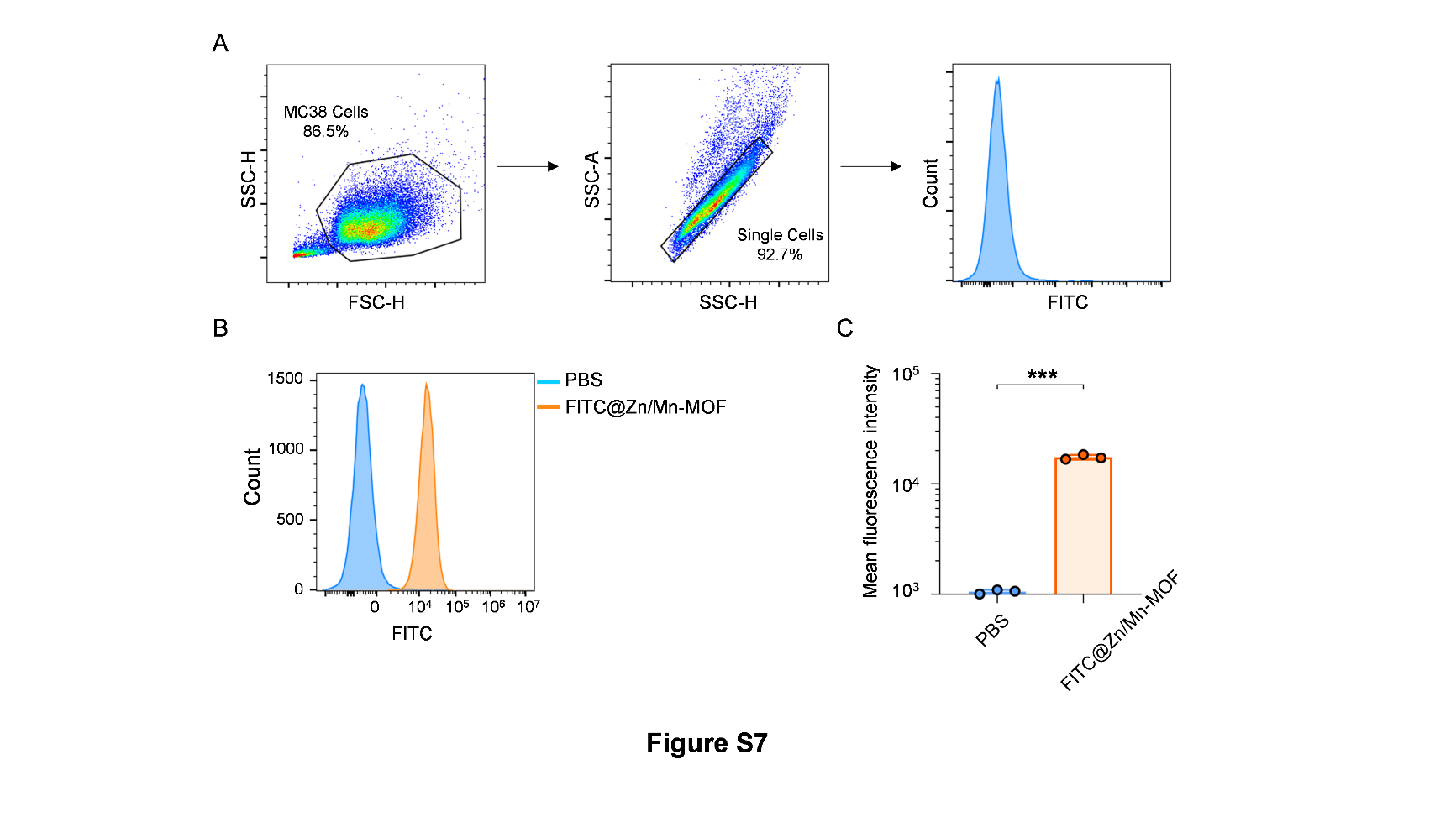
**Figure S7.** Flow cytometry analysis of MC38 cells treated with FITC@Zn/Mn-MOF, showing the corresponding mean intracellular fluorescence intensity. n = 3. Data are presented as mean ± SD. Statistical significance was calculated by a two-tailed unpaired Student's t-test. ***P < 0.001.


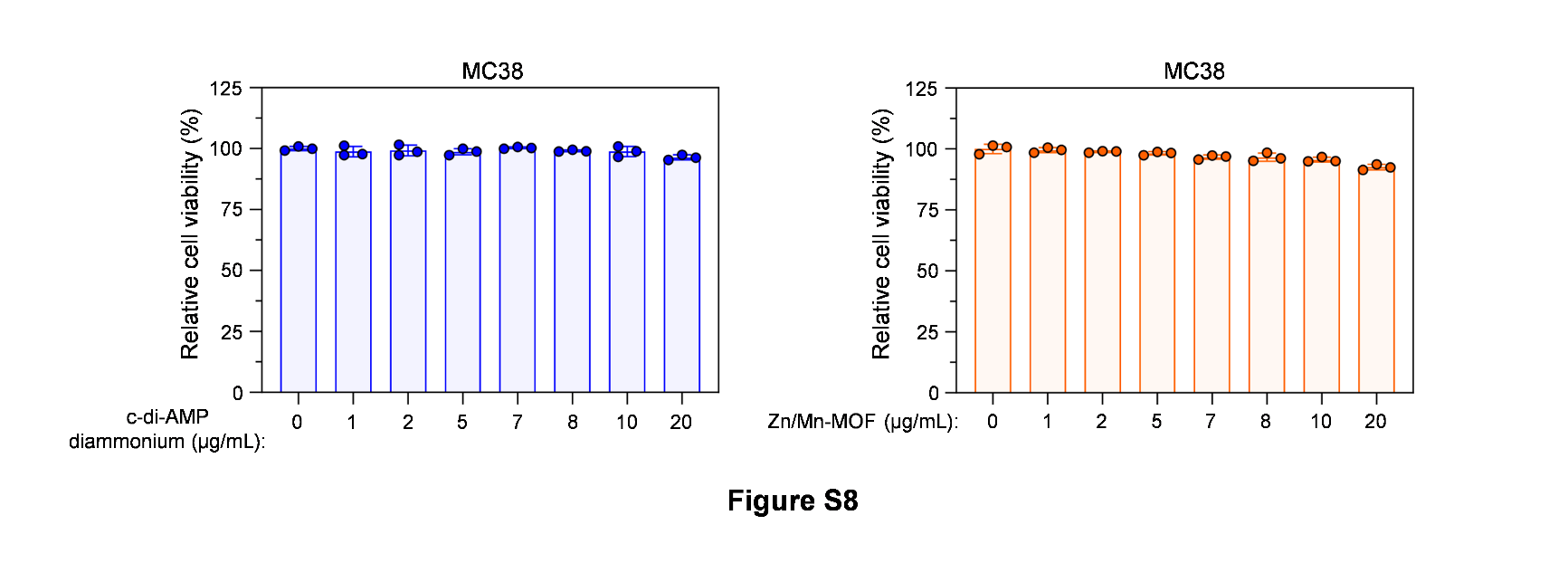
**Figure S8.** Cytotoxic effects on MC38 cells after exposure to various concentrations of c-di-AMP diammonium and Zn/Mn-MOF. n = 3. Data are presented as mean ± SD.


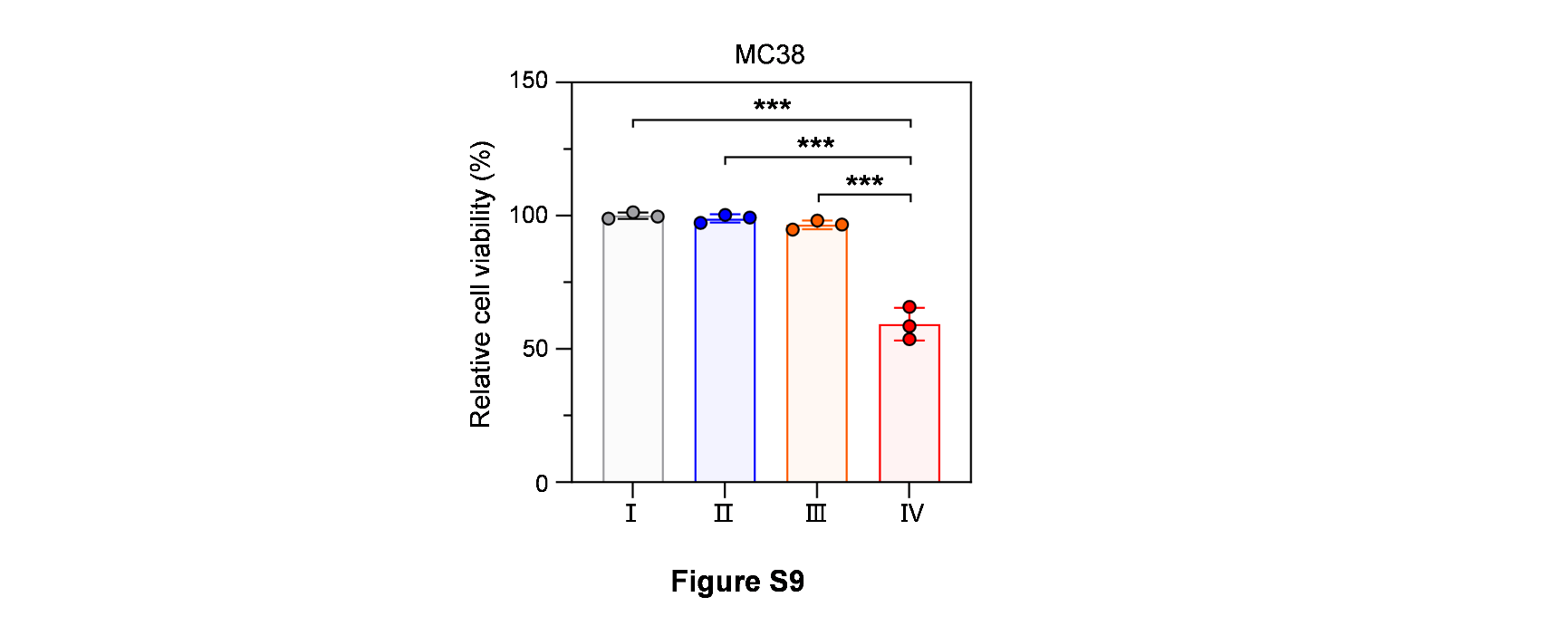
**Figure S9.** Cytotoxicity of MC38 cells following different treatments. n = 3. Data are presented as mean ± SD. Statistical significance was calculated by a two-tailed unpaired Student's t-test. ***P < 0.001.


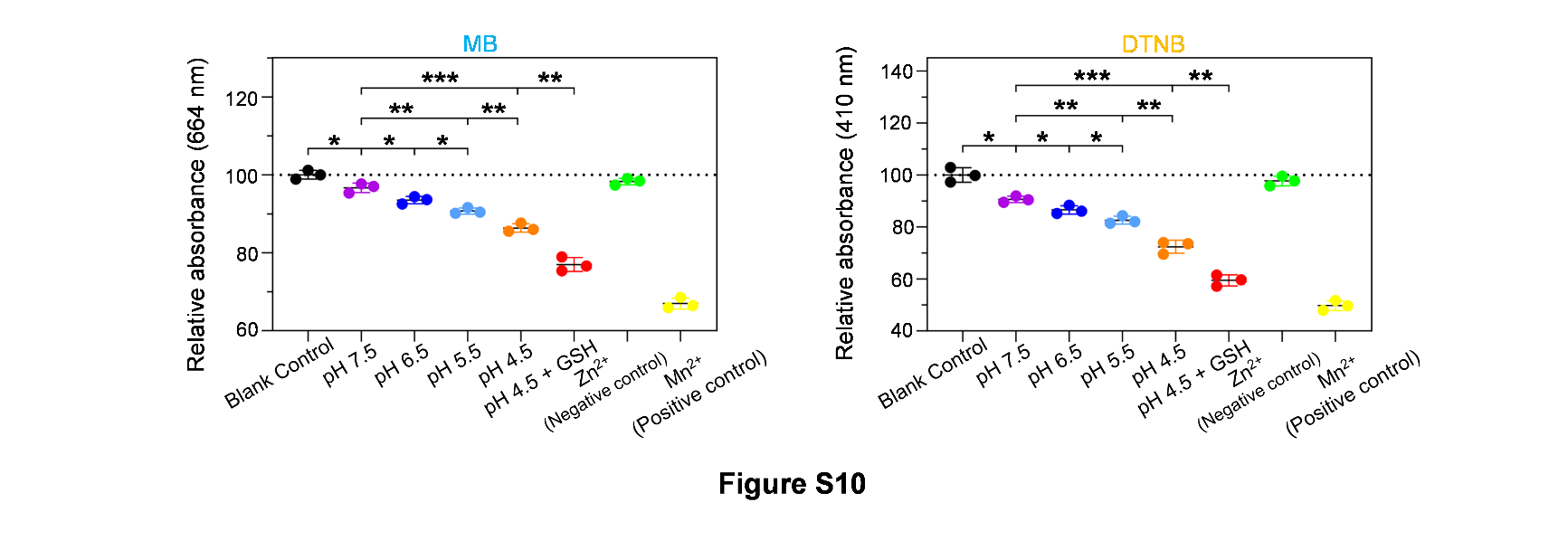
**Figure S10.** Determination of POD-mimetic and GSHox-mimetic activity of AMP@Zn/Mn-MOF. n = 3. Data are presented as mean ± SD. Statistical significance was calculated by a two-tailed unpaired Student's t-test. *P < 0.05, **P < 0.01, ***P < 0.001.


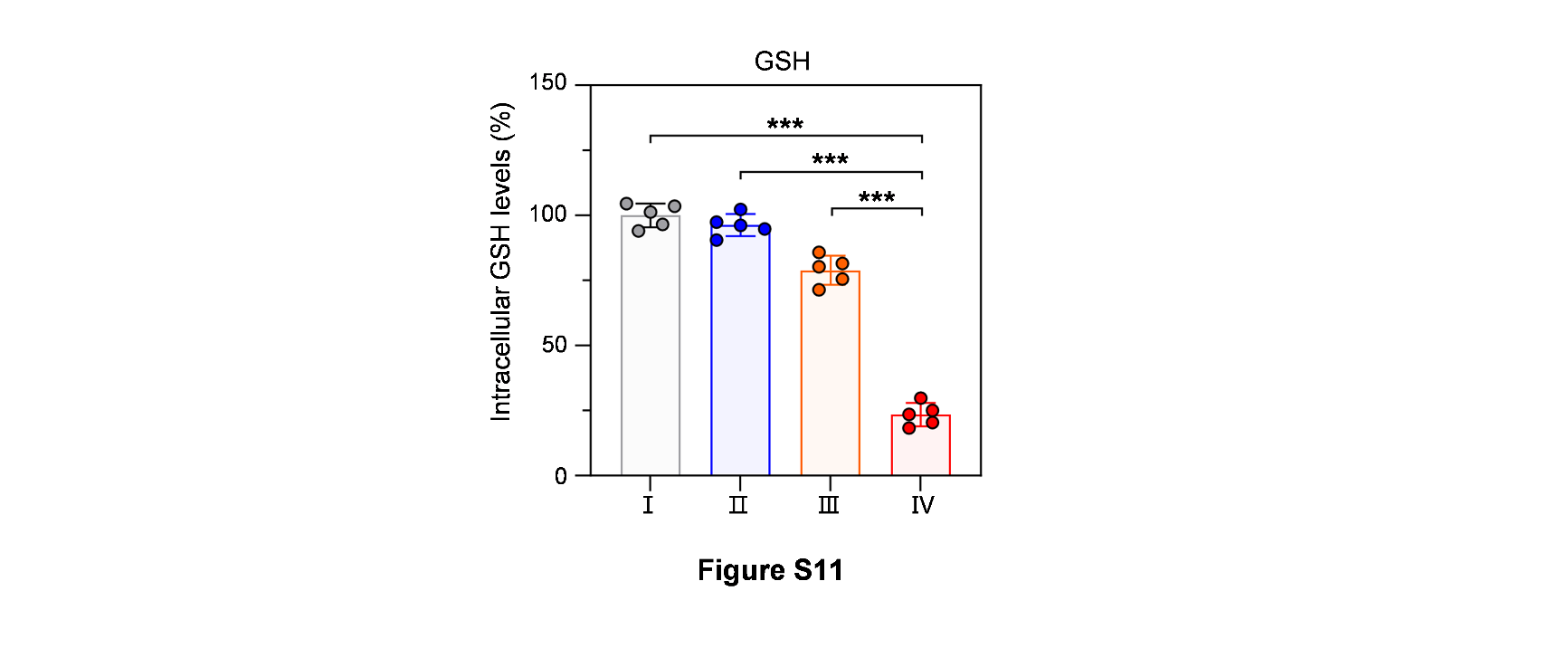
**Figure S11.** Intracellular GSH level in MC38 cells after different treatments. n = 5. Data are presented as mean ± SD. Statistical significance was calculated by a two-tailed unpaired Student's t-test. ***P < 0.001.


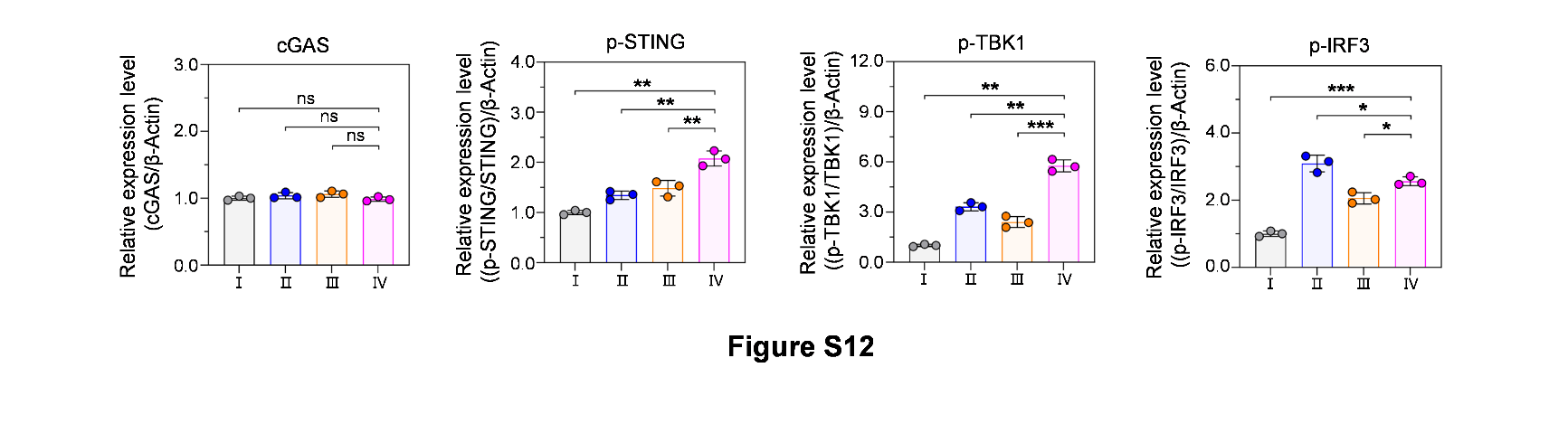
**Figure S12.** Normalized expression levels of cGAS，p-STING, p-TBK1, and p-IRF3 proteins in MC38 cells following different treatments. n = 3. Data are presented as mean ± SD. Statistical significance was calculated by a two-tailed unpaired Student's t-test. *P < 0.05, **P < 0.01, ***P < 0.001; ns: no significant difference.


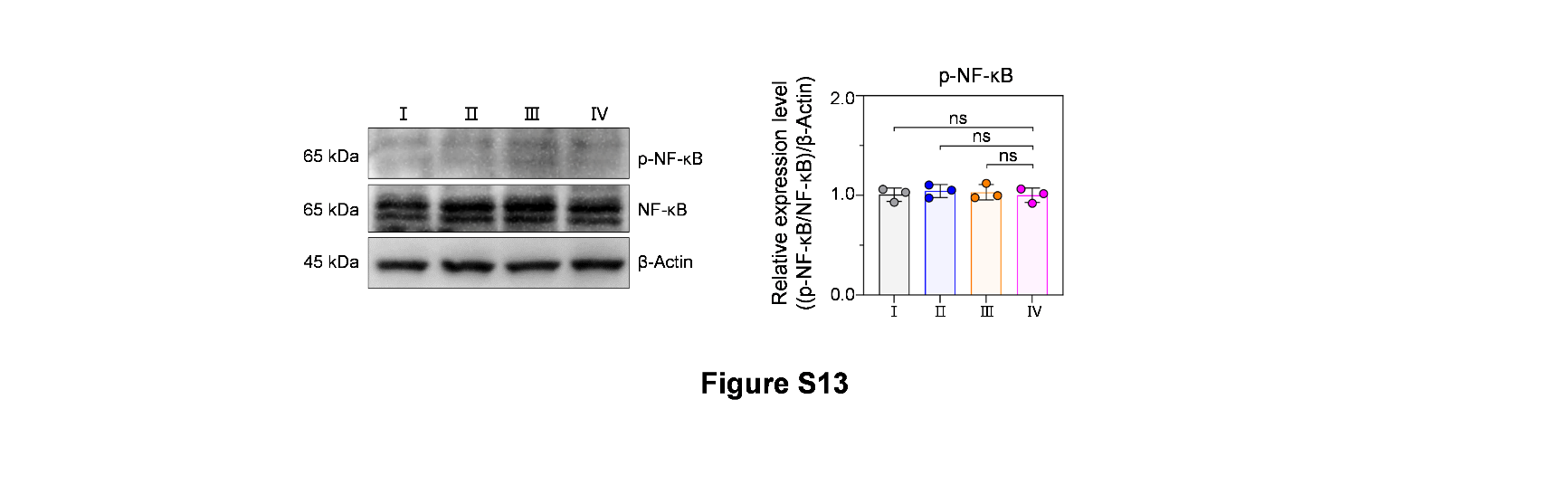
**Figure S13.** Western blot analysis of p-NF-κB expression in MC38 cells after the indicated treatments. n = 3. Data are presented as mean ± SD. Statistical significance was calculated by a two-tailed unpaired Student's t-test. ns: no significant difference.


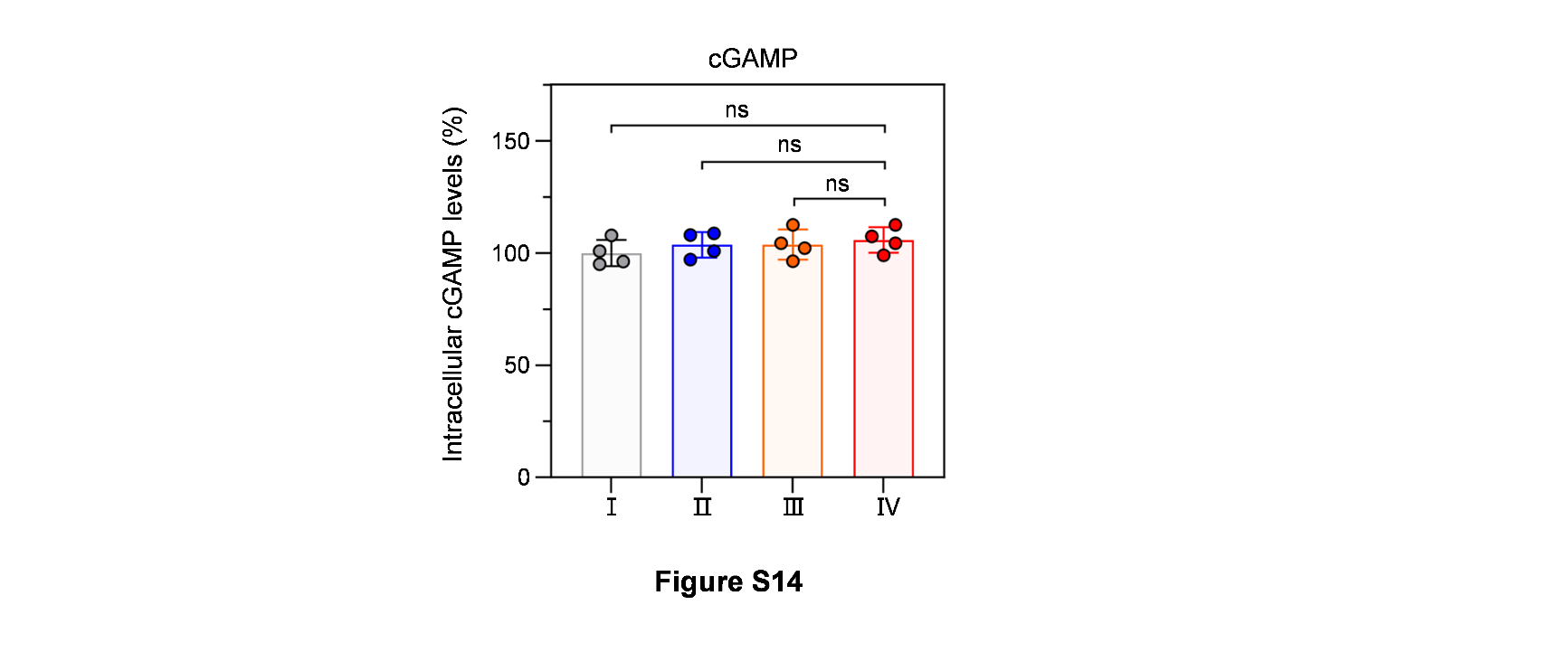
**Figure S14.** Intracellular cGAMP level in MC38 cells after different treatments. n = 4. Data are presented as mean ± SD. Statistical significance was calculated by a two-tailed unpaired Student's t-test. ns: no significant difference.


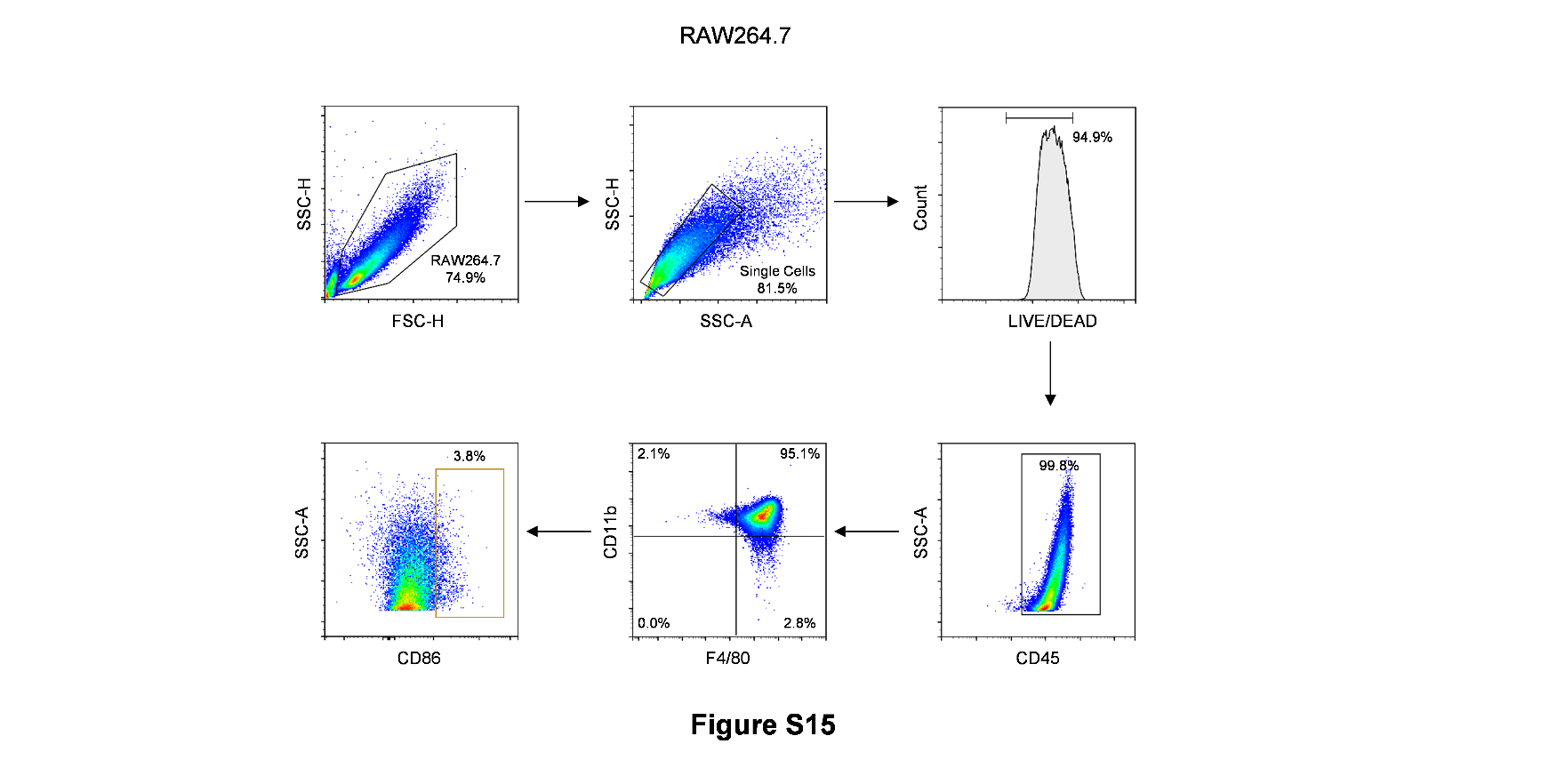
**Figure S15.** The gating strategy for the polarization of RAW264.7 cells after various treatments.


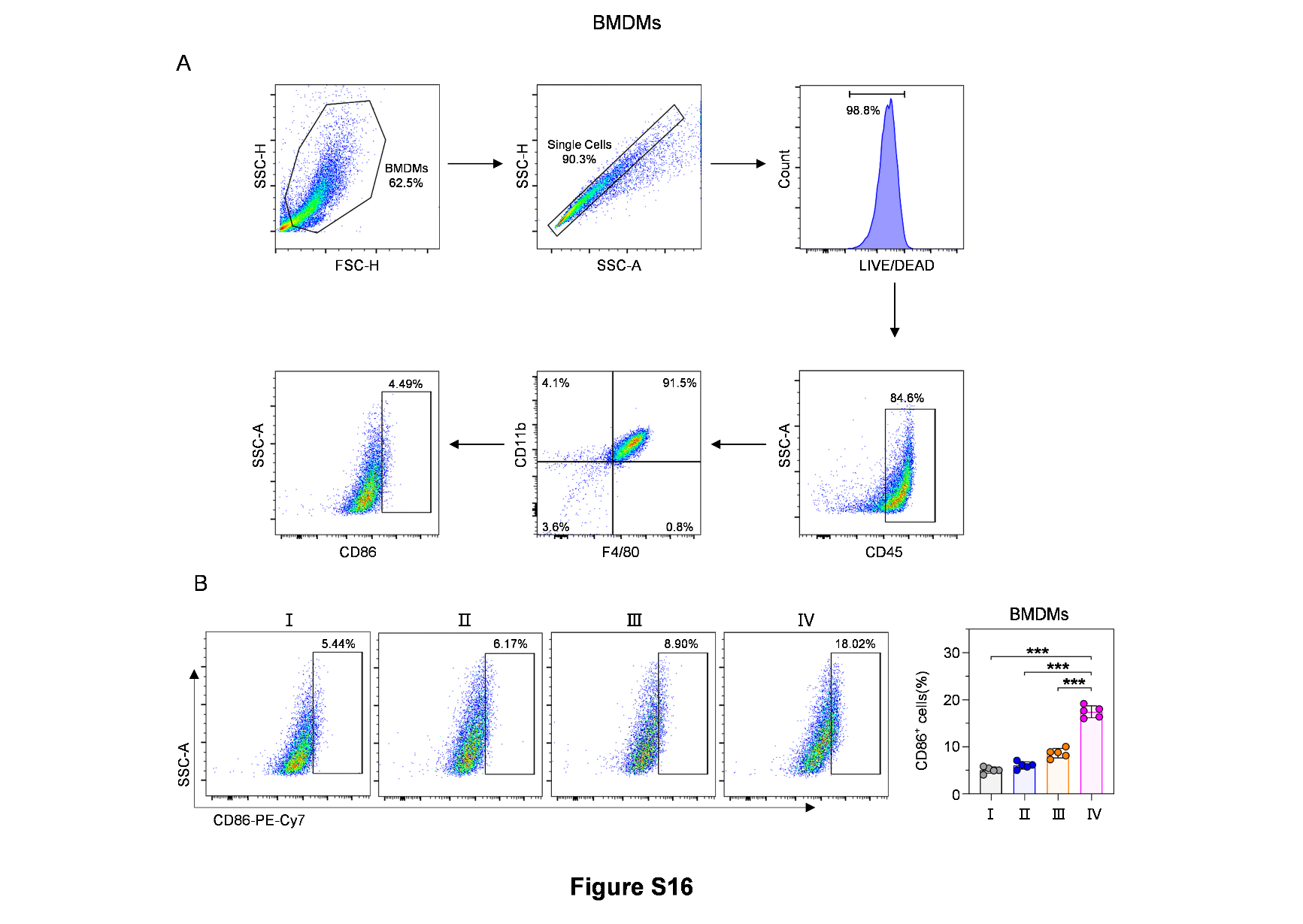
**Figure S16.** Flow cytometric and statistical analysis of the matured BMDMs after incubation with different medium. n = 5. Data are presented as mean ± SD. Statistical significance was calculated by a two-tailed unpaired Student's t-test. ***P < 0.001.


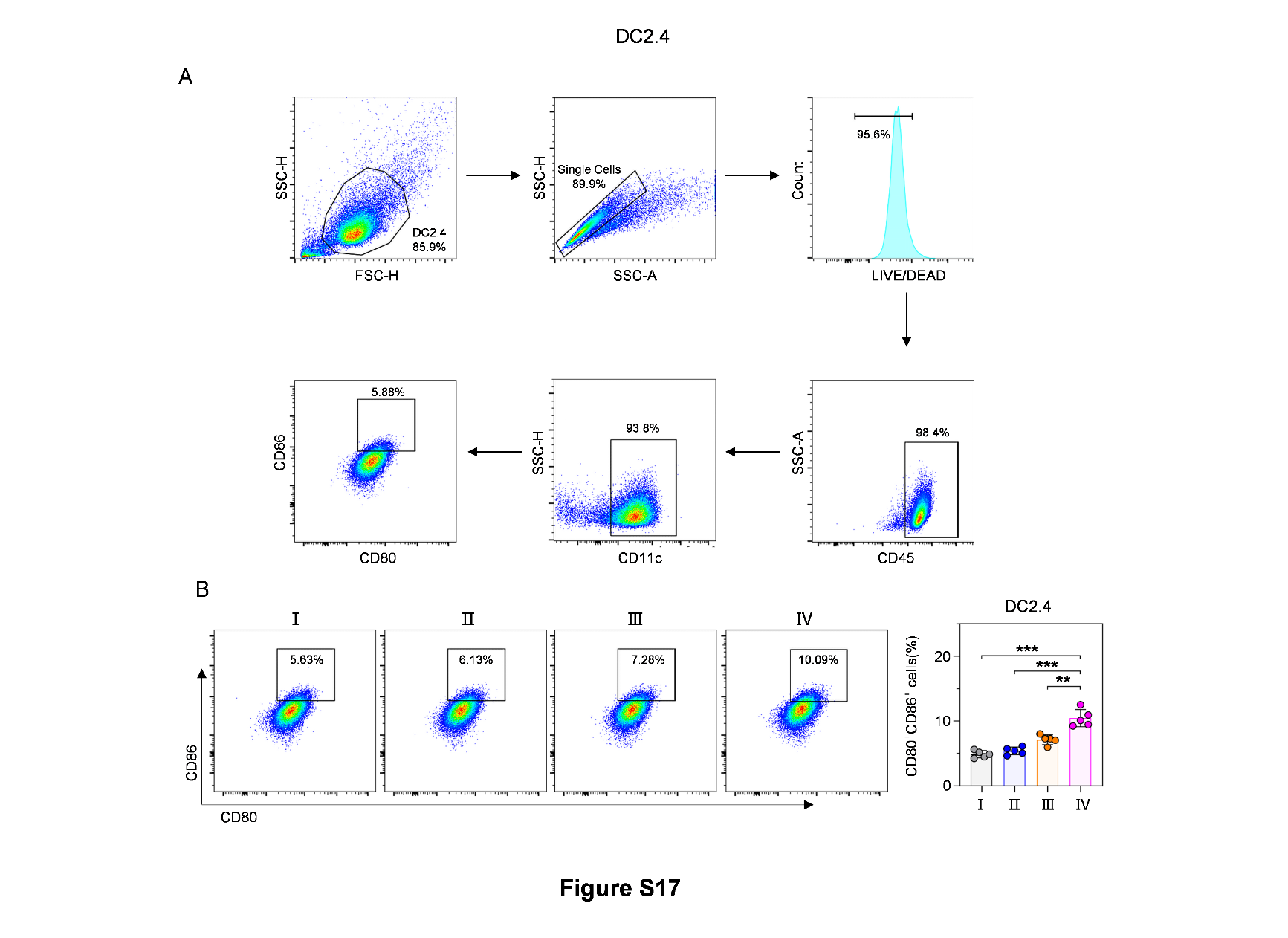
**Figure S17.** Flow cytometric and statistical analysis of the matured DC2.4 cells after incubation with different medium. n = 5. Data are presented as mean ± SD. Statistical significance was calculated by a two-tailed unpaired Student's t-test. **P < 0.01, ***P < 0.001.


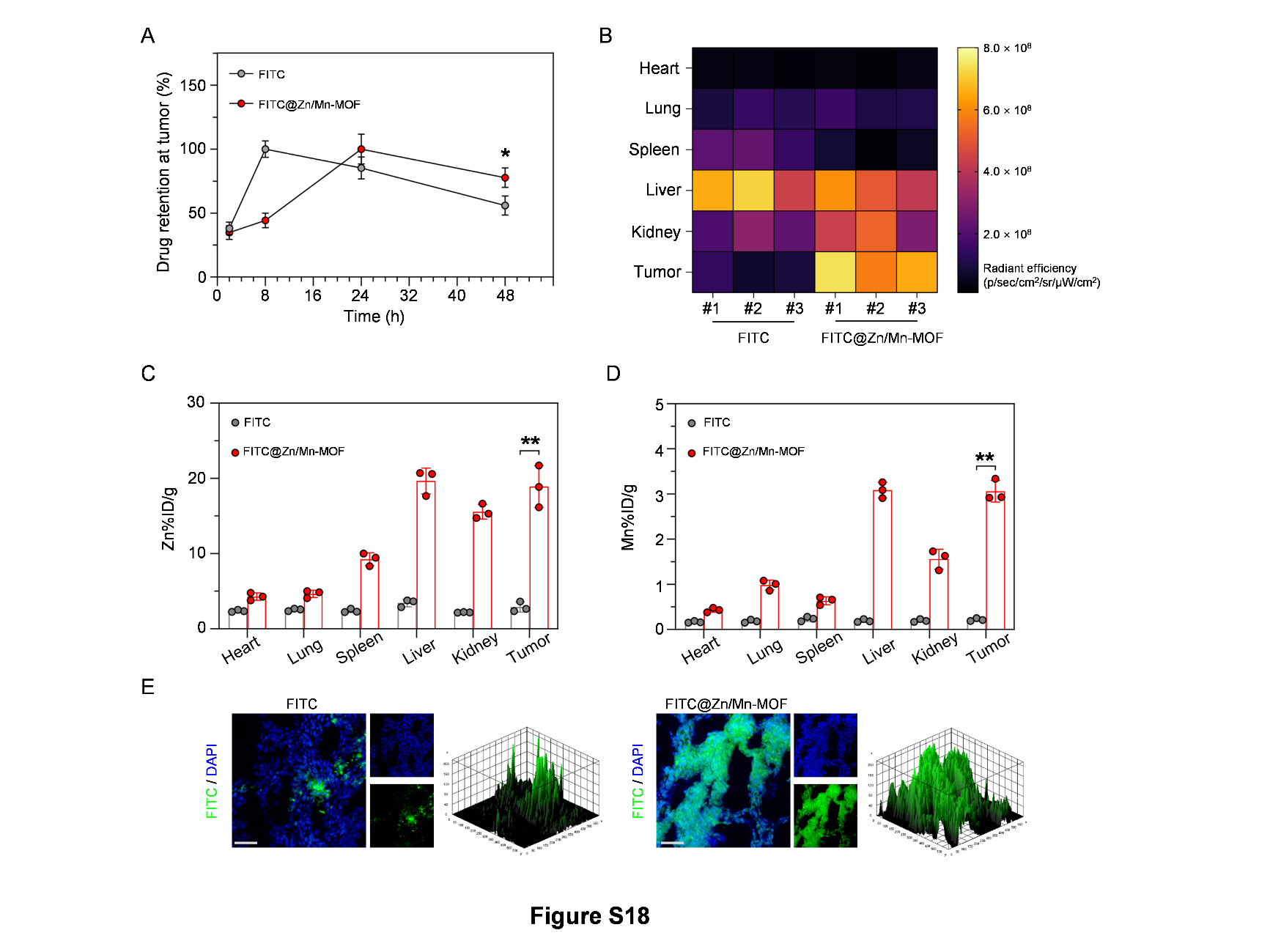
**Figure S18.** Biodistribution of FITC@Zn/Mn-MOF after intravenous injection.

(A, B) Quantitative analysis of fluorescence intensities in tumors and major organs excised from MC38 tumor-bearing mice 48 hours post-intravenous administration. n = 3. (C, D) Accumulated Mn and Zn ions in major organs and tumors after 48 hours post-intravenous injection. (E) Immunofluorescence staining analysis of FITC fluorescence signals in tumor tissues after indicated treatments. Scale bar: 100 μm. Data are presented as mean ± SD. Statistical significance was calculated by a two-tailed unpaired Student's t-test. *P < 0.05, **P < 0.01.


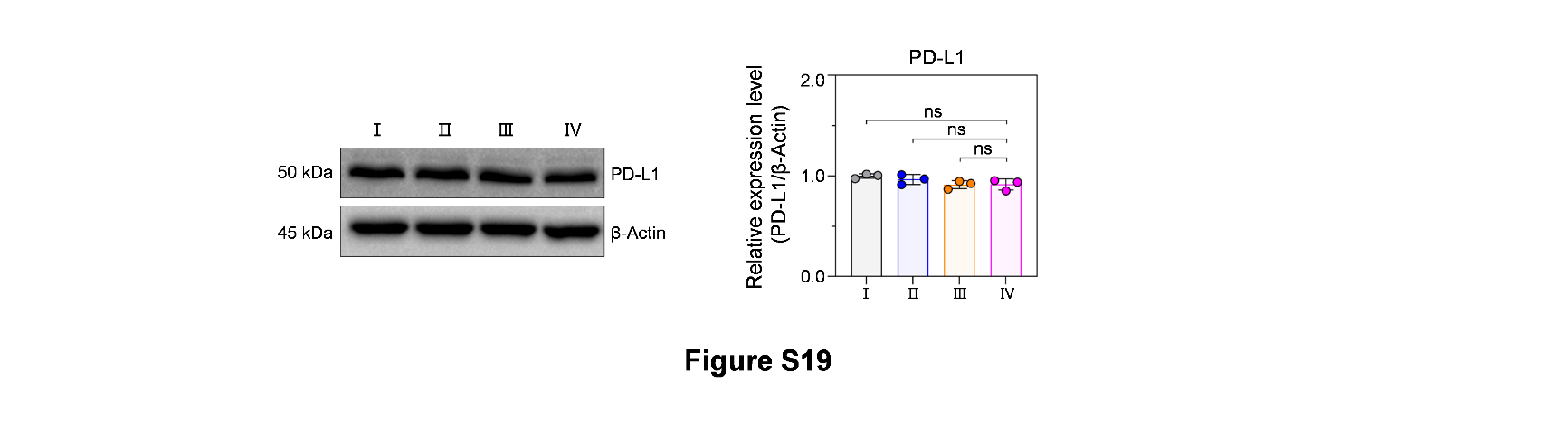
**Figure S19.** Western blot analysis of PD-L1 expression in MC38 cells after the indicated treatments. n = 3. Data are presented as mean ± SD. Statistical significance was calculated by a two-tailed unpaired Student's t-test. ns: no significant difference.


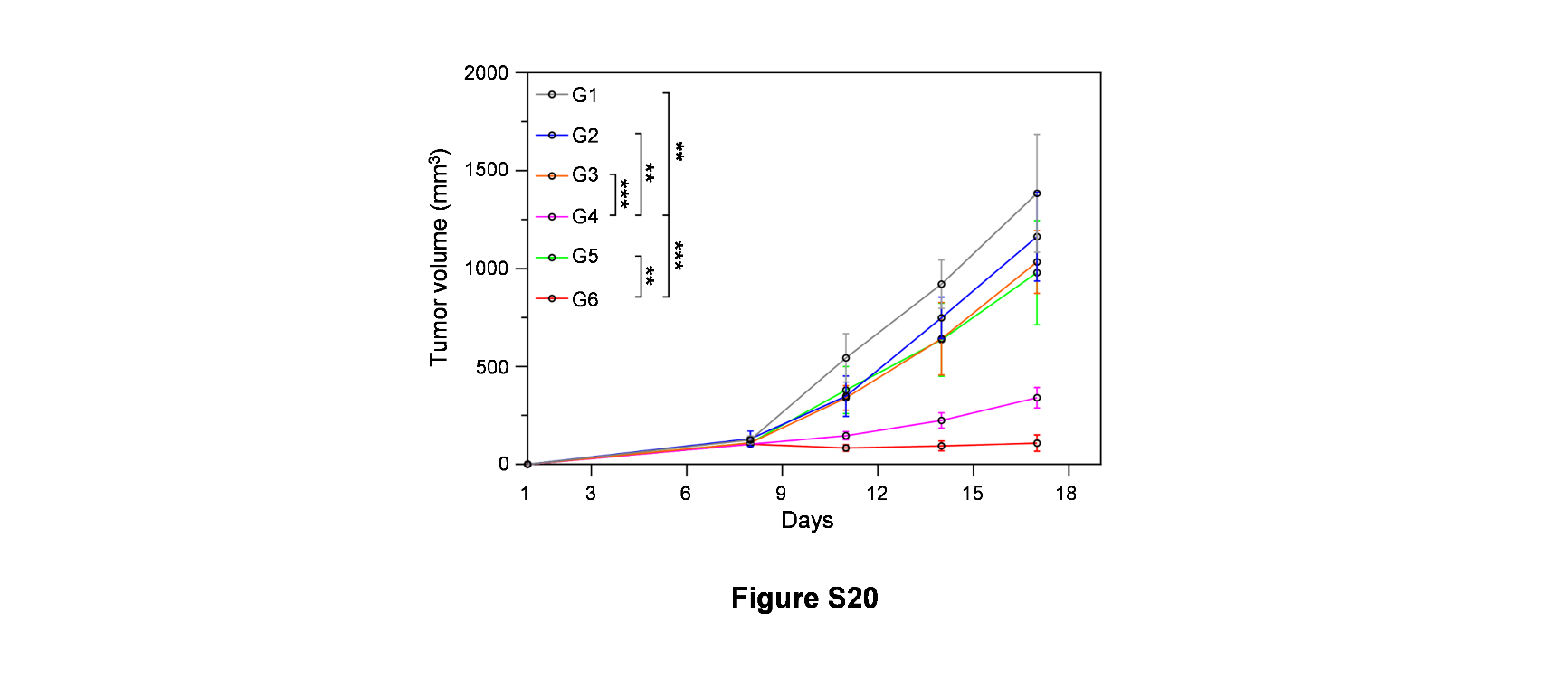


**Figure S20.** Growth curves of tumors in each mouse per group following various treatments. n = 5. Data are presented as mean ± SD. Statistical significance was calculated by a two-tailed unpaired Student's t-test. **P < 0.01, ***P < 0.001.


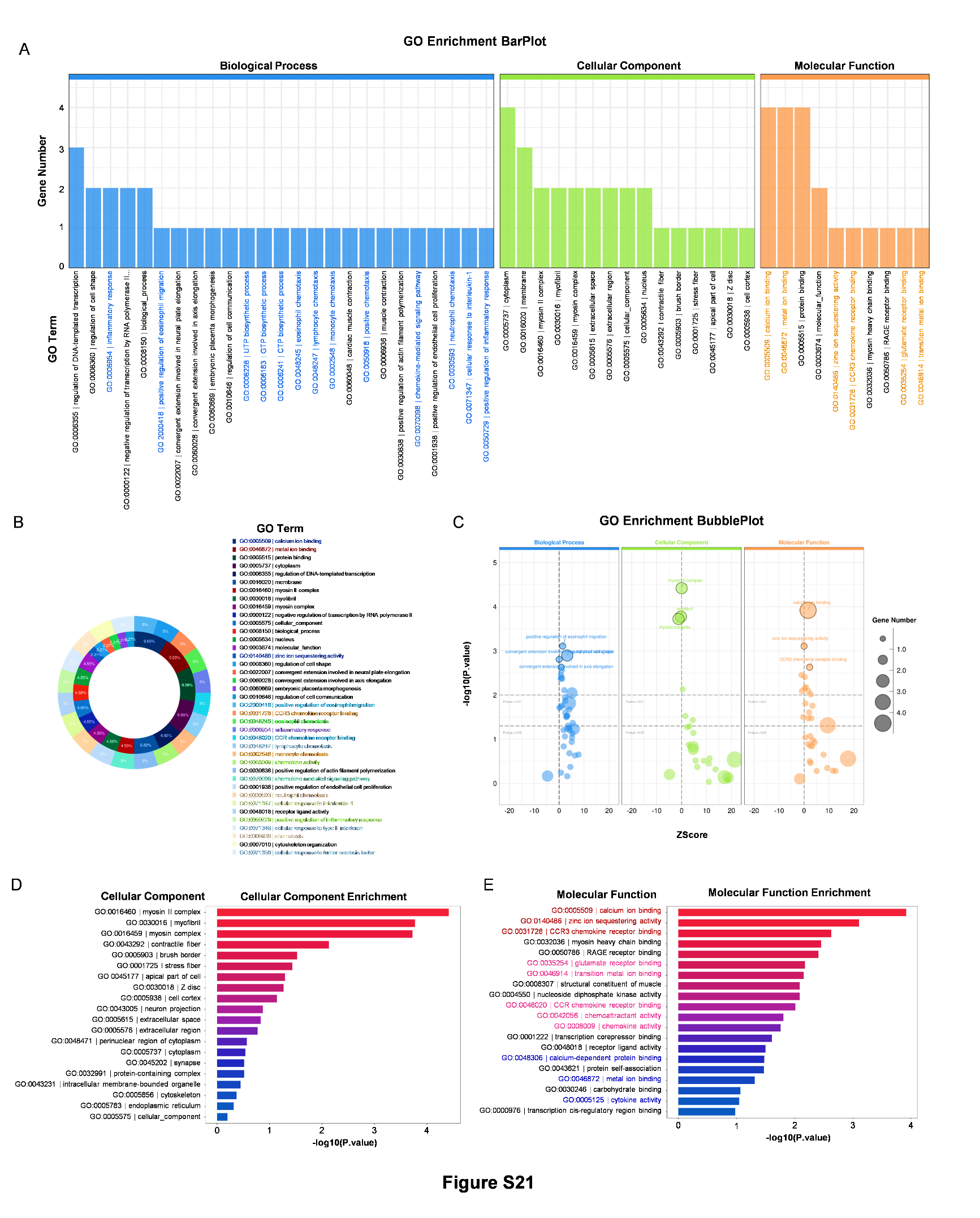


**Figure S21.** GO enrichment analysis comparing differentially expressed pathways in the PBS group versus the AMP@Zn/Mn-MOF groups. n = 4.


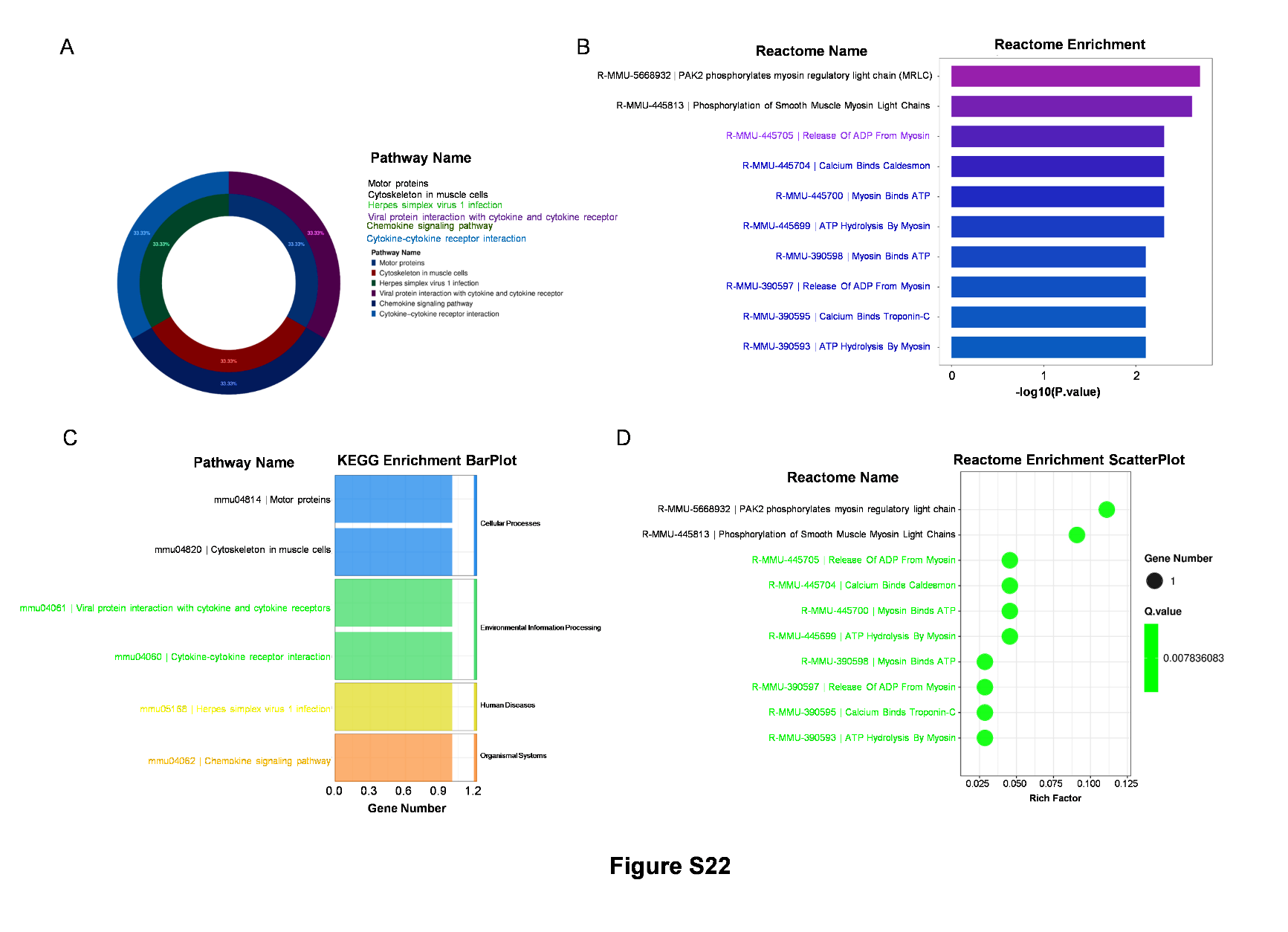
**Figure S22.** Kyoto Encyclopedia of Genes and Genomes (KEGG) pathway analysis of differentially expressed genes comparing the PBS group with the AMP@Zn/Mn-MOF group. n = 4.


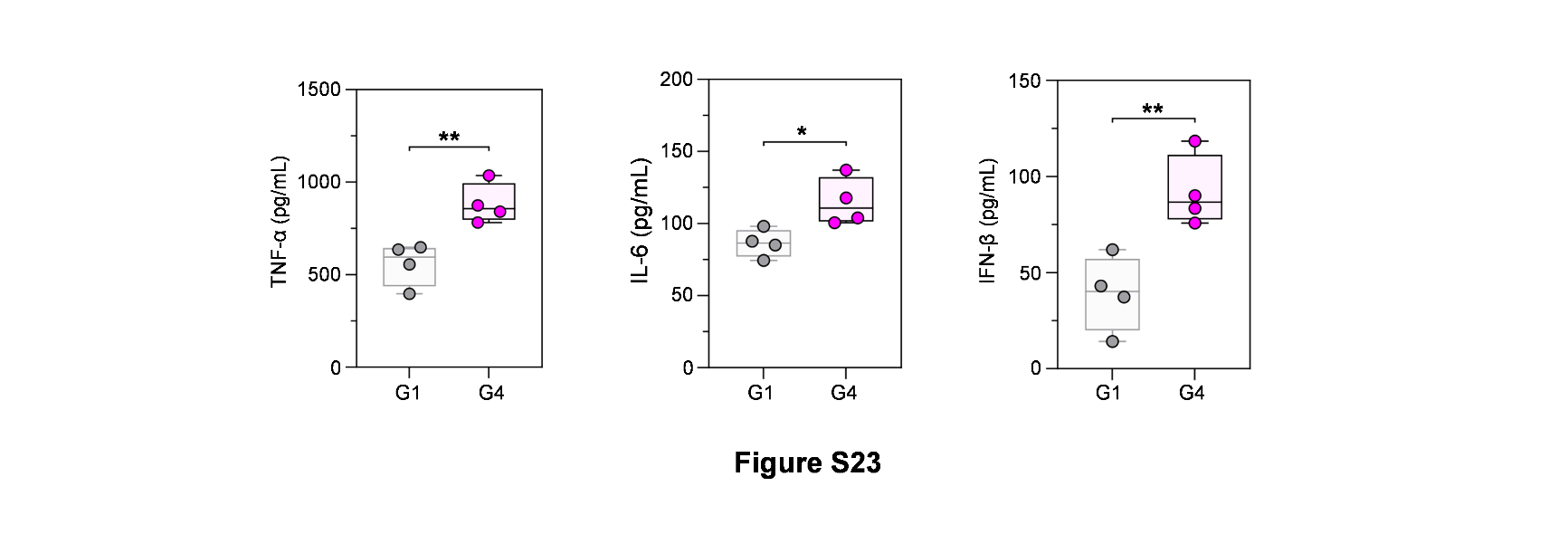
**Figure S23.** The serum levels of TNF-α, IL-6, and IFN-β in the PBS and AMP@Zn/Mn-MOF groups. n = 4. Data are presented as mean ± SD. Statistical significance was calculated by a two-tailed unpaired Student's t-test. *P < 0.05, **P < 0.01.


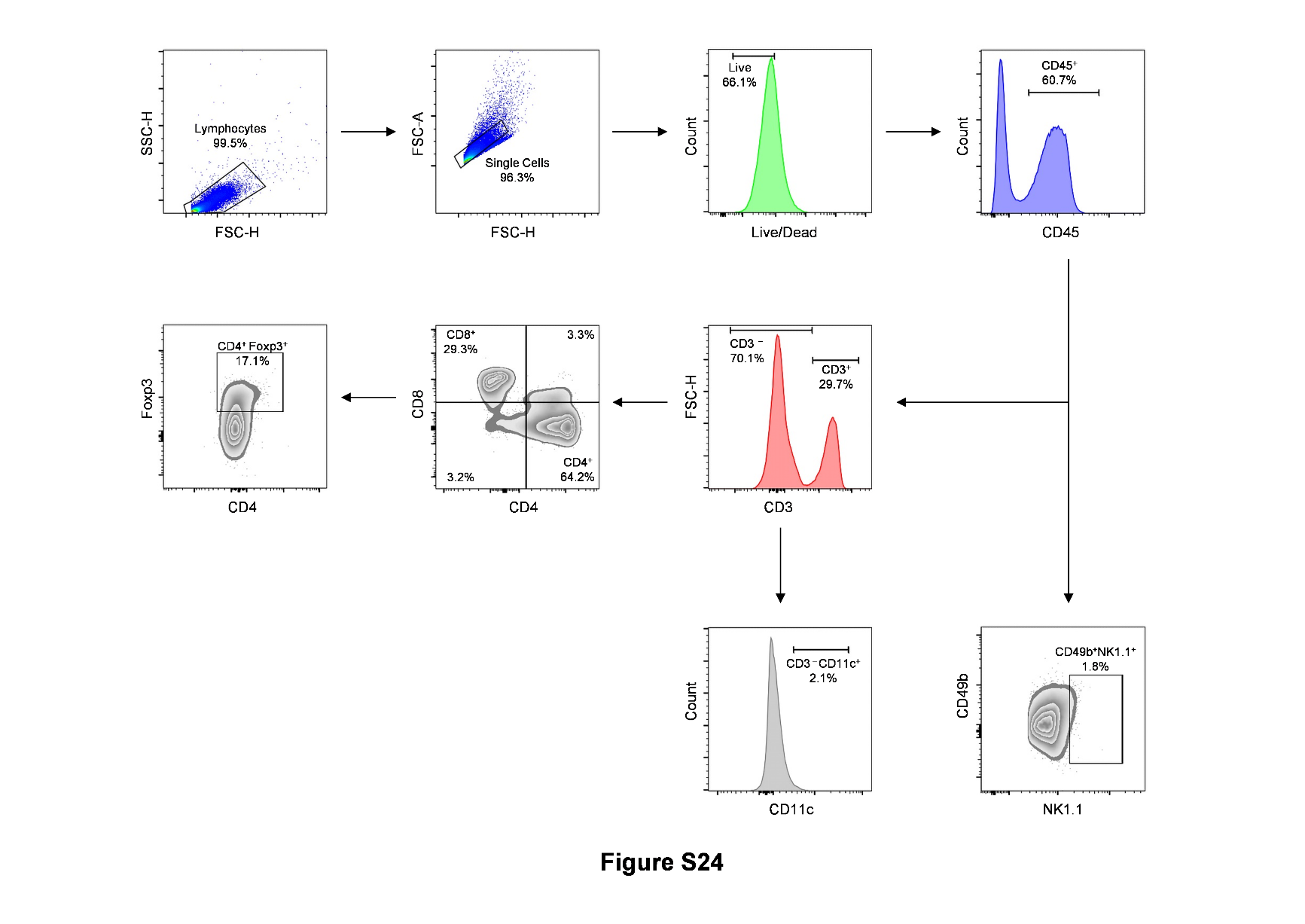
**Figure S24.** The gating strategy for tumor-infiltrating lymphocytes in tumor tissues after various treatments.


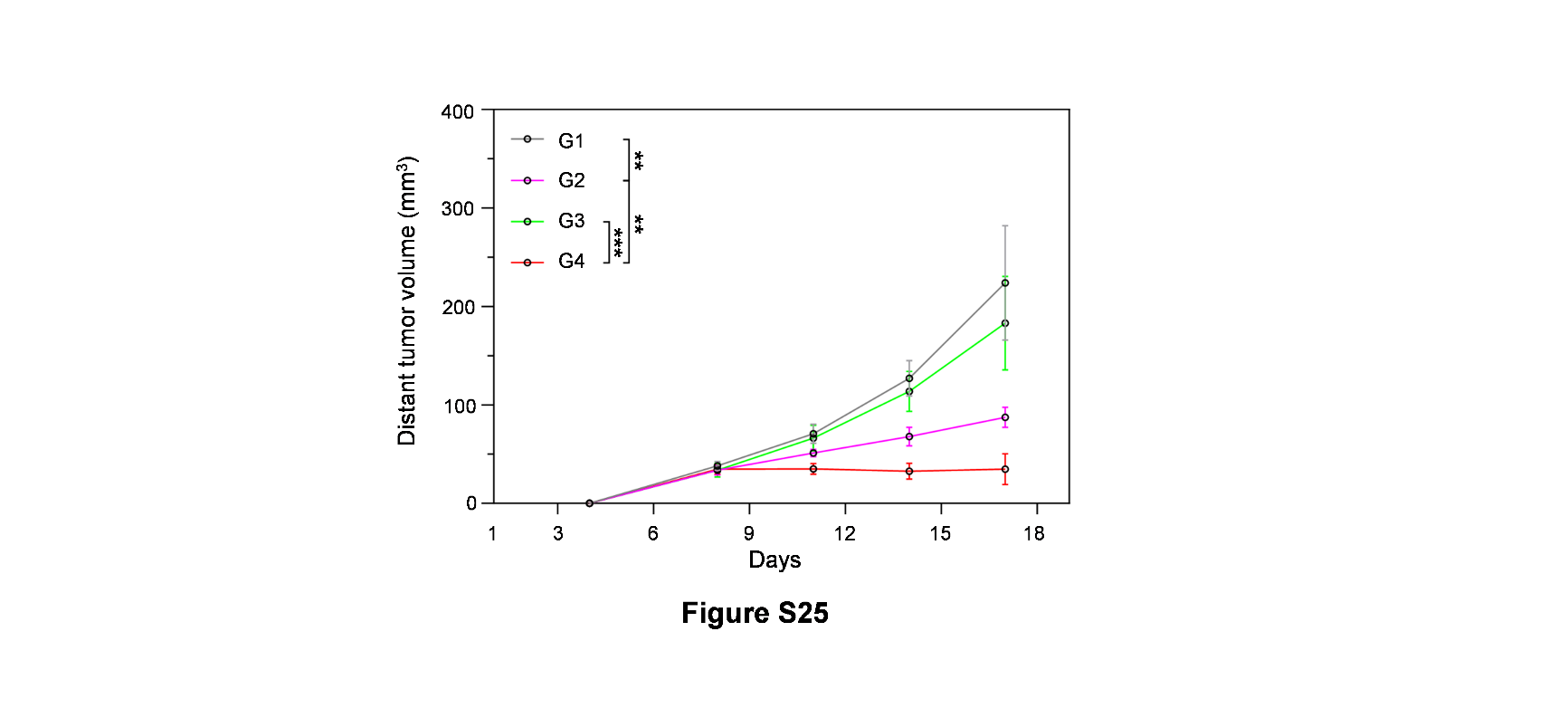
**Figure S25.** Growth curves of distant tumors in each mouse per group following various treatments. n = 5. Data are presented as mean ± SD. Statistical significance was calculated by a two-tailed unpaired Student's t-test. **P < 0.01, ***P < 0.001.


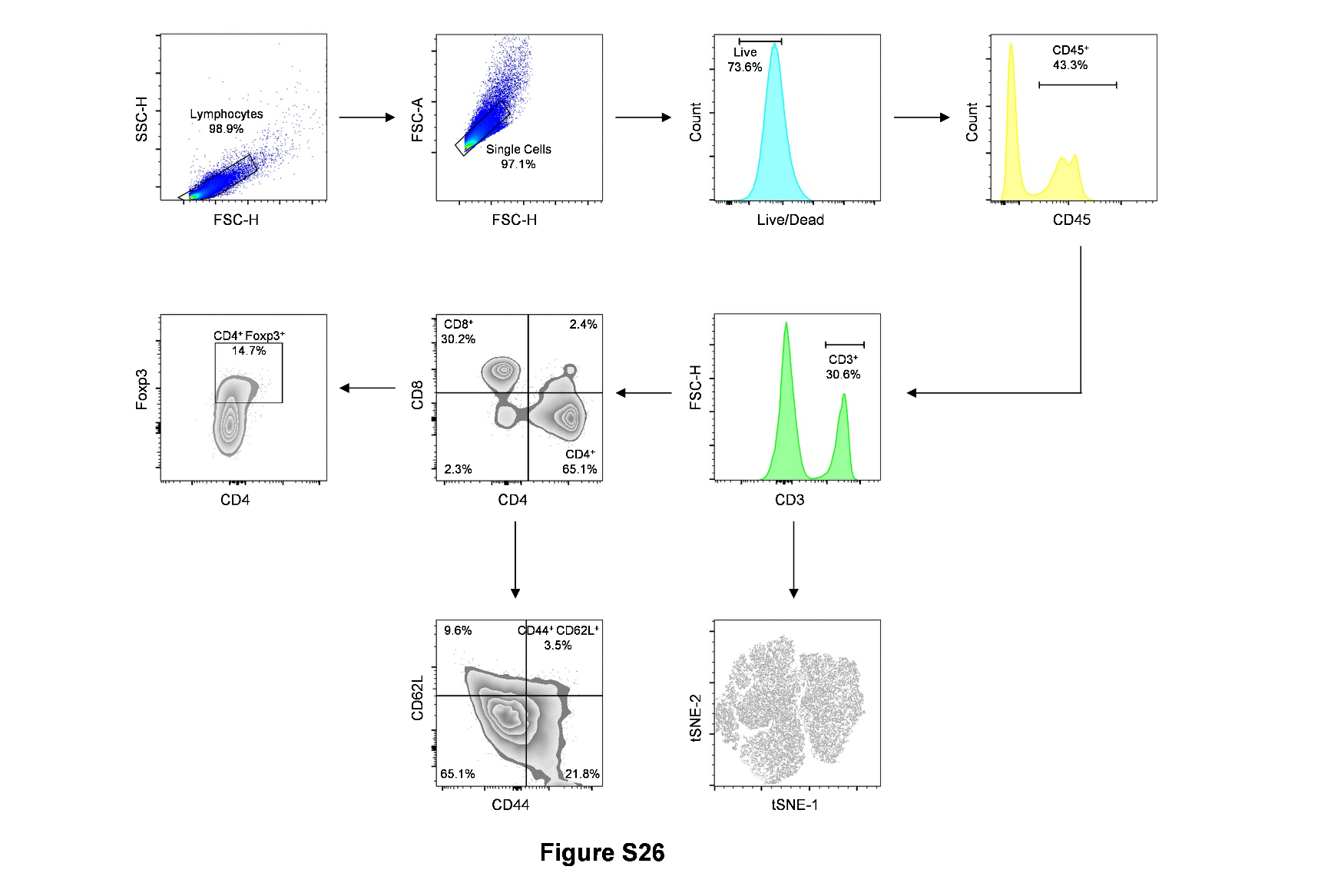
**Figure S26.** The gating strategy for tumor-infiltrating lymphocytes in distant tumor tissues after various treatments.


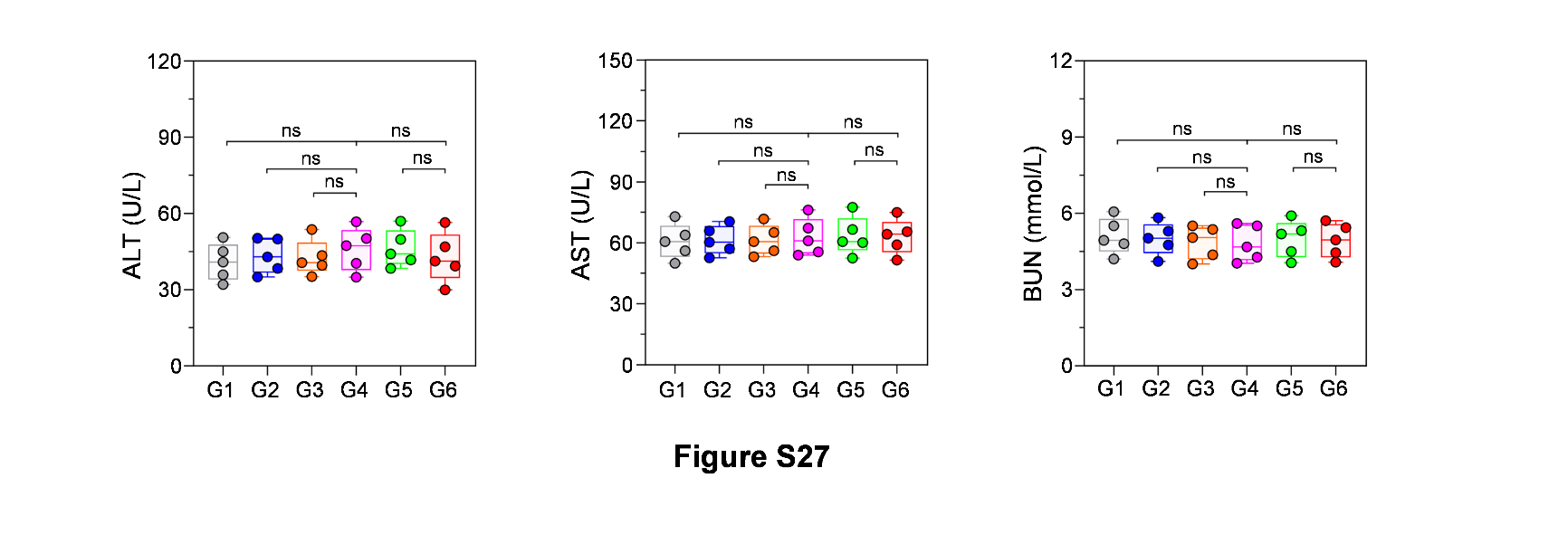
**Figure S27.** Serum biochemical analysis of liver function (AST and ALT) and kidney function (BUN) after indicated therapies. n = 5. Data are presented as mean ± SD. Statistical significance was calculated by a two-tailed unpaired Student's t-test. ns: no significant difference.


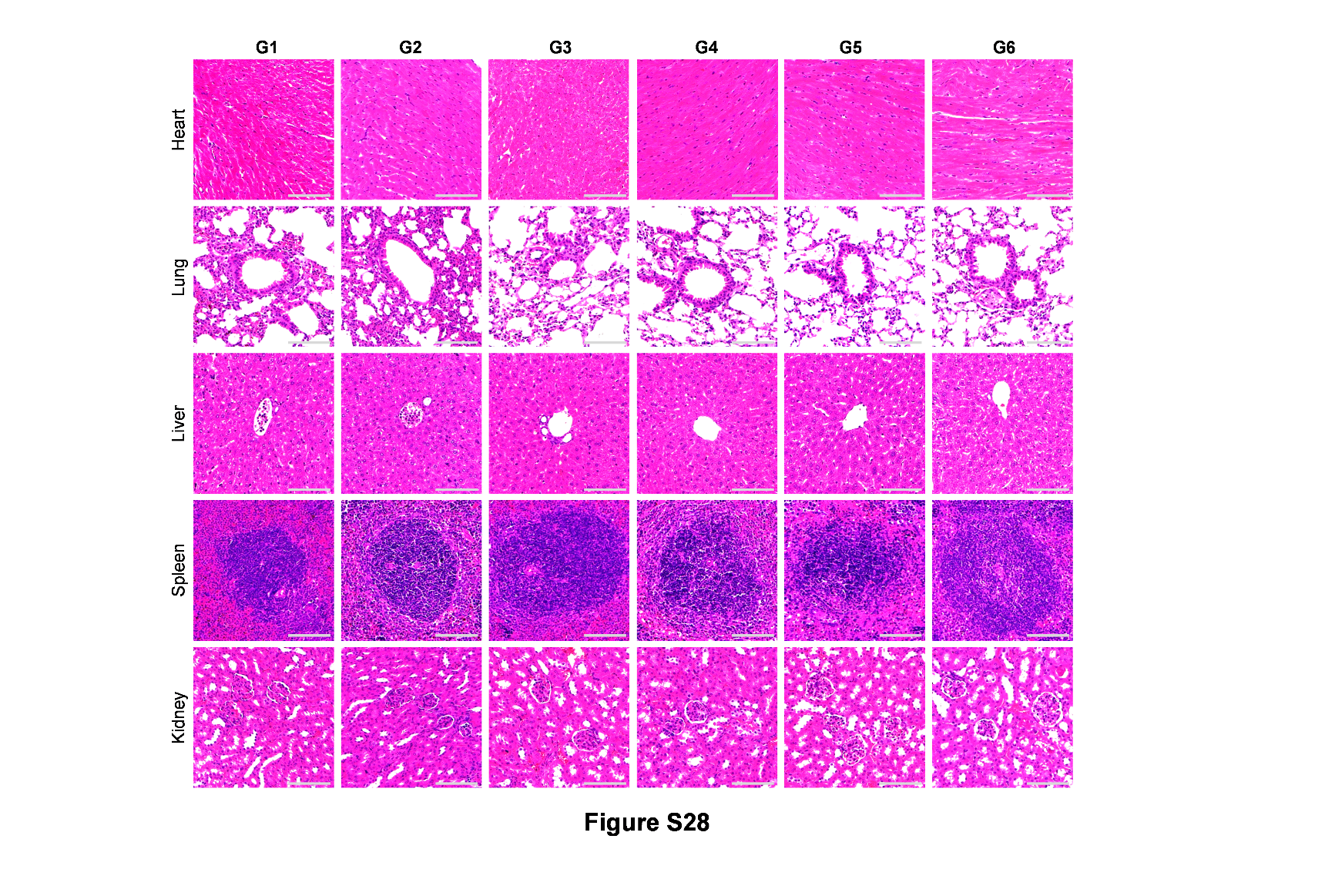
**Figure S28.** H&E staining images of major organs from MC38 tumor-bearing mice following the indicated treatments. Scale bar: 100 μm.


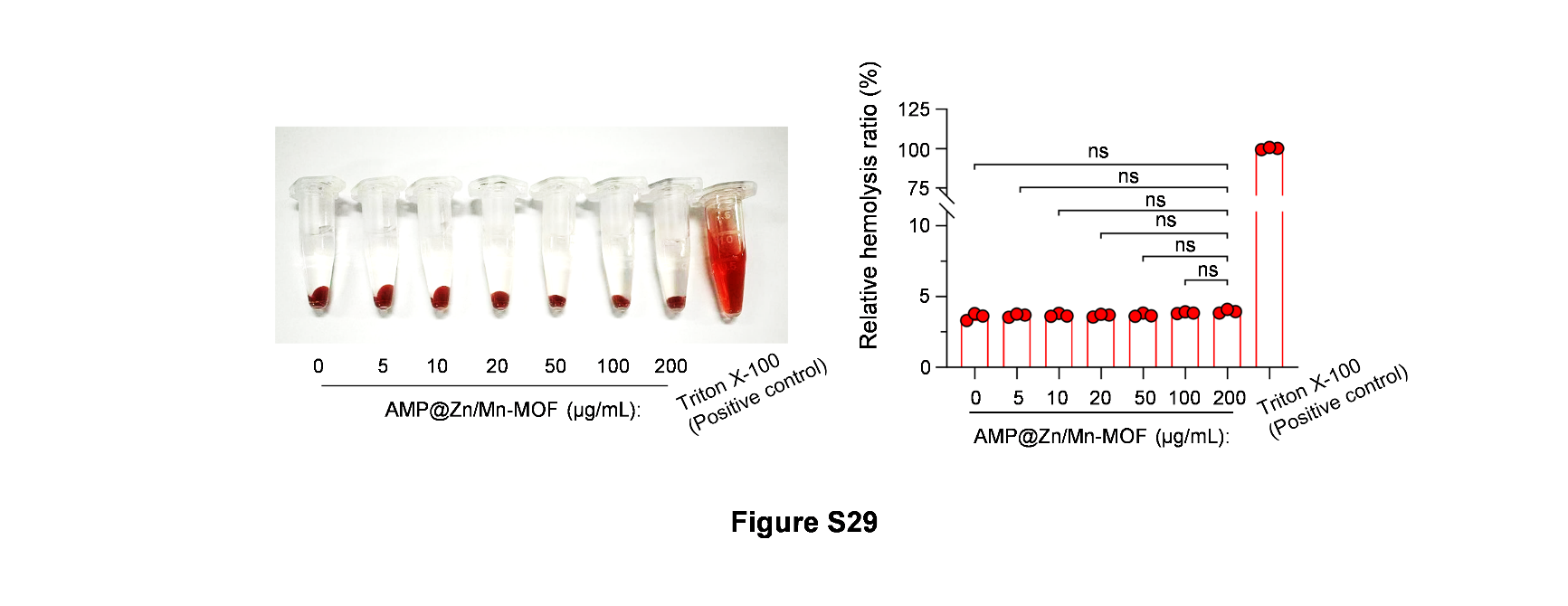
**Figure S29.** Hemolysis assay of AMP@Zn/Mn-MOF at different concentrations. 0.2% Triton X-100 was used as the positive control. n = 3. Data are presented as mean ± SD. Statistical significance was calculated by a two-tailed unpaired Student's t-test. ns: no significant difference.


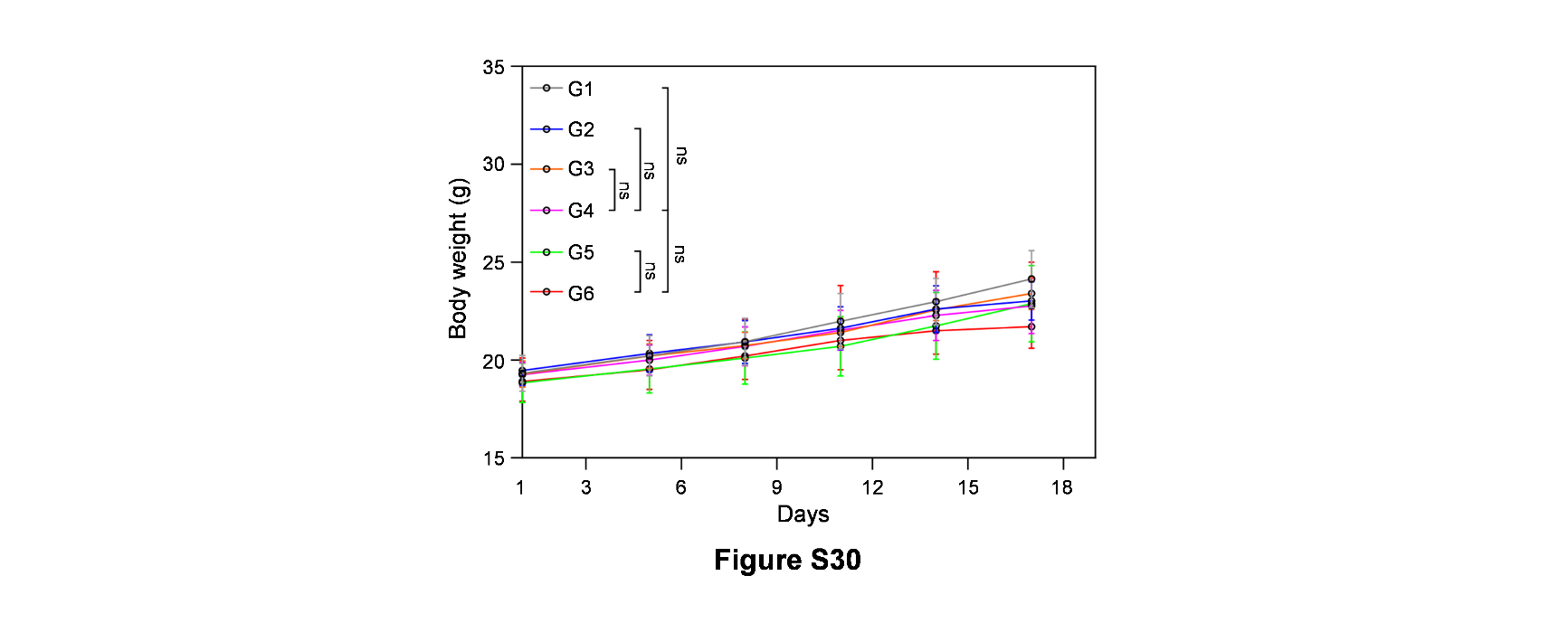
**Figure S30.** Body weight profiles of MC38 tumor-bearing mice over the observation period. n = 5. Data are presented as mean ± SD. Statistical significance was calculated by a two-tailed unpaired Student's t-test. ns: no significant difference.


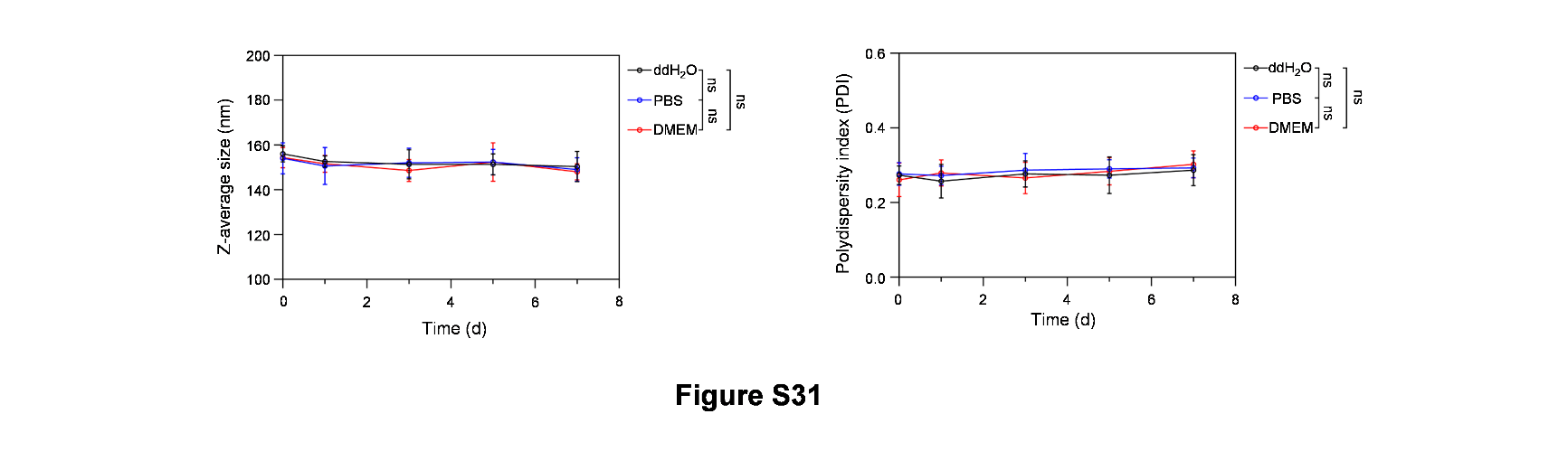


**Figure S31.** Colloidal stability of AMP@Zn/Mn-MOF. Data are presented as mean ± SD. Statistical significance was calculated by a two-tailed unpaired Student's t-test. ns: no significant difference.


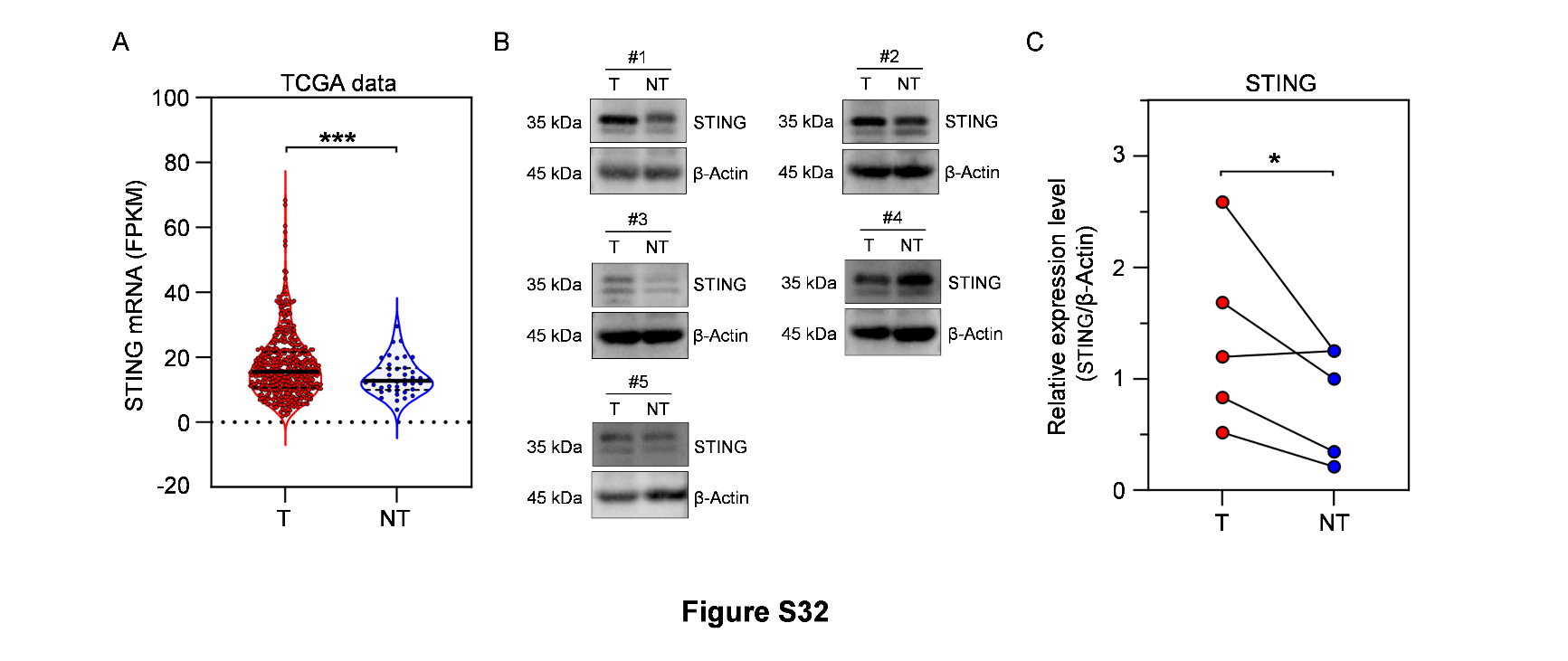


**Figure S32.** STING expression in TCGA data (tumor (T) tissue, n = 465; non-tumor (NT) tissue, n = 41) and human CC samples (n = 5). FPKM: fragments per kilobase of exon per million fragments mapped. Data are indicated with medians and quartiles (A). Statistical significance was calculated by a two-tailed unpaired Student's t-test or ratio paired t-test. *P < 0.05, ***P < 0.001.


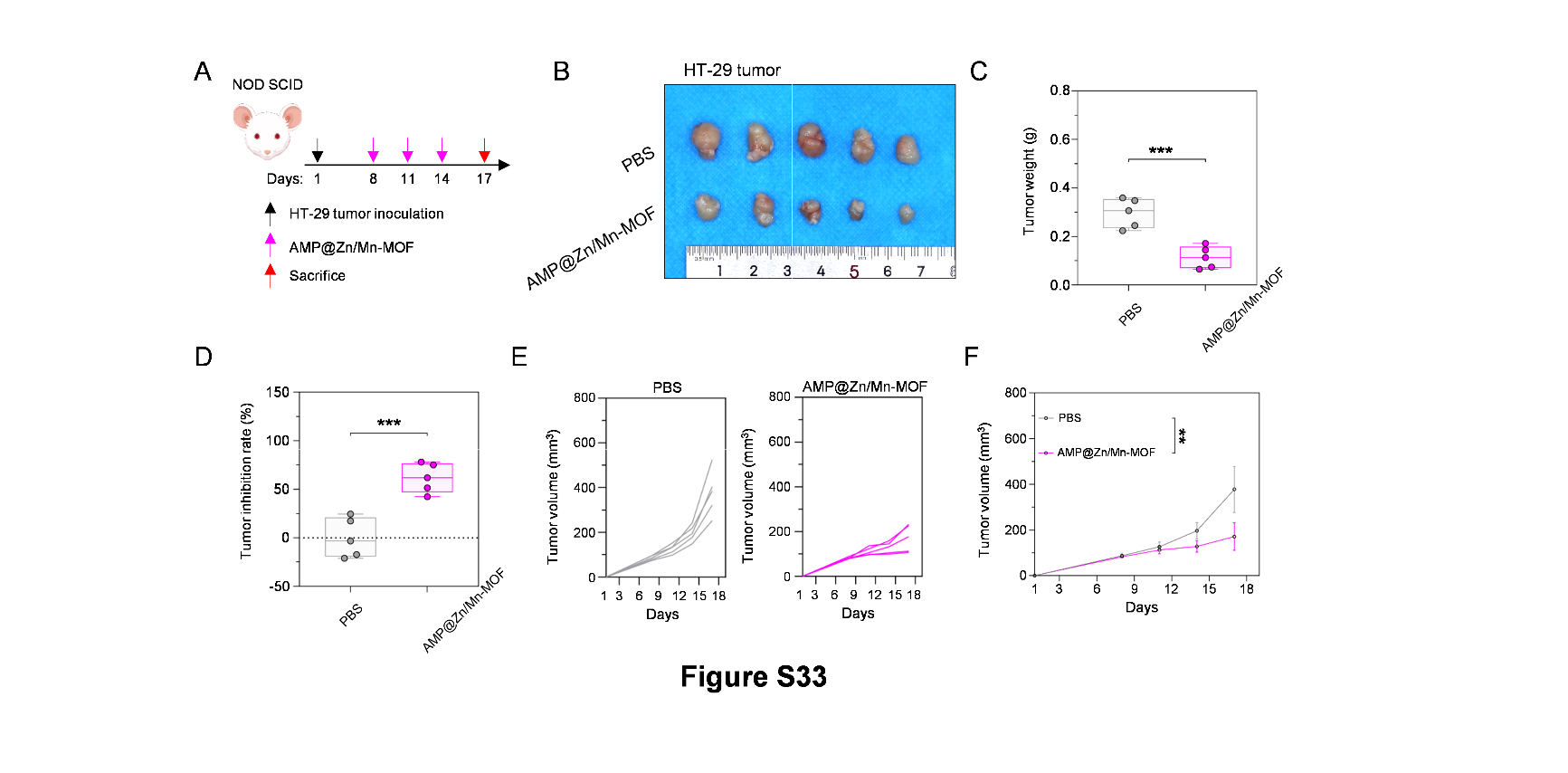
**Figure S33.** *In vivo* antitumor effect of AMP@Zn/Mn-MOF in an HT-29 xenograft model. (A) Schematic illustration of the *in vivo* antitumor experimental protocol. Created in Figdraw. (B) Digital images of tumors resected from each mouse following different treatments. (C) Profile of tumor weight in mice subjected to different treatments. n = 5. (D) Tumor inhibition rate in mice following different treatments. n = 5. (E, F) Tumor growth curves for each mouse in each group after different treatments. n = 5. Data are presented as mean ± SD. Statistical significance was calculated by a two-tailed unpaired Student's t-test. **P < 0.01, ***P < 0.001.


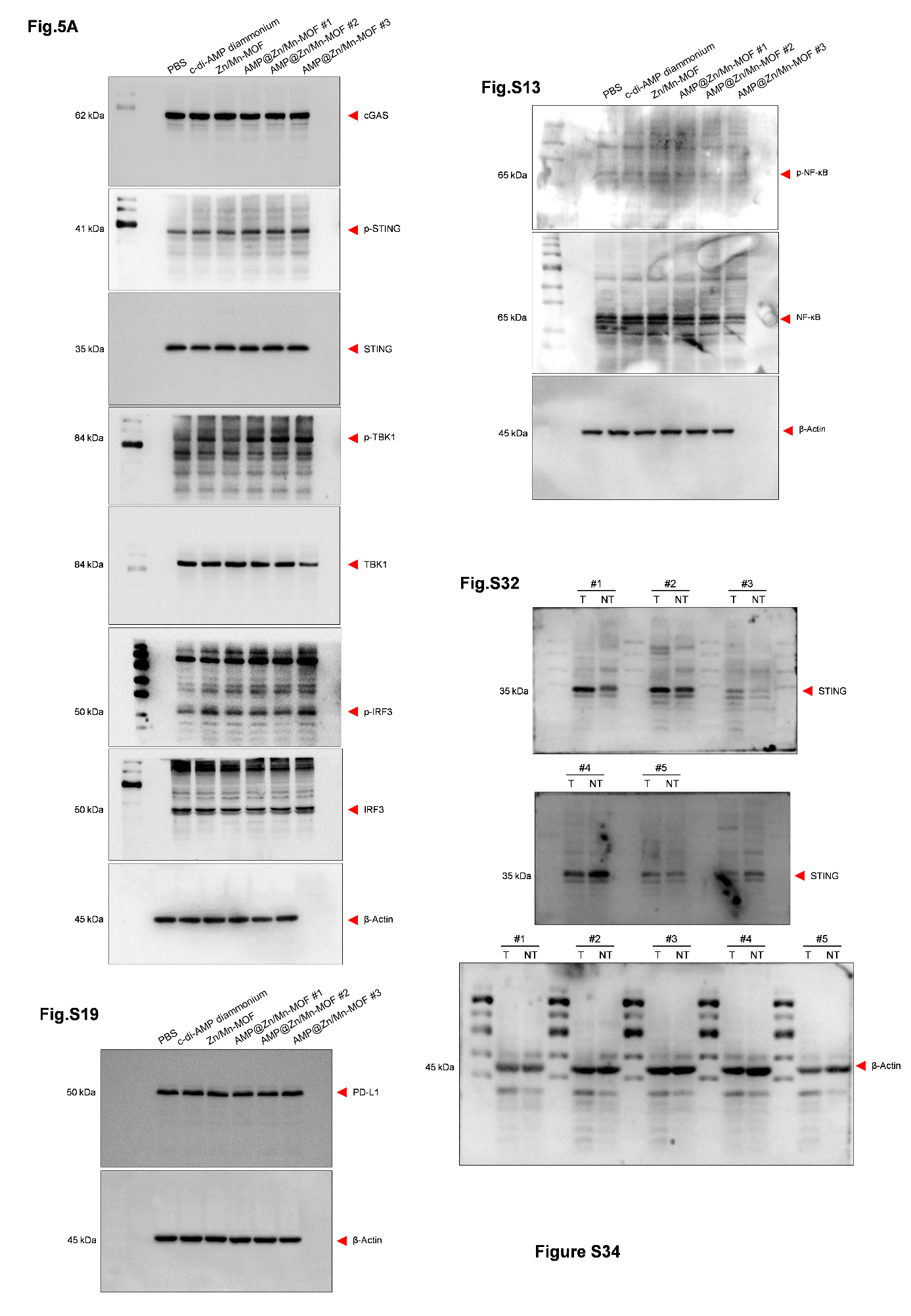


**Figure S34.** Unprocessed western blots.

**Supplementary Table 1.**

Chemicals and reagents used in the study.

| **Chemicals/reagents** | **Manufacturer** | **CAS or Catalog#:** |
| --- | --- | --- |
| Zn(NO_3_)_2_•6H_2_O | Aladdin | 10196-18-6 |
| Mn(NO_3_)_2_•4H_2_O | Aladdin | 20694-39-7 |
| 2-methylimidazole | Sigma-Aldrich | 693-98-1 |
| DSPE-PEG_2000_-NHS | Macklin | 1445723-73-8 |
| c-di-AMP diammonium | MedChemExpress | HY-12326B |
| cGAS antibody | Cell Signaling Technology | 31659S |
| STING antibody | Cell Signaling Technology | 13647S |
| p-STING antibody | Cell Signaling Technology | 72971S |
| TBK1 antibody | Cell Signaling Technology | 3504S |
| p-TBK1 antibody | Cell Signaling Technology | 29047S |
| IRF3 antibody | Cell Signaling Technology | 4302S |
| p-IRF3 antibody | Cell Signaling Technology | 5483S |
| β-actin antibody | Cell Signaling Technology | 4970S |
| PD-L1 antibody | Cell Signaling Technology | 13684T |
| NF-κB | Cell Signaling Technology | 8242S |
| p-NF-κB | Cell Signaling Technology | 3033S |
| Phosphatase inhibitor cocktail | Sigma-Aldrich | P5726 |
| Protease inhibitor cocktail | Sigma-Aldrich | P8340 |
| RIPA Lysis Buffer | Beyotime | P0013B |
| PVDF Membrane | Merck Millipore | IPVH00010 |
| BCA protein assay kit | Beyotime | P0010 |
| BSA | Beyotime | ST023 |
| Anti-rabbit IgG, HRP-linked Antibody | Cell Signaling Technology | 7074 |
| Anti-mouse IgG, HRP-linked Antibody | Cell Signaling Technology | 7076 |
| ECL | NCM Biotech | P10300 |
| FITC | Aladdin | 27072-45-3 |
| MB | Aladdin | 61-73-4 |
| DTNB | Aladdin | 69-78-3 |
| GSH | Aladdin | 70-18-8 |
| H_2_O_2_ | Aladdin | 7722-84-1 |
| DMEM, high glucose | Gibco | 10566016 |
| RPMI 1640 | Gibco | 61870127 |
| Fetal bovine serum (FBS) | Gibco | 10099141 |
| Trypsin-EDTA (0.05%) | Gibco | 25300062 |
| Penicillin/streptomycin | Gibco | 15140122 |
| B27 | Gibco | A1486701 |
| M-CSF | Sigma-Aldrich | SRP3221 |
| Annexin V-FITC/PI Apoptosis Detection Kit | BD Biosciences | 556547 |
| ThiolTracker | Invitrogen | T10095 |
| Cell Counting Kit-8 | Dojindo | CK04 |
| Cytotoxicity LDH Assay Kit | Dojindo | CK12 |
| Extracellular ATP Assay Kit | Dojindo | E299 |
| MitoPeDPP | Dojindo | M466 |
| LysoPrime Deep Red | Dojindo | L264 |
| MitoBright LT Red | Dojindo | MT11 |
| DCFH-DA | Beyotime | S1105 |
| DAPI | Sigma-Aldrich | D9542 |
| Hoechst 33342 | Sigma-Aldrich | 875756-97-1 |
| CD8 antibody | Abcam | ab217344 |
| CRT antibody | Abcam | AB2907 |
| HMGB-1 antibody | Abcam | Ab190377 |
| Goat anti-Rabbit IgG (H+L) Cross-Adsorbed Secondary Antibody, Alexa Fluor™ 488 | Invitrogen | A-11070 |
| Goat anti-Rabbit IgG (H+L) Highly Cross-Adsorbed Secondary Antibody, Alexa Fluor™ 546 | Invitrogen | A-11035 |
| Calcein-AM/ PI | Beyotime | C1371S |
| GSH assay kit | Beyotime | S0052 |
| TRIzol reagent | Invitrogen | 15596018 |
| iScript cDNA synthesis kit | Bio-Rad | 1708840 |
| SsoFast^TM^ EvaGreen Supermix | Bio-Rad | 1725201 |
| Crystal Violet | Absin | abs44122325 |
| Transwell | Costar | 3422 |
| *In vivo* MAb anti-mouse PD-L1 antibody | Bio X Cell | BE0101 |
| FcX™ PLUS | BioLegend | 156604 |
| anti-CD45 | BioLegend | 103110 |
| anti-CD3 | BioLegend | 100206 |
| anti-CD4 | BioLegend | 100406 |
| anti-CD8a | BioLegend | 100714 |
| anti-CD44 | BioLegend | 103047 |
| anti-CD62L | BioLegend | 104453 |
| anti-CD49b | BioLegend | 108910 |
| anti-NK1.1 | BioLegend | 108748 |
| anti-CD11c | BioLegend | 117353 |
| anti-CD86 | BioLegend | 105014 |
| anti-CD11b | BioLegend | 101206 |
| anti-F4/80 | BioLegend | 123110 |
| anti-CD80 | BioLegend | 104705 |
| LIVE/DEAD Fixable Violet Deadcell stain Kit | Invitrogen | L34963 |
| anti-FOXP3 antibody | eBioscience | 25-5773-82 |
| anti-Ki67 antibody | Invitrogen | 14-5698-82 |
| Dako REAL™ EnVision™ Detection System | Dako | K5007 |
| H&E staining Kit | Absin | abs9217 |
| Tissue-Tek® O.C.T. Compound | SAKURA | 4583 |

**Supplementary Table S2.**

Primers for RT-qPCR.

| **Name** | **Species** | **Gene ID** | **Forward and Reverse Primer Sequence**  **(5' -> 3')** |
| --- | --- | --- | --- |
| *Ifnb1(IFN-β)* | *Mus musculus* | 15977 | CGATGTGGTCAAGATCCAGGT |
|  |  |  | TGGCCTCTACATAGCCCACTT |
| *Actb(β-actin)* | *Mus musculus* | 11461 | GGCTGTATTCCCCTCCATCG |
|  |  |  | CCAGTTGGTAACAATGCCATGT |
